# Supplementary material for: Incidence and Risk Factors for the Development of Stress Fractures in Military Recruits and Qualified Personnel: A Systematic Review
Source: Int J Environ Res Public Health. 2025 Nov 20;22(11):1760. doi: 10.3390/ijerph22111760 (PMC12652357; doi:10.3390/ijerph22111760)
Supplement: Supplementary file 1 [file ijerph-22-01760-s001.zip › Supplementary Material File S3 - Excluded Articles with Reasons.pdf]

Supplementary Material S3: EXCLUDED ARTICLES WITH REASONS

**Reason for exclusion**	**References**
Nil incidence/ prevalence/ risk factors or exposures reported	[1-65]
Published prior to 2000	[66-150]
Did not meet required level of evidence (i.e. case-control, cross-sectional design, theses, case series, or hospital-based data)	[151-286]
Duplicate	[287-294]
Not original research or abstract	[295-362]
Unpaid/non-occupational/non-military/elderly/non-human populations	[363-389]
Research protocol & conference abstracts	[390-399]
Pre-existing medical condition	[400-423]
RMA criterion (fracture cannot be verified)	[424-472]
No valid incidence or risk factor data, or data that can be independently validated	[473]
Articles including only traumatic fractures	[474-496]

1. Baker BS, Buchanan SR, Black CD, Bemben MG, Bemben DA. Bone, Biomarker, Body Composition, and Performance Responses to 8 Weeks of Reserve Officers' Training Corps Training. J Athl Train. 2022;57:571-580.
2. Barnes DR, Yoganandan N, Moore J, Humm J, Pintar F, Loftis KL. Quantifying the Effect of Pelvis Fracture on Lumbar Spine Compression during High-rate Vertical Loading. SAE Int. 2022;65: 189-216.
3. Chung BH, Shaw KA, Burke JS, Jackson KL, Schmitz MR, Boomsma S, et al. Consensus and Equipose in the Management of Military Trainee Femoral Neck Stress Fractures: A Survey of Military Surgeons. Mil Med. 2024;189:e82-e89.
4. Coombs CV, O'Leary TJ, Tang JC, Fraser WD, Greeves JP. Hormonal contraceptive use, bone density and biochemical markers of bone metabolism in British Army recruits. BMJ Mil Health. 2023; 169:9-16.
5. Fagnant HS, Lutz LJ, Nakayama AT, Gaffney-Stomberg E, McClung JP, Karl JP. Breakfast Skipping Is Associated with Vitamin D Deficiency among Young Adults entering Initial Military Training. J Acad Nutr Diet. 2022;122:1114-1128.e1.
6. Gaffney-Stomberg E, Hughes JM, Guerriere KI, Staab JS, Cable SJ, Bouxsein ML, et al., Once daily calcium (1000 mg) and vitamin D (1000 IU) supplementation during military training prevents increases in biochemical markers of bone resorption but does not affect tibial microarchitecture in Army recruits. Bone. 2022;155:116269.

7. Gill N, O'Leary T, Roberts A, Liu A, Roerdink M, Greeves J, et al. Enforcing walking speed and step-length affects joint kinematics and kinetics in male and female healthy adults. Gait Posture. 2023;103:223-228.
8. Hennigar SR, Kelley AM, Nakayama AT, Anderson BJ, McClung JP, Gaffney-Stomberg E. Divergent effects of sex and calcium/vitamin D supplementation on serum magnesium and markers of bone structure and function during initial military training. Br J Nutr. 2022;128: 1730-1737.
9. Leggit JC, Wu H, Janvrin M, Korona-Bailey J, Koehlmoos TP, Schneider EB. Non-Operative Shoulder Dysfunction in the United States Military. Mil Med. 2023;188:e1003-e1009.
10. Milgrom C, Tsur N, Eshed I, Milgrom Y, Beyth S, Spitzer E, et al. Significance of tibial MRI findings of special forces recruits at the onset of their training. BMJ Mil Health. 2024; 170:9-14.
11. O'Leary TJ, Coombs CV, Edwards VC, Blacker SD, Knight RL, Koivula FN, et al. The effect of sex and protein supplementation on bone metabolism during a 36-h military field exercise in energy deficit. J Appl Physiol. 2023;134: 1481-1495.
12. Pietsch H, Danelson K, Cavanaugh J, Hardy W. A comparison of fracture response in female and male lumbar spine in simulated under body blast component tests. J Mech Behav Biomed Mater. 2024;150:106303.
13. Rubio JE, Tong J, Sundaramurthy A, Subramani AV, Kote VB, Baggaley M, et al. Differences in running biomechanics between young, healthy men and women carrying external loads. Front Bioeng Biotechnol. 2023;11:1250937.
14. Shaw KA, Hattaway J, Villani N, Barkley C, O'Brien F, Jackson KL, et al. Surgically Treated Femoral Neck Stress Fractures Are Likely to Result in Military Separation During Basic Combat Training. Clin Orthop Relat Res. 2022;480:1684-1691.
15. Steinmann N, de Lange JE, Binette JS, Quenneville CE. Quantification of Behind Shield Blunt Impacts Using a Modified Upper Extremity Anthropomorphic Test Device. J Biomech Eng. 2022;144:091010.
16. Sundaramurthy A, Tong J, Subramani AV, Kote V, Baggaley M, Edwards WB et al. Effect of stride length on the running biomechanics of healthy women of different statures. BMC Musculoskelet Disord. 2023;24:604.
17. Taddei L, Bracq A, Delille R, Bourel B, Marechal C, Lauro F, et al. Effect of blast loading on the risk of rib fractures: a preliminary 3D numerical investigation. Forensic Sci Int. 2021;326: 110930.
18. Talbot M, Gear M, Young J, Milner D, Bunting A, Bozzo A. Risk assessment of aviators with a total hip arthroplasty. BMJ Mil Health. 2025;171;86-87.
19. Thelen M, Sutlive T, Tragord B, Robbins D, Schiferl R, Brock B, et al. Demonstration and Evaluation of Physical Examination Techniques Intended to Identify Proximal Femoral Bone Stress Injuries. Med J. 2022:66-73.
20. Tong J, Subramani AV, Kote V, Baggaley M, Edwards WB, Reifman J. Effects of Stature and Load Carriage on the Running Biomechanics of Healthy Men. IEEE Trans Biomed Eng. 2023. 70:2445-2453.
21. Beltran MJ, Burns TC, Eckel TT, Potter BK, Wenke JC, Hsu JR. Fate of combat nerve injury. J Orthop Trauma. 2012;26: e198-203.
22. Brown KV, Ramasamy A, Tai N, MacLeod J, Midwinter M, Clasper JC. Complications of extremity vascular injuries in conflict. J Trauma Acute Care Surg. 2009;66:S145-9.
23. Burns TC, Stinner DJ, Mack AW, Potter BK, Beer R, Eckel TT, et al. Microbiology and injury characteristics in severe open tibia fractures from combat. J Trauma Acute Care Surg. 2012; 72:1062-7.
24. Caubere A, Demoures T, Choufani C, Huynh V, Barbier O. Use of intramedullary nailing in poor sanitary conditions: French Military Medical Service experience. Orthop Traumatol Surg Res. 2019;105:173-177.
25. Chalupa RL, Rivera JC, Tennent DJ, Johnson AE. Correlation Between Femoral Neck Shaft Angle and Surgical Management in Trainees With Femoral Neck Stress Fractures. US Army Med Dep J. 2016:1-5.
26. Xu C, Silder A, Zhang J, Reifman J, Unnikrishnan G. A cross-sectional study of the effects of load carriage on running characteristics and tibial mechanical stress: implications for stress-fracture injuries in women. BMC Musculoskelet Disord. 2017;18:1-12.
27. Webster CE, Clasper J, Stinner DJ, Eliahoo J, Masouros SD. Characterization of Lower Extremity Blast Injury. Mil Med. 2018;**183**:e448-e453.
28. Cross JD, Stinner DJ, Burns TC, Wenke JC, Hsu JR, Skeletal Trauma Research Consortium. Return to duty after type III open tibia fracture. J Orthop Trauma. 2012;26:43-7.
29. Danelson K, Watkins L, Hendricks J, Frounfelker P, Pizzolato-Heine K, Valentine R. Analysis of the Frequency and Mechanism of Injury to Warfighters in the Under-body Blast Environment. SAE Int. 2018;62:489-513.
30. De Kruijff LG, Prins M, Van Der Krans A, Hoencamp R, Van Der Wurff P. Combat-related foot injuries: impact on gait and functional outcome. BMJ Mil Health. 2018;164:322-327.

31. Dunn JC, Fares A, Kusnezov N, Chandler P, Cordova C, Orr J, et al. US service member tourniquet use on the battlefield: Iraq and Afghanistan 2003–2011. Trauma. 2016;18:216-220.
32. Dutton J, Bromhead SE, Speed CA, Menzies AR, Peters AM. Clinical value of grading the scintigraphic appearances of tibial stress fractures in military recruits. Clin Nucl Med. 2002; 27:18-21.
33. Earl-Boehm JE, Poel DN, Zalewski K, Ebersole KT. The effects of military style ruck marching on lower extremity loading and muscular, physiological and perceived exertion in ROTC cadets. Ergonomics, 2020;63:629-638.
34. Fox CJ, Gillespie DL, O'Donnell SD, Rasmussen TE, Goff JM, Johnson CA, et al. Contemporary management of wartime vascular trauma. J Vasc Surg, 2005;41:638-44.
35. Gaffney-Stomberg E, Lutz LJ, Rood JC, Cable SJ, Pasiakos SM, Young AJ, et al. Calcium and vitamin D supplementation maintains parathyroid hormone and improves bone density during initial military training: a randomized, double-blind, placebo controlled trial. Bone. 2014;68:46-56.
36. Gaffney-Stomberg E, Lutz LJ, Shcherbina A, Ricke DO, Petrovick M, Cropper TL, et al. Association Between Single Gene Polymorphisms and Bone Biomarkers and Response to Calcium and Vitamin D Supplementation in Young Adults Undergoing Military Training. J Bone Miner Res. 2017;32:498-507.
37. Havenetidis K, Kardaris D, Paxinos T. Profiles of musculoskeletal injuries among Greek Army officer cadets during basic combat training. Mil Med. 2011;176:297-303.
38. Hoyt BW, Lundy AE, Purcell RL, Harrington CJ, Gordon WT. Definitive External Fixation for Anterior Stabilization of Combat-related Pelvic Ring Injuries, With or Without Sacroiliac Fixation. Clin Orthop Relat Res. 2020;478:779-789.
39. Lake N, Mombell KW, Bernstein E, O'Mary K, Scott J, Deafenbaugh B. Improved Functional Outcomes Following Operative Treatment of Midshaft Clavicle Fractures in an Active Duty Population. Cureus. 2020;12.
40. Lee C, Feaker DA, Ostrofe AA, Smith CS. No Difference in Risk of Implant Removal Between Orthogonal Mini-fragment and Single Small-fragment Plating of Midshaft Clavicle Fractures in a Military Population: A Preliminary Study. Clin Orthop Relat Res. 2020;478:741-749.
41. Leisey J. Prospective validation of the Ottawa Ankle Rules in a deployed military population. Mil Med, 2004;169:804-6.
42. Lewandowski LR, Potter BK, Murray CK, Petfield J, Stinner DJ, Krauss M, et al. Osteomyelitis Risk Factors Related to Combat Trauma Open Femur Fractures: A Case-Control Analysis. J Orthop Trauma. 2019;33:e110-e119.
43. De Kruijff LG, Prins M, Van Der Krans A, Hoencamp R, Van Der Wurff P. Combat-related foot injuries: impact on gait and functional outcome. J R Army Med Corps, 2018. **164**(5): p. 322.
44. Lovalekar M, Abt JP, Sell TC, Lephart SM, Pletcher E, Beals K. Accuracy of recall of musculoskeletal injuries in elite military personnel: a cross-sectional study. BMJ Open. 2017;7:e017434.
45. Mabry RL, Edens JW, Pearse L, Kelly JF, Harke H. Fatal airway injuries during Operation Enduring Freedom and Operation Iraqi Freedom. Prehosp Emerg Care. 2010;14:272-7.
46. May T, Marra J, Leu A, Torbert D, VanWagner T, Alexander Z, et al. Accuracy of the Tuning Fork Test for Determination of Presence and Location of Tibial Stress Fractures in a Military Training Population. Mil Med. 2021;186:733-736.
47. McKay BJ, Bir CA. Lower extremity injury criteria for evaluating military vehicle occupant injury in underbelly blast events. SAE Int. 2009;53:229-49.
48. Milgrom C, Zloczower E, Fleischmann C, Spitzer E, Landau R, Bader T, et al. Medial tibial stress fracture diagnosis and treatment guidelines. J Sci Med Sport. 2021;24:526-530.
49. Motamedi MH, Khatami SM, Tarighi P. Assessment of severity, causes, and outcomes of hospitalized trauma patients at a major trauma center. J Trauma Acute Care Surg. 2009;66:516-8.
50. Nappo KE, Hoyt BW, Balazs GC, Nanos GP, Ipsen DF, Tintle SM, et al. Union Rates and Reported Range of Motion Are Acceptable After Open Forearm Fractures in Military Combatants. Clin Orthop Relat Res. 2019;477:813-820.
51. Nunns M, Stiles V, Fulford J, Dixon S. Estimated third metatarsal bending stresses are highly susceptible to variations in bone geometry. Footwear Science. 2017;9:127-137.
52. Orr JD, Kusnezov NA, Waterman BR, Bader JO, Romano DM, Belmont Jr PJ. Occupational Outcomes and Return to Running Following Internal Fixation of Ankle Fractures in a High-Demand Population. Foot & Ankle International. 2015;36:780-786.
53. Pehlivan O, Kiral A, Akmaz I, Solakoglu C, Arpacioglu O, Kaplan H. Humeral shaft fractures secondary to throwing. Orthopedics. 2003. 26:1139-41.
54. Petfield JL, Hayeck GT, Kopperdahl DL, Nesti LJ, Keaveny TM, Hsu JR, et al. Virtual stress testing of fracture stability in soldiers with severely comminuted tibial fractures. J Orthop Res. 2017;35:805-811.
55. Plavina L. *Evaluation of Stress Fracture Risk Factors for Recruits.* Papers on Anthropology. 2004;13:185-192.

56. Ramasamy A, Hill AM, Phillip R, Gibb I, Bull AM, Clasper JC. The modern "deck-slap" injury--calcaneal blast fractures from vehicle explosions. J Trauma Acute Care Surg. 2011;**71**:1694-8.
57. Rice H, Fallowfield J, Allsopp A, Dixon S. Altered forefoot function following a military training activity. Gait Posture. 2019;74:182-186.
58. Rice HM, Saunders SC, McGuire SJ, O'Leary TJ, Izard RM. Estimates of Tibial Shock Magnitude in Men and Women at the Start and End of a Military Drill Training Program. Mil Med. 2018;183:e392-e398.
59. Richards T, Wright C. British Army recruits with low serum vitamin D take longer to recover from stress fractures. BMJ Mil Health. 2020;166:240-242.
60. Richardson PS. Dental morbidity in United Kingdom Armed Forces, Iraq 2003. Mil Med. 2005; 170:536-41.
61. Schechtman DW, Walters TJ, Kauvar DS. Utility of the Mangled Extremity Severity Score in Predicting Amputation in Military Lower Extremity Arterial Injury. Ann Vasc Surg. 2021;70:95-100.
62. Stewart SK, Pearce AP, Clasper JC. Clasper, Fatal head and neck injuries in military underbody blast casualties. BMJ Mil Health. 2019;165:18-21.
63. Xu C, Silder A, Zhang J, Reifman J, Unnikrishnan G. A cross-sectional study of the effects of load carriage on running characteristics and tibial mechanical stress: implications for stress-fracture injuries in women. BMC Musculoskelet Disord. 2017;18:125.
64. Gaffney-Stomberg E, Nakayama AT, Guerriere KI, Lutz LJ, Walker LA, Staab JS. Calcium and vitamin D supplementation and bone health in Marine recruits: Effect of season. Bone. 2019; 123:224-233.
65. Barnes KR, Tchandja JN, Webber BJ, Federinko SP, Cropper TL. The effects of prenatal vitamin supplementation on operationally significant health outcomes in female air force trainees. Mil Med. 2015;180:554-8.
66. Almeida SA, Williams KM, Shaffer RA, Brodine SK. Epidemiological patterns of musculoskeletal injuries and physical training. Med Sci Sports Exerc. 1999;31:1176-82.
67. Amoroso PJ, Bell NS, Jones BH. Injury among female and male army parachutists. Aviat Space Environ Med. 1997;68:1006-11.
68. Bar-Dayan Y, Bar-Dayan Y, Shemer J. Parachuting injuries: a retrospective study of 43,542 military jumps. Mil Med. 1998;163:1-2.
69. Bennell KL, Brukner PD. Epidemiology and site specificity of stress fractures. Clin Sports Med. 1997;16:179-96.
70. Bijur PE, Horodyski M, Egerton W, Kurzon M, Lifrak S, Friedman S. Comparison of injury during cadet basic training by gender. Arch Pediatr Adolesc Med. 1997;151:456-61.
71. Cernak I, Savic J, Zunic G, Pejnovic N, Jovanikic O, Stepic V. Recognizing, scoring, and predicting blast injuries. World J Surg. 1999;23:44-53.
72. Cline AD, Jansen GR, Melby CL. Stress fractures in female army recruits: implications of bone density, calcium intake, and exercise. J Am Coll Nutr. 1998;17:128-35.
73. Cowan DN, Jones BH, Frykman PN, Polly Jr DW, Harman EA, Rosenstein RM, et al. Lower limb morphology and risk of overuse injury among male infantry trainees. Med Sci Sports Exerc. 1996;28:945-52.
74. Craig SC, Zugner D, Knapik JJ, Bricknell MC. Parachuting injuries during Operation Royal Dragon, Big Drop III, Fort Bragg, North Carolina, May 15/16, 1996. Mil Med. 1999;164:41-3.
75. Ekeland A. Injuries in military parachuting: a prospective study of 4499 jumps. Injury.1997;28:219-22.
76. Farrow GB. Military static line parachute injuries. Aust N Z J Surg. 1992;62:209-14.
77. Finestone A, Giladi M, Elad H, Salmon A, Mendelson S, Eldad A, et al. Prevention of stress fractures using custom biomechanical shoe orthoses. Clin Orthop Rel Res. 1999;360:182-190.
78. Finestone A, Shlamkovitch N, Eldad A, Wosk J, Laor A, Danon YL. Risk factors for stress fractures among Israeli infantry recruits. Mil Med. 1991;156:528-30.
79. Friberg O. Leg length asymmetry in stress fractures: a clinical and radiological study. J Sports Med Phys Fit. 1982;22:485-488.
80. Friedl KE, Nuovo JA, Patience TH, Dettori JR. Factors associated with stress fracture in young army women: indications for further research. Mil Med. 1992;157:334-8.
81. Gant TD, Epstein LI. Low-velocity gunshot wounds to the maxillofacial complex. J Trauma Acute Care Surg. 1979;19:674-7.
82. Garcia JE, Grabhorn LL, Franklin KJ. Factors associated with stress fractures in military recruits. Mil Med. 1987;152:45-8.
83. Giladi M, Milgrom C, Kashtan H, Stein M, Chisin R, Dizian R. Recurrent stress fractures in military recruits. One-year follow-up of 66 recruits. J Bone Joint Surg Br. 1986;68:439-41.
84. Giladi M, Milgrom C, Simkin A, Danon Y. Stress fractures. Identifiable risk factors. Am J Sports Med. 1991;19:647-52.

85. Giladi M, Milgrom C, Stein M, Kashtan H, Margulies J, Chisin R, et al. External rotation of the hip. A predictor of risk for stress fractures. Clin Orthop Relat Res. 1987;216:131-4.
86. Gill RM, Hopkins GO. Stress fracture in parachute regiment recruits. J R Army Med Corps. 1988;134:91-3.
87. Gordon NF, Hugo EP, Cilliers J. The South African Defence Force physical training programme. Part III. Exertion-related injuries sustained at an SADF basic training centre. S Afr Med J. 1986;69:491-494.
88. Gardner Jr LI, Dziados JE, Jones BH, Brundage JF, Harris JM, Sullivan R, et al. Prevention of Lower Extremity Stress Fractures: A Controlled Trial of a Shock Absorbent Insole. Am J Public Health. 1988;78:1563-1567.
89. Greaney RB, Gerber FH, Laughlin RL, Kmet JP, Metz CD, Kilcheski TS, et al. Distribution and natural history of stress fractures in U.S. Marine recruits. Radiol. 1983;146:339-46.
90. Grimston SK, Zernicke RF. Exercise-Related Stress Responses in Bone. J of Appl Biomech. 1993;9:2-14.
91. Hansen MO, Polly DW, McHale KA, Asplund LM. A prospective evaluation of orthopedic patients evacuated from Operations Desert Shield and Desert Storm: the Walter Reed experience. Mil Med. 1994;159:376-80.
92. Hill PF, Chatterji S, Chambers D, Keeling JD. Stress fracture of the pubic ramus in female recruits. J Bone Joint Surg Br. 1996;78:383-6.
93. Hodalić Ž, Švagelj M, Šebalj I, Šebalj Đ. Surgical treatment of 1,211 patients at the Vinkovci General Hospital, Vinkovci, Croatia, during the 1991-1992 Serbian offensive in east Slavonia. Mil Med. 1999;164:803-8.
94. Hoffman JR, Chapnik L, Shamis A, Givon U, Davidson B. The effect of leg strength on the incidence of lower extremity overuse injuries during military training. Mil Med. 1999;164:153-6.
95. Hopson CN, Perry DR. Stress fractures of the calcaneus in women marine recruits. Clin Orthop Relat Res. 1977;128:159-62.
96. Islinger RB, Kuklo TR, Polly Jr DW. Spine fractures in active duty soldiers and their return to duty rate. Mil Med. 1998;163:536-9.
97. Ivanovic A, Jovic N, Vukelic-Markovic S. Frontoethmoidal fractures as a result of war injuries. J Trauma Acute Care Surg. 1996;40:S177-9.
98. Jacob E, Erpelding JM, Murphy KP. A retrospective analysis of open fractures sustained by U.S. military personnel during Operation Just Cause. Mil Med. 1992;157:552-6.
99. Johnson BA, Neylon T, Laroche R. Lesser metatarsal stress fractures. Clin Podiatr Med Surg. 1999;16:631-642.
100. Jones BH, Knapik JJ. Physical training and exercise-related injuries. Surveillance, research and injury prevention in military populations. Sports Med. 1999;27:111-25.
101. Jordaan G, Schwellnus MP. The incidence of overuse injuries in military recruits during basic military training. Mil Med. 1994;159:421-6.
102. Jovanovic S, Wertheimer B, Zelic Z, Getos Z. Wartime amputations. Mil Med. 1999;164:44-7.
103. Kazarian LE. Identification and classification of vertebral fractures following emergency capsule egress from military aircraft. Aviat Space Environ Med. 1978;49:150-7.
104. Korzinek K. War injuries of the extremities. Der Unfallchirurg. 1993;96:242-7.
105. Kuusela TV. Incidence of bone lesions in the lower extremities during endurance training. Ann Clin Res. 1984;16:17-19.
106. Lacombe J, Beauche A, Tripon P, Salasc P, Ferret JN, Esling F. Epidemiologie des accidents traumatiques en aile planante en milieu militaire. / Epidemiology of taumatic accidents in military hang-gliding. J de Traumatol du Sport. 1993;10:170-174.
107. Leedham CS, Blood CG, Newland C. A descriptive analysis of wounds among U.S. Marines treated at second-echelon facilities in the Kuwaiti theater of operations. Mil Med. 1993;158:508-12.
108. Lesho EP. Can tuning forks replace bone scans for identification of tibial stress fractures? Mil Med. 1997;162:802-3.
109. Levi L, Borovich B, Guilburd JN, Grushkiewicz I, Lemberger A, Linn S, et al. Wartime neurosurgical experience in Lebanon, 1982-85. II: Closed craniocerebral injuries. Isr J Med Sci. 1990;26:555-8.
110. Linenger JM, Shwayhat AF. Epidemiology of podiatric injuries in US Marine recruits undergoing basic training. J Am Podiatr Med Assoc. 1992;82:269-71.
111. Lowdon IM, Wetherill MH. Parachuting injuries during training descents. Injury. 1989;20:257-8.
112. Macleod MA, Houston AS, Sanders L, Anagnostopoulos C. Incidence of trauma related stress fractures and shin splints in male and female army recruits: retrospective case study. Bmj. 1999;318:29.
113. McCarroll JE, Gunderson C. 5-year study of incidence rates of hospitalized cases of head injuries in the US Army. Neuroepidemiology. 1990;9:296-305.

114. Meurman KO. Stress fracture of the pubic arch in military recruits. Br J Radiol. 1980;53:521-4.
115. Milgrom, C., Finestone, A., Mendelson, S. Mendel, D. Edad, H. Nyska, M. Benjoya, N. Simkin, A. Chankov, C. Voloshin, A. Eldad, A., 'The effect of pre-induction sports participation on the incidence of stress fractures in Israeli infantry recruits', Israel; 1998.
116. Milgrom C, Giladi M, Kashtan H, Simkin A, Chisin R, Margulies J, et al. A prospective study of the effect of a shock-absorbing orthotic device on the incidence of stress fractures in military recruits. Foot Ankle. 1985;6:101-4.
117. Milgrom C, Giladi M, Simkin A, Rand N, Kedem R, Kashtan H, et al. An analysis of the biomechanical mechanism of tibial stress fractures among Israeli infantry recruits. A prospective study. Clin Orthop Relat Res. 1988;231:216-21.
118. Milgrom C, Giladi M, Stein M, Kashtan H, Margulies J, Chisin R, et al. Medial tibial pain. A prospective study of its cause among military recruits. Clin Orthop Relat Res. 1986;213:167-71.
119. Milgrom C, Giladi M, Stein M, Kashtan H, Margulies JY, Chisin R, et al. Stress fractures in military recruits. A prospective study showing an unusually high incidence. J Bone Joint Surg Br. 1985;67:732-5.
120. Montgomery LC, Nelson FR, Norton JP, Deuster PA. Orthopedic history and examination in the etiology of overuse injuries. Med Sci Sports Exerc. 1989;21:237-43.
121. Murray-Leslie CF, Lintott DJ, Wright V. The knees and ankles in sport and veteran military parachutists. Ann Rheum Dis. 1977;36:327-31.
122. Murray-Leslie CF, Lintott DJ, Wright V. The spine in sport and veteran military parachutists. Ann Rheum Dis. 1977;36:332-42.
123. Newman DG. The ejection experience of the Royal Australian Air Force: 1951-92. Aviat Space Environ Med. 1995;66:45-9.
124. Novak WA. Use of Ottawa ankle decision rules to evaluate blunt ankle trauma case studies by United States Air Force health care providers. Uniformed Services University of the Health Sciences. 1999:82.
125. Osborne RG, Cook AA. Vertebral fracture after aircraft ejection during Operation Desert Storm. Aviat Space Environ Med. 1997;68:337-41.
126. Parsons III TW, Lauerman WC, Ethier DB, Gormley W, Cain JE, Elias Z, et al. Spine injuries in combat troops--Panama, 1989. Mil Med. 1993;158:501-2.
127. Pester S, Smith PC. Stress fractures in the lower extremities of soldiers in basic training. Orthop Rev. 1992;21:297-303.
128. Pope RP. Prevention of pelvic stress fractures in female army recruits. Mil Med. 1999;164:370-3.
129. Pope RP, Herbert R, Kirwan JD, Graham BJ.Predicting attrition in basic military training. Mil Med. 1999;164:710-4.
130. Pouilles JM, Bernard J, Tremollieres F, Louvet JP, Ribot C. Femoral bone density in young male adults with stress fractures. Bone. 1989;10:105-8.
131. Protzman RR, Colonel L, Corps AM. Physiologic performance of women compared to men. Observations of cadets at the United States Military Academy. Am J Sports Med. 1979;7:191-4.
132. Reynolds KL, Heckel HA, Witt CE, Martin JW, Pollard JA, Knapik JJ, et al. Cigarette smoking, physical fitness, and injuries in infantry soldiers. Am J Prev Med. 1994;10:145-50.
133. Rodden JW, Simecek JW. Dental emergency visits of Marine Corps personnel. Mil Med. 1995;160:555-7.
134. Rosin A, Sinopoli M. Impact of the Ottawa Ankle Rules in a U.S. Army troop medical clinic in South Korea. Military Medicine. 1999;164:793-4.
135. Ross J. A review of lower limb overuse injuries during basic military training. Part 1: Types of overuse injuries. Mil Med. 1993;158:410-5.
136. Rudzki SJ. Injuries in Australian Army recruits. Part II: Location and cause of injuries seen in recruits. Mil Med. 1997;162:477-80.
137. Sahi T, Friberg O, Riihimäki M, Tikkinen J. Epidemiology, etiology and prevention of stress fractures in the Finnish defense forces and the frontier guard. InSports Injuries: Proceedings of the 3rd Jerusalem Symposium. Freund Publishing House. 1988:113-126.
138. Schissel DJ, Godwin J. Effort-related chronic compartment syndrome of the lower extremity. Mil Med. 1999;164:830-2.
139. Schwellnus MP, Jordaan G, Noakes TD. Prevention of common overuse injuries by the use of shock absorbing insoles. A prospective study. Am J Sports Med. 1990;18:636-41.
140. Shaffer RA, Brodine SK, Almeida SA, Williams KM, Ronaghy S. Use of simple measures of physical activity to predict stress fractures in young men undergoing a rigorous physical training program. Am J Epidemiol. 1999;149:236-42.
141. Shanahan DF. Basilar skull fracture in U.S. Army aircraft accidents. Aviat Space Environ Med. 1983;54:628-31.

142. Shappell SA. Naval flight deck injuries: a review of Naval Safety Center data, 1977-91. Aviat Space Environ Med. 1995;66:590-5.
143. Simchen E, Raz R, Stein H, Danon Y. Risk factors for infection in fracture war wounds (1973 and 1982 wars, Israel). Mil Med. 1991;156:520-7.
144. Simkin A, Leichter I, Giladi M, Stein M, Milgrom C. Combined effect of foot arch structure and an orthotic device on stress fractures. Foot Ankle Int. 1989;10:25-29.
145. Stoneham MD, Morgan NV. Stress fractures of the hip in Royal Marine recruits under training: a retrospective analysis. Br J Sports Med. 1991;25:145-8.
146. Swissa A, Milgrom C, Giladi M, Kashtan H, Stein M, Margulies J, et al. The effect of pretraining sports activity on the incidence of stress fractures among military recruits. A prospective study. Clin Orthop Relat Res. 1989;245:256-60.
147. Taimela S, Kujala UM, Österman K. Stress injury proneness: a prospective study during a physical training program. Int J Sports Med. 1990;11:162-5.
148. Truax AL, Chandnani VP, Chacko AK, Gonzalez DM. Incidence and methods of diagnosis of musculoskeletal injuries incurred in Operations Desert Shield and Desert Storm. Invest Radiol. 1997;32:169-73.
149. Werner U. Ejection associated injuries within the German Air Force from 1981-1997. Aviat Space Environ Med. 1999;70:1230-4.
150. Winfield AC, Moore J, Bracker M, Johnson CW. Risk factors associated with stress reactions in female Marines. Mil Med. 1997;162:698-702.
151. Alhawas A, Abahussain M, Alghamdi SG, Alfarhan A, Alhawas Sr AM. Completely Displaced Femoral Neck Stress Fracture in a Young Male Soldier With Almost No Functional Impact: A Case Report. Cureus. 2023;15:e33629.
152. Zalneraitis BH, Huuki E, Benavides LC, Benavides JM. Relation of Vitamin D Level, BMI, and Location of Lower Extremity Stress Fractures in Military Trainees. Mil Med. 2022.
153. Tu MY, Chu H, Huang YJ, Chiang KT, Tong PT, Lai CY. A Rare Case of Rib Fractures During Centrifuge Training. Mil Med. 2022;187:e242-e245.
154. Parker W, Despain RW, Bailey J, Elster E, Rodriguez CJ, Bradley M. Military experience in the management of pelvic fractures from OIF/OEF. BMJ Mil Health. 2023;169:108-111.
155. Petfield JL, Lewandowski LR, Stewart L, Murray CK, Tribble DR. IDCRP Combat-Related Extremity Wound Infection Research. Mil Med. 2022;187:25-33.
156. Ran Y, Mitchnik I, Gendler S, Avital G, Radomislensky I, Bodas M, et al. Isolated limb fractures - the underestimated injury in the Israeli Defence Forces (IDF). Injury. 2023;54:490-496.
157. Schulte SS, Fares AB, Childs BR, Kenney LE, Orr JD. Factors associated with return to duty and need for subsequent procedures after calcaneus open reduction internal fixation in the military. Injury. 2022;53:771-776.
158. Sommer F, Gadjradj PS, Pippig T. Spinal injuries after ejection seat evacuation in fighter aircraft of the German Armed Forces between 1975 and 2021. J Neurosurg Spine. 2023;38:271-278.
159. Tsur N, Talmy T, Radomislensky I, Almog O, Gendler S. Traumatic maxillofacial injuries: Patterns, outcomes, and long-term follow-up of a military cohort. Dent Traumatol. 2023;39:147-156.
160. Yang JZ, Chen P, Chen BH, Zhao B. Subchondral fatigue fracture of the femoral head in young military recruits: Potential risk factors. World J Clin Cases. 2023;11:6733-6743.
161. Bandyopadhyay K, Ray S, Shikha D, Bhalla GS, Khetan A. Risk factors of osteoporosis in soldiers of the Armed Forces: A cross-sectional study from Western India. Med J Armed Forces India. 2023;79:194-200.
162. Gardner CL, Raps SJ, Bedford T, Fisher RA. A Case-Control Analysis of Bone Stress Injury on Advancement and Health Care Utilization in US Air Force Basic Military Trainees. Mil Med. 2023;188:690-697.
163. Nesterovica D, Vaivads N, Stepens A. Relationship of footwear comfort, selected size, and lower leg overuse injuries among infantry soldiers. BMC Musculoskelet Disord. 2021;22:952.
164. Peterson SL, Kingsbury TD, Djafar T, Stewart J, Kuhn KM. Military Service Members with Major Lower Extremity Fractures Return to Running with a Passive-dynamic Ankle-foot Orthosis: Comparison with a Normative Population. Clin Orthop Relat Res. 2021;479:2375-2384.
165. Zafar SI, bin Saeed H, Asif M, Anwer A, Syed HM. Patterns of Musculoskeletal Injuries Secondary to Strenuous Physical Training, Assessed on Magnetic Resonance Imaging. Pak Armed Forces Med J. 2024;74:282.
166. Armstrong III DW, Rue JP, Wilckens JH, Frassica FJ. Stress fracture injury in young military men and women. Bone. 2004;35:806-16.
167. Beck TJ, Ruff CB, Shaffer RA, Betsinger K, Trone DW, Brodine SK. Stress fracture in military recruits: gender differences in muscle and bone susceptibility factors. Bone. 2000;27:437-44.

168. Burgi AA, Gorham ED, Garland CF, Mohr SB, Garland FC, Zeng K, et al. High serum 25-hydroxyvitamin D is associated with a low incidence of stress fractures. J Bone Miner Res. 2011;26:2371-7.
169. Chatzipapas CN, Drosos GI, Kazakos KI, Tripsianis G, Iatrou C, Verettas DA. Stress Fractures in Military. Men and Bone duality Related Factors. Int J Sports Med. 2008;29:922-926.
170. Davey T, Lanham-New SA, Shaw AM, Cobley R, Allsopp AJ, Hajjawi MO, et al. Fundamental differences in axial and appendicular bone density in stress fractured and uninjured Royal Marine recruits--a matched case-control study. Bone. 2015;73:120-6.
171. Dixon SJ, Creaby MW, Allsopp AJ. Comparison of static and dynamic biomechanical measures in military recruits with and without a history of third metatarsal stress fracture. Clin Biomech. 2006;21:412-9.
172. Finestone A, Milgrom C, Evans R, Yanovich RA, Constantini NA, Moran DS. Overuse injuries in female infantry recruits during low-intensity basic training. Med Sci Sports Exerc. 2008;40:S630-5.
173. Givon U, Friedman E, Reiner A, Vered I, Finestone A, Shemer J. Stress fractures in the Israeli defense forces from 1995 to 1996. Clin Orthop Relat Res. 2000:227-32.
174. Kelly EW, Jonson SR, Cohen ME, Shaffer R. Stress fractures of the pelvis in female Navy recruits: An analysis of possible mechanisms of injury. Mil Med. 2000;165:142-6.
175. Korvala J, Hartikka H, Pihlajamäki H, Solovieva S, Ruohola JP, Sahi T, et al. Genetic predisposition for femoral neck stress fractures in military conscripts. BMC Genetics. 2010;11:95.
176. Kuhn KM, Riccio AI, Saldua NS, Cassidy J. Acetabular retroversion in military recruits with femoral neck stress fractures. Clin Orthop Relat Res. 2010;468:846-51.
177. Kupferer KR, Bush DM, Cornell JE, Lawrence VA, Alexander JL, Ramos RG, et al. Femoral Neck Stress Fracture in Air Force Basic Trainees. Mil Med. 2014;179:56-61.
178. Lauder TD, Dixit S, Pezzin LE, Williams MV, Campbell CS, Davis GD. The relation between stress fractures and bone mineral density: evidence from active-duty Army women. Arch Phys Med Rehabil. 2000;81:73-9.
179. Nunns M, House C, Rice H, Mostazir M, Davey T, Stiles V, et al. Four biomechanical and anthropometric measures predict tibial stress fracture: a prospective study of 1065 Royal Marines. Br J Sports Med. 2016;50:1206-10.
180. Schwartz O, Bulis S, Olsen CH, Glasberg E, Dudkiewicz I. The Association Between History of an Ankle Sprain and Traumatic Meniscal Injury Among Infantry Combat Soldiers in the Israeli Defense Forces: A Historical Cohort Study. Mil Med. 2020;185:e748-e754.
181. Sanchez-Santos MT, Davey T, Leyland KM, Allsopp AJ, Lanham-New SA, Judge A, et al. Development of a Prediction Model for Stress Fracture During an Intensive Physical Training Program: The Royal Marines Commandos. Orthop J Sports Med. 2017;5:1-12.
182. Scheinowitz M, Yanovich R, Sharvit N, Arnon M, Moran DS. Effect of cardiovascular and muscular endurance is not associated with stress fracture incidence in female military recruits: a 12-month follow up study. J Basic Clin Physiol Pharmacol. 2017;28:219-224.
183. Schermann H, Ben-Ami IS, Tudor A, Amar E, Rath E, Yanovich R. Past Methylphenidate Exposure and Stress Fractures in Combat Soldiers: A Case-Control Study. Am J Sports Med. 2018;46:728-733.
184. Strohbach CA, Scofield DE, Nindl BC, Centi AJ, Yanovich R, Evans RK, et al. Female recruits sustaining stress fractures during military basic training demonstrate differential concentrations of circulating IGF-I system components: a preliminary study. Growth Horm IGF Res. 2012;22:151-7.
185. Yanovich R, Evans RK, Friedman E, Moran DS. Bone turnover markers do not predict stress fracture in elite combat recruits. Clin Orthop Relat Res. 2013;471:1365-72.
186. Yanovich R, Friedman E, Milgrom R, Oberman B, Freedman L, Moran DS. Candidate gene analysis in Israeli soldiers with stress fractures. J Sports Sci Med. 2012;11:147-155.
187. Lauder TD, Baker SP, Smith GS, Lincoln AE. Sports and physical training injury hospitalizations in the army. Am J Prev Med. 2000;18:118-28.
188. Amako M, Yato Y, Yoshihara Y, Arino H, Sasao H, Nemoto O, et al. Epidemiological patterns of traumatic musculoskeletal injuries and non-traumatic disorders in Japan Self-Defense Forces. Injury Epidemiol. 2018;5:1-1.
189. Bergman BP, St J Miller SA. Equal opportunities, equal risks? Overuse injuries in female military recruits. J Public Health Med. 2001;23:35-9.
190. Lovalekar M, Keenan KA, Chang YF, Wirt MD, Nindl BC, Beals K, et al. Using the capture-recapture method to estimate the incidence of musculoskeletal injuries among U.S. Army soldiers. J Sci Med Sport. 2017;20:S23-S27.
191. Lovalekar M, Perlsweig KA, Keenan KA, Baldwin TM, Caviston M, McCarthy AE, et al. Epidemiology of musculoskeletal injuries sustained by Naval Special Forces Operators and students. J Sci Med Sport. 2017;20:S51-s56.

192. Schwartz O, Malka I, Olsen CH, Dudkiewicz I, Bader T. Overuse Injuries in the IDF's Combat Training Units: Rates, Types, and Mechanisms of Injury. Mil Med. 2018;183:e196-e200.
193. Schwartz O, Malka I, Olsen CH, Dudkiewicz I, Bader T. Overuse Injuries Among Female Combat Warriors in the Israeli Defense Forces: A Cross-sectional Study. Mil Med. 2018;183:e610-e616.
194. Blair JA, Patzkowski JC, Schoenfeld AJ, Rivera JD, Grenier ES, Lehman Jr RA, et al. Spinal column injuries among Americans in the global war on terrorism. J Bone Joint Surg Am. 2012;94:e135.
195. Blair JA, Patzkowski JC, Schoenfeld AJ, Rivera JD, Grenier ES, Lehman RA, et al. Are spine injuries sustained in battle truly different? Spine J. 2012;12:824-9.
196. Blair JA, Possley DR, Petfield JL, Schoenfeld AJ, Lehman RA, Hsu JR, et al. Military penetrating spine injuries compared with blunt. Spine J. 2012;12:762-8.
197. Breeze J, Gibbons AJ, Hunt NC, Monaghan AM, Gibb I, Hepper A, et al. Mandibular fractures in British military personnel secondary to blast trauma sustained in Iraq and Afghanistan. Br J Oral Maxillofac Surg. 2011;49:607-11.
198. Breeze J, Gibbons AJ, Opie NJ, Monaghan A. Maxillofacial injuries in military personnel treated at the Royal Centre for Defence Medicine June 2001 to December 2007. Br J Oral Maxillofac Surg. 2010;48:613-6.
199. Burtis MT, Faillace J, Martin LF, Hermenau S. Scaphoid fracture detection in a military population: a standardized approach for medical referral. Mil Med. 2006;171:404-8.
200. Carey T, Key C, Oliver D, Biega T, Bojescul J. Prevalence of radiographic findings consistent with femoroacetabular impingement in military personnel with femoral neck stress fractures. J Surg Orthop Adv. 2013;22:54-8.
201. Comstock S, Pannell D, Talbot M, Compton L, Withers N, Tien HC. Spinal injuries after improvised explosive device incidents: implications for Tactical Combat Casualty Care. J Trauma. 2011;71:S413-7.
202. Doucet JJ, Galarneau MR, Potenza BM, Bansal V, Lee JG, Schwartz AK, et al. Combat versus civilian open tibia fractures: the effect of blast mechanism on limb salvage. J Trauma. 2011;70:1241-7.
203. Hauret KG, Taylor BJ, Clemmons NS, Block SR, Jones BH. Frequency and causes of nonbattle injuries air evacuated from operations iraqi freedom and enduring freedom, u.s. Army, 2001-2006. Am J Prev Med. 2010;38:S94-107.
204. Hauschild VD, Schuh A, Taylor BJ, Canham-Chervak M, Jones BH. Identification of specific activities associated with fall-related injuries, active component, U.S. Army, 2011. Msmr. 2016;23:2-9.
205. Hayton J.Reducing Medical Downgrading In A High Readiness Royal Marine Unit. J R Army Med Corps. 2004;150:164.
206. Jensen AE, Laird M, Jameson JT, Kelly KR. Prevalence of Musculoskeletal Injuries Sustained During Marine Corps Recruit Training. Mil Med. 2019;184:511-520.
207. Junge T, Bellamy J, Dowd T, Osborn P. Outcomes of Talus Fractures Associated With High-Energy Combat Trauma. Foot Ankle Int. 2017;38:1357-1361.
208. Kim KE, Kim EJ, Park J, Kim SW, Kwon J, Moon G. Humeral shaft fracture and radial nerve palsy in Korean soldiers: focus on arm wrestling related injury. BMJ Mil Health. 2021;167:80-83.
209. Lew TA, Walker JA, Wenke JC, Blackbourne LH, Hale RG. Characterization of craniomaxillofacial battle injuries sustained by United States service members in the current conflicts of Iraq and Afghanistan. J Oral Maxillofac Surg. 2010;68:3-7.
210. Lin DL, Kirk KL, Murphy KP, McHale KA, Doukas WC. Orthopedic injuries during Operation Enduring Freedom. Mil Med. 2004;169:807-9.
211. Madson AQ, Tucker D, Aden J, Hale RG, Chan RK. Non-battle craniomaxillofacial injuries from U.S. military operations. J Craniomaxillofac Surg. 2013;41:816-20.
212. Mattila VM, Parkkari J, Korpela H, Pihlajamäki H. Hospitalisation for injuries among Finnish conscripts in 1990-1999. Accid Anal Prev. 2006;38:99-104.
213. Mitchener TA, Hauret KG. Air medical evacuations of soldiers for oral-facial disease and injuries, 2005, Operations Enduring Freedom/Iraqi Freedom. Mil Med. 2009;174:376-81.
214. Motamedi MH, Sagafinia M, Famouri-Hosseinizadeh M. Oral and maxillofacial injuries in civilians during training at military garrisons: prevalence and causes. Oral Surg Oral Med Oral Pathol Oral Radiol. 2012;114:49-51.
215. Possley DR, Blair JA, Freedman BA, Schoenfeld AJ, Lehman RA, Hsu JR, et al. The effect of vehicle protection on spine injuries in military conflict. Spine J. 2012;12:843-8.
216. Ragel BT, Allred CD, Brevard S, Davis RT, Frank EH. Fractures of the thoracolumbar spine sustained by soldiers in vehicles attacked by improvised explosive devices. Spine. 2009;34:2400-5.
217. Ramasamy A, Harrisson S, Lasrado I, Stewart MP. A review of casualties during the Iraqi insurgency 2006--a British field hospital experience. Injury. 2009;40:493-7.
218. Roberts DC, Power DM, Stapley SA. A review of 10 years of scapula injuries sustained by UK military personnel on operations. J R Army Med Corps. 2018;164:30-34.

219. Rohena-Quinquilla IR, Rohena-Quinquilla FJ, Scully WF, Evanson JR. Femoral Neck Stress Injuries: Analysis of 156 Cases in a U.S. Military Population and Proposal of a New MRI Classification System. AJR Am J Roentgenol. 2018;210:601-607.
220. Schoenfeld AJ, Dunn JC, Belmont PJ. Pelvic, spinal and extremity wounds among combat-specific personnel serving in Iraq and Afghanistan (2003-2011): A new paradigm in military musculoskeletal medicine. Injury. 2013;44:1866-70.
221. Schoenfeld AJ, Goodman GP, Belmont Jr PJ. Characterization of combat-related spinal injuries sustained by a US Army Brigade Combat Team during Operation Iraqi Freedom. Spine J. 2012;12:771-6.
222. Taanila H, Suni J, Pihlajamäki H, Mattila VM, Ohrankämmen O, Vuorinen P, Parkkari J. Musculoskeletal disorders in physically active conscripts: a one-year follow-up study in the Finnish Defence Forces. BMC Musculoskelet Disord. 2009;10:89.
223. Webster CE, Clasper J, Gibb I, Masouros SD. Environment at the time of injury determines injury patterns in pelvic blast. J R Army Med Corps. 2019;165:15-17.
224. Wordsworth M, Thomas R, Breeze J, Evriviades D, Baden J, Hettiaratchy S. The surgical management of facial trauma in British soldiers during combat operations in Afghanistan. Injury. 2017;48:70-74.
225. Zachar MR, Labella C, Kittle CP, Baer PB, Hale RG, Chan RK. Characterization of mandibular fractures incurred from battle injuries in Iraq and Afghanistan from 2001-2010. J Oral Maxillofac Surg. 2013;71:734-42.
226. Zakowski B, Wagner I, Domzalski M. Analysis of a Military Parachutist Injury - A Retrospective Review of Over 37,000 Landings‡. Mil Med. 2019;184:e261-e265.
227. Andersen RC, Wilson KW, Bojescul JA, Mickel TJ, Gordon WT, Potter BK. Open, combat-related loss, or disruption of the knee extensor mechanism: treatment strategies, classification, and outcomes. J Orthop Trauma. 2014. 28:e250-7.
228. Ball VL, Sutton JA, Hull A, Sinnott BA. Traumatic injury patterns associated with static line parachuting. Wilderness Environ Med. 2014;25:89-93.
229. Bennett PM, Stevenson T, Sargeant ID, Mountain A, Penn-Barwell JG. Salvage of Combat Hindfoot Fractures in 2003-2014 UK Military. Foot Ankle Int. 2017;38:745-751.
230. Burns TC, Stinner DJ, Possley DR, Mack AW, Eckel TT, Potter BK, et al. Does the zone of injury in combat-related Type III open tibia fractures preclude the use of local soft tissue coverage? J Orthop Trauma. 2010;**24**:697-703.
231. Carmont MR, Patrick JH, Cassar-Pullicino VN, Postans NJ, Hay SM. Sequential metatarsal fatigue fractures secondary to abnormal foot biomechanics. Mil Med. 2006,171:292-7.
232. Carow SD, Houser JD. Trainees With Displaced Hip Fractures Present to Physical Therapy With Primary Complaint of Knee Pain. Mil Med. 2017;182:e2095-e2098.
233. Chalupa RL, Aberle C, Johnson AE. Observed Rates of Lower Extremity Stress Fractures After Implementation of the Army Physical Readiness Training Program at JBSA Fort Sam Houston. US Army Med Dep J. 2016:6-9.
234. Cho RI, Bakken HE, Reynolds ME, Schlifka BA, Powers DB. Concomitant cranial and ocular combat injuries during Operation Iraqi Freedom. J Trauma. 2009;67:516-20.
235. Clasper JC, Phillips SL. Early failure of external fixation in the management of war injuries. J R Army Med Corps. 2005;151:81-6.
236. Comat G, Barbier O, Ollat D. The posterior malleolar fracture: a parachute injury not to be overlooked. Orthop Traumatol Surg Res. 2014;100:419-22.
237. Commandeur J, Derksen RJ, MacDonald D, Breederveld R. Identical fracture patterns in combat vehicle blast injuries due to improvised explosive devices; a case series. BMC Emerg Med. 2012;12:12.
238. Covey DC, Lurate RB, Hatton CT. Field hospital treatment of blast wounds of the musculoskeletal system during the Yugoslav civil war. J Orthop Trauma.2000;14:278-86
239. Kim DK, Kim TH. Femoral neck shaft angle in relation to the location of femoral stress fracture in young military recruits: femoral head versus femoral neck stress fracture. Skeletal Radiology. 2021;50:1163-1168.
240. Dunn JC, Kusnezov NA, Koehler LR, Eisenstein ED, Kilcoyne KG, Orr JD, Mitchell JS. Radial Head Arthroplasty in the Active Duty Military Service Member With Minimum 2-Year Follow-Up. J Hand Surg Am. 2017;42:660.e1-660.e7.
241. Duran-Stanton AM, Kirk KL. "March fractures" on a female military recruit. Mil Med.2011;176:53-5.
242. Finestone A, Milgrom C, Wolf O, Petrov K, Evans R, Moran D. Epidemiology of metatarsal stress fractures versus tibial and femoral stress fractures during elite training. Foot Ankle Int. 2011;32:16-20.
243. Fisher RA, Esparza SD, Webber BJ, Pawlak MT, Nye NS, Tchandja JN, et al. Athletic Trainer Integration in US Air Force Basic Training. J Athl Train. 2017;52:1.

244. Formby PM, Wagner SC, Pisano AJ, Van Blarcum GS, Kang DG, Lehman Jr RA. Outcomes After Operative Management of Combat-Related Low Lumbar Burst Fractures. Spine. 2015;40:E1019-E1024.
245. Galvin JW, Dannenbaum IV JH, Tubb CC, Poepping TP, Grassbaugh JA, Arrington ED. Infection Rate of Intramedullary Nailing in Closed Fractures of the Femoral Diaphysis After Temporizing External Fixation in an Austere Environment. J Orthop Trauma. 2015;29:e316-20.
246. Garvin D, Thomson B, Mudge C. Predicting femoral neck stress fracture extent with bone scintigraphy. J Nucl Med. 2018;59:1623.
247. Gordon WT, O'Brien FP, Strauss JE, Andersen RC, Potter BK. Outcomes associated with the internal fixation of long-bone fractures proximal to traumatic amputations. J Bone Joint Surg Am. 2010;92:2312-8.
248. Greer MA. Incidence of metacarpal fractures in U.S. soldiers stationed in South Korea. J Hand Ther. 2008;21:137-41.
249. Griffis CE, Rocchi V, Cochran G, Kuhn KM. Return to Duty in Military Members Following Surgical Treatment of Incomplete Femoral Neck Fractures. J Surg Orthop Adv. 2018;27:312-316.
250. Gwinn DE, Tintle SM, Kumar AR, Andersen RC, Keeling JJ. Blast-induced lower extremity fractures with arterial injury: prevalence and risk factors for amputation after initial limb-preserving treatment. J Orthop Trauma. 2011;25:543-8.
251. Hamdi M, Zrig M, Bellesoued A, Khezami M, Chaabane B, Khechelfi S, et al. Stress fracture locations in military personnel. Tunis Med. 2007;85:137-42.
252. Hinsley DE, Phillips SL, Clasper JC. Ballistic Fractures During The 2003 Gulf Conflict - Early Prognosis And High Complication Rate. J R Army Med Corps. 2006;152:96.
253. Hoencamp R, Tan EC, Idenburg F, Ramasamy A, van Egmond T, Leenen LP, et al. Challenges in the training of military surgeons: experiences from Dutch combat operations in southern Afghanistan. Eur J Trauma Emerg Surg. 2014;40:421-428.
254. Holmgaard R, Duffy J, Warburg FE, Jensen L, Bonde C. Danish experience with free flaps in war wounds. Dan Med J. 2016;63:A5180.
255. Inklebarger J, Griffin M, Taylor MJ, Dembry RB. Femoral and tibial stress fractures associated with vitamin D insufficiency. J R Army Med Corps. 2014;160:61-3.
256. Islinger RB, Kuklo TR, McHale KA. A review of orthopedic injuries in three recent U.S. military conflicts. Mil Med. 2000;165:463-5.
257. Jacobs N, Rourke K, Rutherford J, Hicks A, Smith SR, Templeton P, et al. Lower limb injuries caused by improvised explosive devices: proposed 'Bastion classification' and prospective validation. Injury. 2014;45:1422-8.
258. Breeze J, Gensheimer W, DuBose JJ. Combat Facial Fractures Sustained During Operation Resolute Support and Operation Freedom’s Sentinel in Afghanistan. Mil Med. 2020;185:414-416.
259. Lichtenberger JP, Kim AM, Fisher D, Tatum PS, Neubauer B, Peterson PG, et al. Imaging of Combat-Related Thoracic Trauma - Blunt Trauma and Blast Lung Injury. Mil Med. 2018;183:e89-e96.
260. Johnson JD, Chachula LA, Bickley RJ, Anderson CD, Ryan PM. Return to Duty Following Open Reduction and Internal Fixation of Unstable Ankle Fractures in the Active Duty Population. Mil Med. 2019;184:e381-e384.
261. Joshi A, Kc BR, Shah BC, Chand P, Thapa BB, Kayastha N. Femoral neck stress fractures in military personnel. JNMA J Nepal Med Assoc. 2009;48:99-102.
262. Khan SU, Khan M, Khan AA, Murtaza B, Maqsood A, Ibrahim W, et al. Etiology and pattern of maxillofacial injuries in the Armed Forces of Pakistan. J Coll Physicians Surg Pak. 2007;17:94-7.
263. Kumar AR, Grewal NS, Chung TL, Bradley JP. Lessons from the modern battlefield: successful upper extremity injury reconstruction in the subacute period. J Trauma. 2009;67:752-7.
264. Lanier PJ, Speirs J, Koehler L, Bader J, Abdelgawad A, Waterman BR. Predictors of Persistent Pain After Fixation of Distal Clavicle Fractures in an Active Military Population. Orthop. 2018;41:e117-e126.
265. Lee CH, Huang GS, Chao KH, Jean JL, Wu SS. Surgical treatment of displaced stress fractures of the femoral neck in military recruits: a report of 42 cases. Arch Orthop Trauma Surg. 2003;123:527-33.
266. Lehman Jr RA, Paik H, Eckel TT, Helgeson MD, Cooper PB, Bellabarba C. Low lumbar burst fractures: a unique fracture mechanism sustained in our current overseas conflicts. Spine J. 2012;12:784-90.
267. Lo MC, Giffin RP, Pakulski KA, Davis WS, Bernstein SA, Wise DV. High-Mobility Multipurpose Wheeled Vehicle Rollover Accidents and Injuries to U.S. Army Soldiers by Reported Occupant Restraint Use, 1992-2013. Mil Med. 2017;182:e1782-e1791.
268. Mody RM, Zapor M, Hartzell JD, Robben PM, Waterman P, Wood-Morris R, et al. Infectious complications of damage control orthopedics in war trauma. J Trauma. 2009;67:758-61.

269. Mossadegh S, Midwinter M, Parker P. Developing a cumulative anatomic scoring system for military perineal and pelvic blast injuries. J R Army Med Corps. 2013;159:i40-4.
270. Mossadegh S, Tai N, Midwinter M, Parker P. Improvised explosive device related pelvi-perineal trauma: anatomic injuries and surgical management. J Trauma Acute Care Surg. 2012;73:S24-31.
271. Nelson TJ, Wall DB, Stedje-Larsen ET, Clark RT, Chambers LW, Bohman HR. Predictors of mortality in close proximity blast injuries during Operation Iraqi Freedom. J Am Coll Surg. 2006;202:418-22.
272. Pavlović M, Pejović J, Mladenović J, Čekanac R, Jovanović D, Karkalić R, et al. Ejection experience in Serbian Air Force, 1990-2010. Vojnosanit Pregl. 2014;71:531-3.
273. Phillips BN, Chun DW, Colyer M. Closed globe macular injuries after blasts in combat. Retina. 2013;33:371-9.
274. Polacek M, Småbrekke A. Displaced stress fracture of the femoral neck in young active adults. BMJ Case Rep. 2010.
275. Poopitaya S, Kanchanaroek K. Injuries of the thoracolumbar spine from tertiary blast injury in Thai military personnel during conflict in southern Thailand. J Med Assoc Thai. 2009;**92**:S129-34.
276. Pope RP. Injury surveillance and systematic investigation identify a rubber matting hazard for anterior cruciate ligament rupture on an obstacle course. Mil Med. 2002;167:359-62.
277. Salminen ST, Böstman OM, Kiuru MJ, Pihlajamäki HK. Bilateral femoral fatigue fracture: an unusual fracture in a military recruit. Clin Orthop Relat Res. 2007;456:259-63.
278. Stewart L, Shaikh F, Bradley W, Lu D, Blyth DM, Petfield JL, et al. Combat-Related Extremity Wounds: Injury Factors Predicting Early Onset Infections. Mil Med. 2019;184:83-91.
279. Thomas R, Wood AM, Watson J, Arthur CH, Nicol AM. Delay in diagnosis of neck of femur stress fracture in a female military recruit. J R Nav Med Serv. 2012;98:27-9.
280. Ucak M. Incidence and Severity of Maxillofacial Injuries During the Syrian Civil War in Syrian Soldiers and Civilians. J Craniofac Surg. 2019;30:992-995.
281. Ucak M. Shrapnel Injuries on Regions of Head and Neck in Syrian War. J Craniofac Surg. 2020;31:1191-1195.
282. Williams T, Puckett M, Denison G, Shin A, Gorman J. Acetabular stress fractures in military endurance athletes and recruits: incidence and MRI and scintigraphic findings. Skeletal Radiol. 2002;31:277-81.
283. Yanovich R, Milgrom R, Friedman E, Moran DS. Androgen receptor CAG repeat size is associated with stress fracture risk: a pilot study. Clin Orthop Relat Res. 2011;469:2925-31.
284. Clark HD. Military load carriage during prolonged marches on lower extremity mechanics: Influence of gender. State University of New York at Buffalo. 2013:57.
285. Mauntel TC. The influence of lower extremity biomechanics on biochemical markers of bone turnover during Army Cadet Basic Training. The University of North Carolina at Chapel Hill. 2016:174.
286. Trone DW. Assessment of Data Systems, Smoking and Injury, and Poor Training Outcomes in U.S. Military Recruit Populations. University of California, San Diego. 2011:127.
287. Zalneraitis BH, Huuki E, Benavides LC, Benavides JM. Relation of Vitamin D Level, BMI, and Location of Lower Extremity Stress Fractures in Military Trainees. Mil Med. 2023;188:e1970-e1974.
288. Eckard TG, Miraldi SF, Peck KY, Posner MA, Svoboda SJ, DiStefano LJ, et al. Association Between Automated Landing Error Scoring System Performance and Bone Stress Injury Risk in Military Trainees. J Athl Train. 2021.
289. Griffis CE, Pletta AM, Mutschler C, Ahmed AE, Lorimer SD. Proportion of Navy Recruits Diagnosed With Symptomatic Stress Fractures During Training and Monetary Impact of These Injuries. Clin Orthop Relat Res. 2022;480:2120-2121.
290. Burtis MT, Faillace J, Martin LF, Hermenau S. Scaphoid fracture detection in a military population: a standardized approach for medical referral. Mil Med. 2006;171:404-408.
291. Gam A, Goldstein L, Karmon Y, Mintser I, Grotto I, Guri A, et al. Comparison of Stress Fractures of Male and Female Recruits during Basic Training in the Israeli Anti-Aircraft Forces. Mil Med. 2005;170:710-2.
292. Schwartz O, Bulis S, Olsen CH, Glasberg E, Dudkiewicz I. The Association Between History of an Ankle Sprain and Traumatic Meniscal Injury Among Infantry Combat Soldiers in the Israeli Defense Forces: A Historical Cohort Study. Mil Med. 2020;185:e748-e754.
293. Shaffer RA, Rauh MJ, Brodine SK, Trone DW, Macera CA. Predictors of stress fracture susceptibility in young female recruits. Am J Sports Med. 2006;34:108-115.
294. Reis JP, Trone DW, Macera CA, Rauh MJ. Factors Associated with Discharge during Marine Corps Basic Training. Mil Med. 2007;172:936-41.
295. Abbott A, Wang C, Stamm M, Mulcahey MK. Part I: Background and Clinical Considerations for Stress Fractures in Female Military Recruits. Mil Med. 2023;188:86-92.
296. Abbott A, Wang C, Stamm M, Mulcahey MK. Part II: Risk Factors for Stress Fractures in Female Military Recruits. Mil Med. 2023;188:93-99.

297. Barbeau P, Michaud A, Hamel C, Rice D, Skidmore B, Hutton B, et al. Musculoskeletal Injuries Among Females in the Military: A Scoping Review. Mil Med. 2021;186:e903-e931.
298. Hamstra-Wright KL, Djelovic E, Payette J. The Relationship Between Stress Fractures and Bone Turnover Markers Is Unclear in Athletic and Military Populations: A Critically Appraised Topic. Int J Athl Ther Train. 2023;28:144-150.
299. Hughes JM, O'Leary TJ, Koltun KJ, Greeves JP. Promoting adaptive bone formation to prevent stress fractures in military personnel. Eur J Sport Sci. 2022;22:4-15.
300. Lovalekar M, Hauret K, Roy T, Taylor K, Blacker SD, Newman P, et al. Musculoskeletal injuries in military personnel-Descriptive epidemiology, risk factor identification, and prevention. J Sci Med Sport. 2021;24:963-969.
301. Nindl BC, Kyröläinen H. Editorial: Military human performance optimization: Contemporary issues for sustained and improved readiness. Eur J Sport Sci. 2022;22:1-3.
302. Shaw KA, Hattaway J, Villani N, Barkley C, O'Brien F, Jackson KL, et al. Surgically Treated Femoral Neck Stress Fractures Are Likely to Result in Military Separation During Basic Combat Training. Clin Orthop Relat Res. 2022;480:1692-1693.
303. Ring M, Friemert B, Hackenbroch C, Achatz G. Stressfrakturen im militärischen Kontext. Die Unfallchirurgie. 2023;126:856-862.
304. Shaw KA, Moreland CM, Hunt TJ, Barkley C, O'Brien F, Jackson KL. Femoral Neck Stress Fractures in Athletes and the Military. J Bone Joint Surg Am. 2022;104:473-482.
305. Wardle SL, O'Leary TJ, McClung JP, Pasiakos SM, Greeves JP. Feeding female soldiers: Consideration of sex-specific nutrition recommendations to optimise the health and performance of military personnel. J Sci Med Sport. 2021;24:995-1001.
306. Greeves JP, Beck B, Nindl BC, O'Leary TJ. Current risks factors and emerging biomarkers for bone stress injuries in military personnel. J Sci Med Sport. 2023;26:S14-s21.
307. Aweid B, Aweid O, Talibi S, Porter K. Stress fractures. Trauma. 2013;15:308-321.
308. Friedl K. Sex-specific considerations in stress fracture risk of military personnel. J Sci Med Sport. 2017;20.
309. Balazs GC, Polfer EM, Brelin AM, Gordon WT. High seas to high explosives: the evolution of calcaneus fracture management in the military. Mil Med. 2014;179:1228-35.
310. Balthrop PM, Nyland J, Roberts CS. Risk factors and musculoskeletal injuries associated with all-terrain vehicle accidents. J Emerg Med. 2009;36:121-31.
311. Belmont PJ, Owens BD, Schoenfeld AJ. Musculoskeletal Injuries in Iraq and Afghanistan: Epidemiology and Outcomes Following a Decade of War. J Am Acad Orthop Surg. 2016;24:341-8.
312. Beranger F, De Lesquen H, Aoun O, Roqueplo C, Meyrat L, Natale C, et al. Management of war-related vascular wounds in French role 3 hospital during the Afghan campaign. Injury. 2017;48:1906-1910.
313. Boden BP, Osbahr DC. High-risk stress fractures: evaluation and treatment. J Am Acad Orthop Surg. 2000;8:344-53.
314. Breeze J, Blanch R, Baden J, Monaghan AM, Evriviades D, Harrisson SE, et al. Skill sets required for the management of military head, face and neck trauma: a multidisciplinary consensus statement. J R Army Med Corps. 2018;164:133-138.
315. Brenner, A., Stress Fracture Implications within the IET Environment. Armor. 2007; **116**(6): 40-43.
316. Orejel Bustos A, Belluscio V, Camomilla V, Lucangeli L, Rizzo F, Sciarra T, et al. Overuse-Related Injuries of the Musculoskeletal System: Systematic Review and Quantitative Synthesis of Injuries, Locations, Risk Factors and Assessment Techniques. Sensors. 2021;21:2438.
317. Clasper J. The interaction of projectiles with tissues and the management of ballistic fractures. J R Army Med Corps. 2001;147:52-61.
318. Connolly M, Ibrahim ZR, Johnson ON. Changing paradigms in lower extremity reconstruction in war-related injuries. Mil Med Res. 2016;3.
319. Constantini N, Mann G, Nyska M, Mei-dan O, Even A, Kahn G. RISK FACTORS FOR STRESS FRACTURES AND OTHER ORTHOPEDIC INJURIES IN FEMALE INFANTRY RECRUITS. Clin J Sport Med. 2004;14:375-375.
320. DeFroda SF, Cameron KL, Posner M, Kriz PK, Owens BD. Bone Stress Injuries in the Military: Diagnosis, Management, and Prevention. Am J Orthop. 2017;46:176-183.
321. Dembowski SC, Tragord BS, Hand AF, Rohena-Quinquilla IR, Lee IE, Thoma DC, et al. Injury Surveillance and Reporting for Trainees with Bone Stress Injury: Current Practices and Recommendations. Mil Med. 2018;183:e455-e461.
322. Dixon S, Nunns M, House C, Rice H, Stiles V. Ankle joint kinematics influence risk of third metatarsal stress fracture in military recruits. Footwear Sci. 2013;5:122.

323. Epstein Y, Yanovich R, Moran DS, Heled Y. Physiological employment standards IV: integration of women in combat units physiological and medical considerations. Eur J Appl Physiol. 2013;113:2673-2690.
324. Epstein D, Markovitz E, Nakdimon I, Guinzburg A, Aviram E, Gordon B, et al. Injuries associated with the use of ejection seats: a systematic review, meta-analysis and the experience of the Israeli Air Force, 1990-2019. Injury. 2020;51:1489-1496.
325. Finestone A, Milgrom C. How stress fracture incidence was lowered in the Israeli army: a 25-yr struggle. Med Sci Sports Exerc. 2008;40:S623-9.
326. Flinn SD. Changes in stress fracture distribution and current treatment. Curr Sports Med Rep. 2002;1:272-7.
327. Friedl KE. Biomedical research on health and performance of military women: accomplishments of the Defense Women's Health Research Program (DWHRP). J Womens Health. 2005;14:764-802.
328. Friedl KE, Evans RK, Moran DS. Stress fracture and military medical readiness: bridging basic and applied research. Med Sci Sports Exerc. 2008;40:S609-22.
329. Green NM, Matthews JJ. The management of acute hip pain in the military: femoral neck stress fractures and tears of the acetabular labrum. J R Nav Med Serv. 2016;102:124-9.
330. Hauret KG, Jones BH, Bullock SH, Canham-Chervak M, Canada S. Musculoskeletal injuries description of an under-recognized injury problem among military personnel. Am J Prev Med. 2010;38:S61-70.
331. Hosey RG, Fernandez MM, Johnson DL. Evaluation and Management of Stress Fractures of the Pelvis and Sacrum. Orthop (Online). 2008;31:383-385.
332. Houston MN, Peck KY, Cameron KL, Feagin JA. O1 The incidence of ankle injuries in intramural and club sports at the united states military academy. Br J Sports Med. 2017;51.
333. Jacobs JM, Cameron KL, Bojescul JA. Lower extremity stress fractures in the military. Clin Sports Med. 2014;33:591-613.
334. Jones B, Hauret K. The incidence and risk factors for stress fractures and other injuries among U.S. Army trainees. J Sci Med Sport. 2017;20:S84-S85.
335. Joy SM. Predicting Lower Extremity Stress Fractures in Young Women Recruited into the Marine Corps. Clin J Sport Med. 2007;17:80-81.
336. Kessler DF. Running FASTER: Changing Running Technique to Reduce Stress Injuries. Int J Athl Ther Train. 2020;25:49-53.
337. Knapik JJ. United States Military Parachute Injuries: Part 2: Interventions Reducing Military Parachute Injuries in Training and Operations. J Spec Oper Med. 2019;19:109-113.
338. Knapik JJ, Bedno SA. Epidemiological Evidence and Possible Mechanisms for the Association Between Cigarette Smoking and Injuries (Part 1). J Spec Oper Med. 2018;18:108-112.
339. Knapik JJ, Reynolds KL, Hoedebecke KL. Stress Fractures: Etiology, Epidemiology, Diagnosis, Treatment, and Prevention. J Spec Oper Med. 2017;17:120-130.
340. Knapik JJ, Reynolds KL, Harman E. Soldier load carriage: historical, physiological, biomechanical, and medical aspects. Mil Med. 2004;169:45-56.
341. Martin N, Beck K, Conlon C, Smeele R, Mugridge O, McClung J, et al. Iron status and associations with aerobic performance and stress fracture risk during initial military training. J Sci Med Sport. 2017;20:S164-S165.
342. Nappo KE, Hoyt BW, Balazs GC, Nanos GP, Ipsen DF, Tintle SM, et al. Union Rates and Reported Range of Motion Are Acceptable After Open Forearm Fractures in Military Combatants. Clin Orthop Relat Res. 2019;477:2329-2331.
343. Moran DS, Evans RK, Hadad E. Imaging of Lower Extremity Stress Fracture Injuries. Sports Med. 2013;43:345-356.
344. Moran DS, Evans RK, Hadad E. Imaging of lower extremity stress fracture injuries. Sports Med. 2008;38:345-56.
345. Murray CK, Obremskey WT, Hsu JR, Andersen RC, Calhoun JH, et al. Prevention of infections associated with combat-related extremity injuries. J Trauma. 2011;71:S235-57.
346. Nunns M, Rice H, House C, Fallowfield J, Allsopp A, Stiles V, et al. A prospective study identifying risk factors for tibial stress fracture in Royal Marine recruits: initial findings. Footwear Sci. 2013;5:123.
347. Nye NS, Covey CJ, Sheldon L, Webber B, Pawlak M, Boden B, et al. Improving Diagnostic Accuracy and Efficiency of Suspected Bone Stress Injuries. Sports Health. 2016;8:278-283.
348. Orejel Bustos A, Belluscio V, Camomilla V, Lucangeli L, Rizzo F, Sciarra T, et al. Overuse-Related Injuries of the Musculoskeletal System: Systematic Review and Quantitative Synthesis of Injuries, Locations, Risk Factors and Assessment Techniques. Sensors. 2021;21.

349. Orr R, Pope R, Lopes TJ, Leyk D, Blacker S, Bustillo-Aguirre BS, et al. Soldier Load Carriage, Injuries, Rehabilitation and Physical Conditioning: An International Approach. Int J Environ Res Public Health. 2021;18.
350. Patel DS, Roth M, Kapil N. Stress fractures: diagnosis, treatment, and prevention. Am Fam Physician. 2011;83:39-46.
351. Pegrum J, Crisp T, Padhiar N. Diagnosis and management of bone stress injuries of the lower limb in athletes. BMJ. 2012;344.
352. Peris P. Stress fractures. Best Pract Res Clin Rheumatol. 2003;17:1043-61.
353. Ramasamy A, Hill AM, Hepper AE, Bull AM, Clasper JC. Blast mines: physics, injury mechanisms and vehicle protection. J R Army Med Corps. 2009;155:258-64.
354. Shaffer RA, Rauh MJ, Brodine SK, Trone DW, Macera CA. PREDICTORS OF STRESS FRACTURE YOUNG WOMEN RECRUITS. J Orthop Sports Phys Ther. 2005;35:A73-a74.
355. Riley DJ. Predictors of fitness test performance in young men. University of California, San Diego and San Diego State University. 2004:107.
356. Rome K, Handoll HH, Ashford RL. Interventions for preventing and treating stress fractures and stress reactions of bone of the lower limbs in young adults. Cochrane Database Syst Rev. 2005:Cd000450.
357. Schaffer, R. Stress fracture risk varies by race, sex. Endocrine Today. 2017: **15**(5): 7.
358. Schoenfeld AJ, Lehman Jr RA, Hsu JR. Evaluation and management of combat-related spinal injuries: a review based on recent experiences. Spine J. 2012;12:817-23.
359. Shaffer SW, Uhl TL. Preventing and Treating Lower Extremity Stress Reactions and Fractures in Adults. J Athl Train. 2006;41:466-9.
360. Shenoy K, Kim YH. The Military Medical System and Wartime Injuries to the Spine. Bull Hosp Jt Dis. 2020;78:42-45.
361. Weber JM, Vidt LG, Gehl RS, Montgomery T. Calcaneal stress fractures. Clin Podiatr Med Surg. 2005;22:45-54.
362. Mattila VM, Niva M, Kiuru M, Pihlajamäki H. Risk Factors for Bone Stress Injuries: A Follow-up Study of 102,515 Person-Years. Med Sci Sports Exerc. 2007;39:1061-6.
363. Alfort H, Von Kieseritzky J, Wilcke M. Finger fractures: Epidemiology and treatment based on 21341 fractures from the Swedish Fracture register. PLoS One. 2023;18:e0288506.
364. Baker HP, Dahm J, Schultz K, Portney D, Dillman D, Strelzow J. A comparison of the incidence of concomitant ipsilateral femoral neck fractures in ballistic versus blunt femur fractures. Eur J Orthop Surg Traumatol. 2023;33:843-850.
365. Critchley ML, Toomey C, Gabel L, Kenny SJ, Emery CA. Differences in Bone Mineral Density and Associated Factors in Dancers and Other Female Athletes. Appl Physiol Nutr Metab. 2024.
366. Olson A, Khan U, Wagner L, Davidson V, Diedring B, Bandovic I, et al. Low energy gunshot injuries: Does removal of retained bullet fragmentation at the time of internal fixation reduce the risk of fracture related infection? Injury. 2024;55:111423.
367. Rice H, Seynnes O, Werkhausen A. Effect of increased running speed and weight carriage on peak and cumulative tibial loading. Scand J Med Sci Sports. 2023;33:2516-2523.
368. Bass CR, Salzar RS, Lucas SR, Davis M, Donnellan L, Folk B, et al. Injury risk in behind armor blunt thoracic trauma. Int J Occup Saf Ergon. 2006;12:429-42.
369. Ekenman I, Milgrom C, Finestone A, Begin M, Olin C, Arndt T, et al. The role of biomechanical shoe orthoses in tibial stress fracture prevention. Am J Sports Med. 2002;30:866-70.
370. Kizaki K, Yamashita F, Mori D, Funakoshi N. Ankle Structures of Professional Soccer (Football) Players With Proximal Diaphyseal Stress Fractures of the Fifth Metatarsal. J Foot Ankle Surg. 2019;58:489-491.
371. Saita Y, Nagao M, Kawasaki T, Kobayashi Y, Kobayashi K, Nakajima H, et al. Range limitation in hip internal rotation and fifth metatarsal stress fractures (Jones fracture) in professional football players. Knee Surg Sports Traumatol Arthrosc. 2018;26:1943-1949.
372. Al-Hilli AB, Salih DS. Early or delayed surgical treatment in compound limb fractures due to high velocity missile injuries: a 5-year retrospective study from Medical City in Baghdad. Iowa Orthop J. 2010;30:94-8.
373. Bass E, French DD, Bradham DD, Rubenstein LZ. Risk-adjusted mortality rates of elderly veterans with hip fractures. Ann Epidemiol. 2007;17:514-9.
374. Breeze J, Gensheimer W, DuBose JJ. Combat Facial Fractures Sustained During Operation Resolute Support and Operation Freedom's Sentinel in Afghanistan. Mil Med. 2020;185:414-416.
375. Choi HJ, Cho HM. Multiple stress fractures of the lower extremity in healthy young men. J Orthop Traumatol. 2012;13:105-110.
376. Dussault MC, Smith M, Hanson I. Evaluation of trauma patterns in blast injuries using multiple correspondence analysis. Forensic Sci Int. 2016;267:66-72.

377. Eardley W, Bonner TJ, Gibb IE, Clasper JC. Spinal Fractures in Current Military Deployments. J R Army Med Corps. 2012;158:101.
378. Wang H, Frame J, Ozimek E, Reedstrom C, Leib D, Dugan E. LOAD CARRIAGE INCREASES MECHANICAL LOADING RATES DURING WALKING. Conference Proceedings of the Annual Meeting of the American Society of Biomechanics. 2010:920-921.
379. Hughes JM, Dickin DC, Wang H. The relationships between multiaxial loading history and tibial strains during load carriage. J Sci Med Sport. 2019;22:48-53.
380. Ireland AW, Kelly PJ, Cumming RG. Risk factor profiles for early and delayed mortality after hip fracture: Analyses of linked Australian Department of Veterans' Affairs databases. Injury. 2015;46:1028-35.
381. Justin GA, Turnage WA, Brooks DI, Davies BW, Ryan DS, Eiseman AS, et al. Orbital Fractures and Associated Ocular Injuries in Operation Iraqi Freedom and Operation Enduring Freedom Referred to a Tertiary Care Military Hospital and the Effect on Final Visual Acuity. Ophthalmic Plast Reconstr Surg. 2020;36:55-60.
382. Mathieu L, Bertani A, Gaillard C, Ollat D, Rigal S, Rongiéras F. Wartime upper extremity injuries: experience from the Kabul International Airport combat support hospital. Chir Main. 2014;33:183-8.
383. Milgrom C, Radeva-Petrova DR, Finestone A, Nyska M, Mendelson S, Benjuya N, et al. The effect of muscle fatigue on in vivo tibial strains. J Biomech. 2007;40:845-50.
384. Nair R, Abdool-Carrim AT, Robbs JV. Gunshot injuries of the popliteal artery. Br J Surg. 2000;87:602-7.
385. Norozy A, Motamedi MH, Ebrahimi A, Khoshmohabat H. Maxillofacial Fracture Patterns in Military Casualties. J Oral Maxillofac Surg. 2020;78:611.e1-611.e6.
386. Stern CA, Stockinger ZT, Todd WE, Gurney JM. An Analysis of Orthopedic Surgical Procedures Performed During U.S. Combat Operations from 2002 to 2016. Mil Med. 2019;184:813-819.
387. Wang H, Kia M, Dickin DC. Influences of load carriage and physical activity history on tibia bone strain. J Sport Health Sci. 2019;8:478-485.
388. Webster CE, Clasper J, Stinner DJ, Eliahoo J, Masouros SD. Characterization of Lower Extremity Blast Injury. Mil Med. 2018;183:e448-e453.
389. Weidauer LA, Binkley T, Vukovich M, Specker B. Greater Polar Moment of Inertia at the Tibia in Athletes Who Develop Stress Fractures. Orthop J Sports Med. 2014;2.
390. Critchley M, Toomey C, Lobos SM, Palacios-Derflingher L, Kenny SJ, Emery C. 124 Bone mineral density and associated factors: do young female dancers and other recreational sport athletes differ? Br J Sports Med. 2021;55:A50-A50.
391. Dallagi A, Khessairi Z, Kamoun S, Jlassi O, Amri A. P-226 Military occupational accidents. Occup Environ Med. 2023;80:A47-A47.
392. Double R, O'Leary T, Fraser W, Greeves J. Self-reported Hormonal Contraceptive Use In The British Armed Forces. Med Sci Sports Exerc. 2021;53:361-361.
393. Greeves JP. Reproductive health and stress fracture risk: a wearables solution. J Sci Med Sport. 2022;25:S3.
394. Guerriere KI, Hughes JM, Kusumpa S, Walker LA, Richardson MD, Taylor KM, et al. Associations Between Physical Fitness Test Scores And Tibial Bone Microarchitecture In Young Adults Entering Military Training. Med Sci Sports Exerc. 2021;53:127-127.
395. Nindl BC. Role of the insulin-like growth factor system in bone health and military relevance during military training. J Sci Med Sport. 2022;25:S3.
396. Popp KL, Taylor KM, Guerriere KI, Smith NI, Staab JS, Walker LA, et al. Prior Physical Activity Influences Changes In Tibial Bone Microarchitecture During U.S. Army Basic Combat Training. Med Sci Sports Exerc. 2021;53:112-113.
397. Sekel NM, Sterczala A, Krajewski K, Jabloner L, Martin B, Ahamed N, et al. Association Between DXA And HR-pQCT Measurements Of BMD In Active, Recruit-aged Men And Women. Med Sci Sports Exerc. 2021;53:129-129.
398. Hughes JM, Foulis SA, Taylor KM, Guerriere KI, Walker LA, Hand AF, et al. A prospective field study of U.S. Army trainees to identify the physiological bases and key factors influencing musculoskeletal injuries: a study protocol. BMC Musculoskelet Disord. 2019;20:282.
399. O'Leary TJ. Bone and calcium metabolism markers as an indicator of skeletal adaptation and stress fracture risk in military personnel. J Sci Med Sport. 2022;25:S4-S4.
400. Shaw KA, Hattaway J, Villani N, Barkley C, O'Brien F, Jackson KL, et al. Surgically Treated Femoral Neck Stress Fractures Are Likely to Result in Military Separation During Basic Combat Training. Clin Orthop Relat Res. 2022;480:1684-1691.
401. Alhabeeb AY, Konbaz F, Aleissa S, Alhamed GS, Alhowaish TS, Alhamadh MS, et al. Returning to Work After Traumatic Spine Fractures: Current Status in a Military Hospital. Mil Med. 2024.

402. Barkley C, Wong WK, Knapik JJ, Westrick RB. The Presence of Hip Joint Effusion on MRI Is Predictive of a Grade 4 Femoral Neck Stress Injury. Mil Med. 2023.
403. Carlson Jr RJ. MRI Predictive Model's Utility in a Recruit Training Environment for Tibia Stress Fractures. Med J. 2023:10-16.
404. Greenlee TA, Bullock G, Teyhen DS, Rhon DI. Can a Psychologic Profile Predict Successful Return to Full Duty After a Musculoskeletal Injury? Clin Orthop Relat Res. 2024;482:617-629.
405. Mueller C, Moreland CM, Jackson KL, Hensley D, Lacap A, Shaw KA. Pectoralis Major Tendon Tears During Airborne Operations: Are These Injuries Isolated? Mil Med. 2022.
406. Perez KG, Eskridge SL, Clouser MC, McCabe CT, Galarneau MR. A Focus on Non-Amputation Combat Extremity Injury: 2001-2018. Mil Med. 2022;187:e638-e643.
407. Rhon DI, Greenlee TA, Cook CE, Westrick RB, Umlauf JA, Fraser JJ. Fractures and Chronic Recurrence are Commonly Associated with Ankle Sprains: a 5-year Population-level Cohort of Patients Seen in the U.S. Military Health System. Int J Sports Phys Ther. 2021;16:1313-1322.
408. Sinnott B, Ray C, Weaver F, Gonzalez B, Chu E, Premji S, et al. Risk Factors and Consequences of Lower Extremity Fracture Nonunions in Veterans With Spinal Cord Injury. JBMR Plus. 2022;6:e10595.
409. Vuoncino M, Scheidt J, Kauvar DS. Association between time to revascularization and limb loss in military femoropopliteal arterial injuries. J Vasc Surg. 2023;78:1198-1203.
410. Ayan A, Örsçelik A. Bilateral Tibial Stress Injuries in Recreational Athletes of Army. / Rekreasyonel Asker Sporcularda Bilateral Tibia Stres Yaralanmaları. Turk J Sports Med. 2020;55:14-20.
411. Celtikci E, Yakar F, Celtikci P, Izci Y. Relationship between individual payload weight and spondylolysis incidence in Turkish land forces. Neurosurg Focus. 2018;45:E12.
412. Cross AM, Davis C, Penn-Barwell J, Taylor DM, De Mello WF, Matthews JJ. The incidence of pelvic fractures with traumatic lower limb amputation in modern warfare due to improvised explosive devices. J R Nav Med Serv. 2014;100:152-6.
413. Dussault MC, Hanson I, Smith MJ. Blast injury prevalence in skeletal remains: Are there differences between Bosnian war samples and documented combat-related deaths? Sci Justice. 2017;57:439-447.
414. Hawkinson MP, Tennent DJ, Belisle J, Osborn P. Outcomes of Lisfranc Injuries in an Active Duty Military Population. Foot Ankle Orthop. 2017;2.
415. Lee CH, Choi CH, Yoon SY, Lee JK. Posttraumatic stress disorder associated with orthopaedic trauma: a study in patients with extremity fractures. J Orthop Trauma. 2015;29:e198-202.
416. Mbelu M, Kayembe D, Mokassa L, Malemba JJ. Frequency of hyperlipasemia in patients with bone fractures on follow up at the Military General Hospital in camp Kokolo. Pan Afr Med J. 2020;37:314.
417. Oh JS, Tubb CC, Poepping TP, Ryan P, Clasper JC, Katschke AR, et al. Dismounted Blast Injuries in Patients Treated at a Role 3 Military Hospital in Afghanistan: Patterns of Injury and Mortality. Mil Med. 2016;181:1069-74.
418. Penn-Barwell JG, Sargeant ID, Penn-Barwell JG, Bennett PM, Fries CA, Kendrew JM, et al. Gun-shot injuries in UK military casualties - Features associated with wound severity. Injury. 2016;47:1067-71.
419. Petfield JL, Tribble DR, Potter BK, Lewandowski LR, Weintrob AC, Krauss M, et al. Is Bone Loss or Devascularization Associated With Recurrence of Osteomyelitis in Wartime Open Tibia Fractures? Clin Orthop Relat Res. 2019;477:789-801.
420. Schoenfeld AJ, Newcomb RL, Pallis MP, Cleveland III AW, Serrano JA, Bader JO, et al. Characterization of spinal injuries sustained by American service members killed in Iraq and Afghanistan: A study of 2,089 instances of spine trauma. J Trauma Acute Care Surg. 2013;74:1112-1118.
421. Siddique MK, Bhatti AM. A two-year experience of treating vascular trauma in the extremities in a military hospital. J Pak Med Assoc. 2013;63:327-30.
422. Stannard A, Morrison JJ, Scott DJ, Ivatury RA, Ross JD, Rasmussen TE. The epidemiology of noncompressible torso hemorrhage in the wars in Iraq and Afghanistan. J Trauma Acute Care Surg. 2013;74:830-4.
423. Nye NS, Covey CJ, Pawlak M, Olsen C, Boden BP, Beutler AI. Evaluating an Algorithm and Clinical Prediction Rule for Diagnosis of Bone Stress Injuries. Sports Health. 2020;12:449-455.
424. Alowais FA, Alfaqeeh FA, Alammar AK, Mortada H, Skef Z, Alshammari M, et al. Patterns and Characteristics of Intentional Self-inflicted Hand Injuries among Military Personnel: A Retrospective Study and Proposal of Treatment Algorithm. Plast Reconstr Surg Glob Open. 2022;10:e4648.
425. Garcia A, Kretzmer TS, Dams-O'Connor K, Miles SR, Bajor L, Tang X, et al. Health Conditions Among Special Operations Forces Versus Conventional Military Service Members: A VA TBI Model Systems Study. J Head Trauma Rehabil. 2022;37:E292-e298.
426. Gu W, Groves LL, McClellan SF. Patterns of concomitant traumatic brain injury and ocular trauma in US service members. Trauma Surg Acute Care Open. 2024;9:e001313.

427. O'Leary TJ, Wardle SL, Rawcliffe AJ, Chapman S, Mole J, Greeves JP. Understanding the musculoskeletal injury risk of women in combat: the effect of infantry training and sex on musculoskeletal injury incidence during British Army basic training. BMJ Mil Health. 2023;169:57-61.
428. Tsur N, Arbel Y, Abuhasira S, Permut Y, Lvovsky A, Protter N. A retrospective study of oral pathoses in Israeli military divers and non-divers: 2011-2020. Dent Traumatol. 2022;38:48-52.
429. Whittle RS. Distance travelled by military recruits during basic training is a significant risk factor for lower limb overuse injury. BMJ Mil Health. 2022;168:343-348.
430. O'leary TJ, Wardle SL, Gifford RM, Double RL, Reynolds RM, Woods DR, et al. Tibial Macrostructure and Microarchitecture Adaptations in Women During 44 Weeks of Arduous Military Training. J Bone Miner Res. 2021;36:1300-1315.
431. Ahmed SI, Burns TC, Landt C, Hayda R. Heterotopic ossification in high-grade open fractures sustained in combat: risk factors and prevalence. J Orthop Trauma. 2013;27:162-9.
432. Antikainen A, Patinen P, Päkkilä J, Tjäderhane L, Anttonen V. The types and management of dental trauma during military service in Finland. Dent Traumatol. 2018;34:87-92.
433. Azevedo L, Martins D, Veiga N, Fine P, Correia A. Dental Injuries in a Sample of Portuguese Militaries - A Preliminary Research. Mil Med. 2018;183:e591-e595.
434. Baggaley M, Esposito M, Xu C, Unnikrishnan G, Reifman J, Edwards WB. Effects of load carriage on biomechanical variables associated with tibial stress fractures in running. Gait Posture. 2020;77:190-194.
435. Banti M, Walter J, Hudak S, Soderdahl D. Improvised explosive device-related lower genitourinary trauma in current overseas combat operations. J Trauma Acute Care Surg. 2016;80:131-4.
436. Bar-Dayan Y, Bar-Dayan Y, Weisbroth M, Shemer J. Diverse Influences on Parachuting Injuries in Israel. J R Army Med Corps. 2000;146:81.
437. Becker T, Ashkenazi M. Incidence of reported dental trauma among soldiers during basic training. Mil Med. 2009;174:190-192.
438. Becker T, Ashkenazi M. A rubber-covered ceramic weapon reduces the incidence of dental trauma in recruits during combat basic training. Mil Med. 2011;176:1117-9.
439. Bennett PM, Sargeant ID, Myatt RW, Penn-Barwell JG. The management and outcome of open fractures of the femur sustained on the battlefield over a ten-year period. Bone Joint J. 2015;97-b:842-6.
440. Dua A, Patel B, Desai SS, Holcomb JB, Wade CE, Coogan S, et al. Comparison of military and civilian popliteal artery trauma outcomes. J Vasc Surg. 2014;59:1628-32.
441. Dutton JR, Kusnezov NA, Lanzi JT, Garcia ES, Pallis MP. The Success of Hip Arthroscopy in an Active Duty Population. Arthrosc: J Arthrosc Relat Surg. 2016;32:2251-2258.
442. Evans RK, Antczak AJ, Lester M, Yanovich RA, Israeli E, Moran DS. Effects of a 4-month recruit training program on markers of bone metabolism. Med Sci Sports Exerc. 2008;40:S660-70.
443. Forsberg JA, Pepek JM, Wagner S, Wilson K, Flint J, Andersen RC, et al. Heterotopic ossification in high-energy wartime extremity injuries: prevalence and risk factors. J Bone Joint Surg Am. 2009;91:1084-91.
444. Fox CJ, Perkins JG, Kragh Jr JF, Singh NN, Patel B, Ficke JR. Popliteal artery repair in massively transfused military trauma casualties: a pursuit to save life and limb. J Trauma. 2010;69:S123-34.
445. Galarneau MR, Woodruff SI, Dye JL, Mohrle CR, Wade AL. Traumatic brain injury during Operation Iraqi Freedom: findings from the United States Navy-Marine Corps Combat Trauma Registry. J Neurosurg. 2008;108:950-7.
446. Ge W, Mu J, Huang C. The GDF5 SNP is associated with meniscus injury and function recovery in male Chinese soldiers. Int J Sports Med. 2014;35:625-8.
447. Gifford SM, Aidinian G, Clouse WD, Fox CJ, Porras CA, Jones WT, et al. Effect of temporary shunting on extremity vascular injury: an outcome analysis from the Global War on Terror vascular injury initiative. J Vasc Surg. 2009;50:549-55.
448. Gray GC, Smith TC, Kang HK, Knoke JD. Are Gulf War veterans suffering war-related illnesses? Federal and civilian hospitalizations examined, June 1991 to December 1994. Am J Epidemiol. 2000;151:63-71.
449. Hadid A, Epstein Y, Shabshin N, Gefen A. Biomechanical Model for Stress Fracture-related Factors in Athletes and Soldiers. Med Sci Sports Exerc. 2018;50:1827-1836.
450. Ng YY, Seng KY, Ying MF. Training incidents in armored vehicles in the Singapore Armed Forces. Mil Med. 2003;168:165-71.
451. Mauntel TC, Marshall SW, Hackney AC, Pietrosimone BG, Cameron KL, Peck KY, et al. Trunk and Lower Extremity Movement Patterns, Stress Fracture Risk Factors, and Biomarkers of Bone Turnover in Military Trainees. J Athl Train. 2020;55:724-732.

452. Mündermann A, Stefanyshyn DJ, Nigg BM. Relationship between footwear comfort of shoe inserts and anthropometric and sensory factors. Med Sci Sports Exerc. 2001;33:1939-1945.
453. Murray CK, Hsu JR, Solomkin JS, Keeling JJ, Andersen RC, Ficke JR, et al. Prevention and management of infections associated with combat-related extremity injuries. J Trauma. 2008;64:S239-51.
454. Newman T, Croy T, Hart J, Saliba S. The effects of prophylactic ankle bracing on dynamic reach distance and obstacle course performance in military cadets. Mil Med. 2012;177:567-72.
455. Nunns M, Stiles V, Dixon S. The effects of standard issue Royal Marine recruit footwear on risk factors associated with third metatarsal stress fractures. Footwear Science. 2012;4:59-70.
456. O'Leary TJ, Walsh NP, Casey A, Izard RM, Tang JC, Fraser WD, et al. Supplementary Energy Increases Bone Formation during Arduous Military Training. Med Sci Sports Exerc. 2021;53:394-403.
457. Patzkowski JC, Blair JA, Schoenfeld AJ, Lehman RA, Hsu JR, Skeletal Trauma Research Consortium. Multiple associated injuries are common with spine fractures during war. Spine J. 2012;12:791-7.
458. Penn-Barwell JG, Bennett PM, Kay A, Sargeant ID, Fries CA, Cooper J, et al. Acute bilateral leg amputation following combat injury in UK servicemen. Injury. 2014;45:1105-10.
459. Schermann H, Eiges N, Sabag A, Kazum E, Albagli A, Salai M, et al. Estimation of Dog-Bite Risk and Related Morbidity Among Personnel Working With Military Dogs. J Spec Oper Med. 2017;17:51-54.
460. Ng YY, Seng KY, Ying MF. Training incidents in armored vehicles in the Singapore Armed Forces. Mil Med. 2003;168:165-171.
461. Sormaala MJ, Niva MH, Kiuru MJ, Mattila VM, Pihlajamäki HK. Stress injuries of the calcaneus detected with magnetic resonance imaging in military recruits. J Bone Joint Surg Am. 2006;88:2237-42.
462. Stark DB, Willis AK, Eshelman Z, Kang YS, Ramachandra R, Bolte IV JH, et al. Human Response and Injury Resulting from Head Impacts with Unmanned Aircraft Systems. SAE Technical Paper. 2019;63:29-64.
463. Vasquez KB, Brozoski FT, Logsdon KP, Chancey VC. Retrospective Analysis of Injuries in Underbody Blast Events: 2007-2010. Mil Med. 2018;183:347-352.
464. Williams ST, Lawrence PT, Miller KL, Crook JL, LaFleur J, Cannon GW, et al. A comparison of electronic and manual fracture risk assessment tools in screening elderly male US veterans at risk for osteoporosis. Osteoporos Int. 2017;28:3107-3111.
465. Yang J, Sun J, Luo F, Sun Q, Zhao L, Su N, et al. Peak BMD assessment in a Chinese infantry recruit group. Int J Sports Med. 2011;32:970-4.
466. Young KW, Kim JS, Cho JH, Kim HS, Cho HK, Lee KT. Paratrooper's ankle fracture: posterior malleolar fracture. Clin Orthop Surg. 2015;7:15-21.
467. Zadik Y, Levin L. Orofacial injuries and mouth guard use in elite commando fighters. Mil Med. 2008;173:1185-7.
468. Zadik Y, Levin L. Oral and facial trauma among paratroopers in the Israel Defense Forces. Dent Traumatol. 2009;25:100-2.
469. Devlin JD, Knapik JJ, Solomon Z, Hauret KG, Morris K, Carter R, et al. Incidence of admission to the Physical Training and Rehabilitation Programs in Initial Entry Training during fiscal year 2011. Mil Med. 2014;179:547-552.
470. Little JV, Eckard TG, DiStefano LJ, Cameron KL, Marshall SW, Padua DA. Association of Dynamic Knee Valgus and Bone Stress Injury in US Military Academy Cadets. J Sport Rehabil. 2023;32:797-801.
471. Eckard TG, Miraldi SF, Peck KY, Posner MA, Svoboda SJ, DiStefano LJ, et al. Automated Landing Error Scoring System Performance and the Risk of Bone Stress Injury in Military Trainees. J Athl Train. 2022;57:334-340.
472. Koltun KJ, Sekel NM, Bird MB, Lovalekar M, Mi Q, Martin BJ, et al. Tibial Bone Geometry Is Associated With Bone Stress Injury During Military Training in Men and Women. Front Physiol. 2022;13:803219.
473. Baxter ML, Baycroft C, Baxter GD. Lower limb injuries in soldiers: feasibility of reduction through implementation of a novel orthotic screening protocol. Mil Med. 2011;176:291-6.
474. Andreotti G, Lange JL, Brundage JF. The nature, incidence, and impact of eye injuries among US military personnel: implications for prevention. Arch Ophthalmol. 2001;119:1693-7.
475. Armed Forces Health Surveillance Center. Injuries associated with combat sports, active component, U.S. Armed Forces, 2010-2013. Msmr. 2014;21:16-8.
476. Belmont Jr PJ, McCriskin BJ, Hsiao MS, Burks R, Nelson KJ, Schoenfeld AJ. The nature and incidence of musculoskeletal combat wounds in Iraq and Afghanistan (2005-2009). J Orthop Trauma. 2013;27:e107-13.

477. Belmont Jr CP, Taylor CK, Mason CK, Shawen CS, Polly Jr LD, Klemme LW. Incidence, epidemiology, and occupational outcomes of thoracolumbar fractures among U.S. Army aviators. J Trauma. 2001;50:855-61.
478. Belmont Jr PJ, Thomas D, Goodman GP, Schoenfeld AJ, Zacchilli M, Burks R, et al. Combat musculoskeletal wounds in a US Army Brigade Combat Team during operation Iraqi Freedom. J Trauma. 2011;71:E1-7.
479. Davidson PL, Chalmers DJ, Wilson BD, McBride D. Lower limb injuries in New Zealand Defence Force personnel: descriptive epidemiology. Aust N Z J Public Health. 2008;32:167-73.
480. Dichiera R, Dunn J, Bader J, Bulken-Hoover J, Pallis M. Characterization of Metacarpal Fractures in a Military Population. Mil Med. 2016;181:931-4.
481. Fraser JJ, Ryans CP, MacGregor AJ, Janney CF. Macrotraumatic Fractures of the Multisegmented Ankle-Foot Complex in Military Tactical Athletes: A Cohort Study. J Am Podiatr Med Assoc. 2023;113.
482. Freedman BA, Serrano JA, Belmont PJ, Jackson KL, Cameron B, Neal CJ, et al. The combat burst fracture study-results of a cohort analysis of the most prevalent combat specific mechanism of major thoracolumbar spinal injury. Arch Orthop Trauma Surg. 2014;134:1353-1359.
483. Hsiao MS, Cameron KL, Huh J, Hsu JR, Benigni M, Whitener JC, et al. Clavicle fractures in the United States military: incidence and characteristics. Mil Med. 2012;177:970-4.
484. Jones BH, Canham-Chervak M, Canada S, Mitchener TA, Moore S. Medical surveillance of injuries in the u.s. Military descriptive epidemiology and recommendations for improvement. Am J Prev Med. 2010;38:S42-60.
485. Mitchener TA, Canham-Chervak M. Oral-maxillofacial injury surveillance in the Department of Defense, 1996-2005. Am J Prev Med. 2010;38:S86-93.
486. Pisquiy JJ, Carter JT, Chan A, Kusnezov N, Adler A. Incidence of Pelvic Ring Fractures in the U.S. Military Population. Cureus. 2020;12.
487. Qi RR, Wang JQ, Pan LL, Zhou W, Liu JL, Ju JT, et al. Descriptive epidemiology of deployment-related medical conditions and shipboard training-related injuries in a Chinese Navy population. Public Health. 2016;141:170-177.
488. Reynolds K, Cosio-Lima L, Creedon J, Gregg R, Zigmont T. Injury occurrence and risk factors in construction engineers and combat artillery soldiers. Mil Med. 2002;167:971-7.
489. Saad A, Kala C, Ohayon S, Feldman L, Galili E, Yanir Y, et al. Assessment of the Risk of Fractures Because of Service on Diesel Submarines: A Retrospective Cohort Study. Mil Med. 2015;180:787-91.
490. Schermann H, Gurel R, Ankory R, Kadar A, Yoffe V, Snir N, et al. Lower risk of fractures under methylphenidate treatment for ADHD: A dose-response effect. J Orthop Res. 2018;36:3328-3333.
491. Schoenfeld AJ, Laughlin MD, McCriskin BJ, Bader JO, Waterman BR, Belmont Jr PJ. Spinal injuries in United States military personnel deployed to Iraq and Afghanistan: an epidemiological investigation involving 7877 combat casualties from 2005 to 2009. Spine. 2013;38:1770-8.
492. Schoenfeld AJ, Romano D, Bader JO, Walker JJ. Lumbar spine fractures within a complete American cohort: epidemiology and risk factors among military service members. J Spinal Disord Tech. 2013;26:207-11.
493. Schoenfeld AJ, Sielski B, Rivera KP, Bader JO, Harris MB. Epidemiology of cervical spine fractures in the US military. Spine J. 2012;12:777-83.
494. Schram B, Pope R, Norman A, Orr R. A Detailed Analysis of Serious Personal Injuries Suffered by Full Time and Part Time Soldiers of the Australian Army. Mil Med. 2020;185:e364-e369.
495. Shere JL, Boole JR, Holtel MR, Amoroso PJ. An analysis of 3599 midfacial and 1141 orbital blowout fractures among 4426 United States Army Soldiers, 1980-2000. Otolaryngol Head Neck Surg. 2004;130:164-70.
496. Zigras F, Dellis S. Incidence and anatomic location of fractures resulting from static line parachuting in the Greek Army Forces: A retrospective study. J Res Pract Musculoskelet Syst. 2018;2:58-61.
