# Supplementary material for: Incidence and Risk Factors for the Development of Stress Fractures in Military Recruits and Qualified Personnel: A Systematic Review
Source: Int J Environ Res Public Health. 2025 Nov 20;22(11):1760. doi: 10.3390/ijerph22111760 (PMC12652357; doi:10.3390/ijerph22111760)
Supplement: Supplementary file 1 [file ijerph-22-01760-s001.zip › Supplementary Material File S4.pdf]

Supplementary Material S4: Characteristics and key findings of studies of stress fractures in military recruit/trainee populations

Study	Study Design	Participants	Methods (Diagnosis / Exposure to Risk Factors)	Occupations or occupational tasks: comparative levels of incidence or prevalence	Other contextual or risk factors	Study Quality Scores
Bar-Dayan et al. 2005 [67]<br><br>*Country of origin: Israel*	Prospective cohort	Ten mixed gender IDF anti-aircraft basic training recruit cohorts (N=513; *n* = 375 males; *n* = 138 females; age range 18-20 years) completing their 10 wk basic training course Nov 1999 – Jan 2003.	Injured recruits presented to the medical branch of the IDF and orthopaedic specialist examination was conducted, with radionuclide technetium bone scans to verify presence and severity of stress fracture.	Calculated overall *stress fracture* incidence rate was 1,713 new stress fractures per 1,000 person-years of exposure to basic training<br>Calculated overall *case* incidence rate was 75 individuals who suffered one or more stress fractures from the 513 recruits followed prospectively for 10 weeks, which equates to 760 cases per 1,000 person-years of recruit training exposure.	Calculated overall *stress fracture* incidence rate for male recruits was 1,248 new stress fractures per 1,000 person-years of exposure to basic training<br>Calculated overall *case* incidence rate for male recruits was 582 cases per 1,000 person-years of recruit training exposure.<br><br>Calculated overall *stress fracture* incidence rate for female recruits was 2,976 new stress fractures per 1,000 person-years of exposure to basic training<br>Calculated overall *case* incidence rate for female recruits was 1243 cases per 1,000 person-years of recruit training exposure.<br>Female recruits had a significantly higher stress fracture incidence rate than male recruits (IRR 2.38 (95% CI 1.89-3.00)	67%<br><br>Level of Evidence: II
Carswell et al., 2023 [87]<br><br>Country of Origin: United Kingdom	Prospective Cohort	British army recruits (N = 2167; *n* = 1637 males; *n* = 530 females; age 22.6 ± 7.5 years) volunteered between April 2013 – March 2017.	Injuries reported to the medical centre by participants during military training were diagnosed by clinicians and recorded in their medical records. Imaging was used to assess stress fracture where necessary (e.g., use of magnetic resonance imaging, X-ray).<br><br>Venous blood samples were assessed for vitamin D metabolites (serum- 25-dihydroxyvitamin D (25(OH)D), $1,25(OH)_2D$, and $24,25(OH)_2D$) in Week 1 of initial military training.	Overall calculated case incidence rate among British Army recruits (across Standard, Officer and Infantry initial training programs) was 242.8 cases with one or more diagnosed stress fractures per 1,000 person-years.	Calculated case-based incidence rate in male Army Officer training was 129.5 cases per 1,000 person-years. Calculated case-based incidence rate among women in Army Officer training was 163.9 cases per 1,000 person-years.<br><br>Calculated case-based incidence rate in male Infantry training was 257.2 cases per 1,000 person-years.<br><br>Calculated case-based incidence rate among women undergoing standard training was 338.5 cases per 1,000 person-years.<br><br>Concentrations of $24,25(OH)_2D$ between 0.4-3.1 nmol L^{-1} and 3.2-5.1 nmol L^{-1} (quartiles 1 and 2) at Week 1 of training were associated with increased odds (OR=4.02, 95% CI 1.82-8.87; and OR=2.39, 95% CI 1.16-4.92, respectively) of sustaining a stress fracture when compared with quartile 4 levels (7.7-29.6 nmol L^{-1}).	91%<br><br>Level of Evidence: II

Study	Study Design	Participants	Methods (Diagnosis / Exposure to Risk Factors)	Occupations or occupational tasks: comparative levels of incidence or prevalence	Other contextual or risk factors	Study Quality Scores
					Concentrations of 25(OH)D and the 1,25$(OH)_2$D:24,25$(OH)_2$D ratios in Cluster 1 (6.9-38.5 nmol L^{-1} and 125-307) and Cluster 3 (13.1-68.6 nmol L^{-1} and 17-63) were associated with increased odds (OR=22.08, 95% CI 3.26-149.4; and OR=5.00, 95% CI 1.20-20.81, respectively) of sustaining a stress fracture when compared with Cluster 6 (107.2-222.5 nmol L^{-1} and 6-32).<br><br>Concentrations of 24,25$(OH)_2$D between 0.4-3.1 nmol L^{-1} and 3.2-5.1 nmol L^{-1} (quartiles 1 and 2) at Week 1 of training were associated with an increased hazard ratio (HR=5.15, 95% CI 1.67-18.26; and HR=3.60, 95% CI 1.24-10.4, respectively) of sustaining a stress fracture when compared with quartile 4 levels (7.7-29.6 nmol L^{-1}).	
Claassen, Hu, & Rohrbeck (2014) [10]<br><br>*Country of origin: United States of America*	Retrospective cohort	Active U.S. military service members (Army, Navy, Air Force, Marine Corps, Coast Guard), recruits, and deployed members to OEF, OIF or OND. Surveillance period for recruit cohorts was generally 1/01/2003 – 31/12/2012; Coast guard periods were 2007 – 2012.<br><br>Only data relating to stress fractures in recruits are reported here.	Data from the Defense Medical Surveillance System, Theater Medical Data Store, and Transportation Command Regulating and Command and Control Evacuation System were queried for incident ICD-9 codes for fracture types: stress (733.93-.98) and pathologic (733.1) fractures.	Overall incidence rate for stress fractures (including pathologic fractures) in recruits from the U.S. Armed Forces was 44.2 new stress fractures per 1,000 person-years, from 2003-2012<br>**Incidence rates (per 1,000 person-years) of stress fractures (including pathologic fractures) in recruits from the U.S. Armed Forces by anatomical location**<br>**Anatomical location** — **Incidence rate**<br>Tibia or fibula — 15.7<br>Other bone — 15.2<br>Metatarsals — 5.6<br>Pelvis — 1.4<br>Femoral neck — 1.1<br>Femoral shaft — 0.7	Overall incidence rates for stress fractures in female recruits from the U.S. Armed Forces was 94.7 new stress fractures per 1,000 person-years, from 2003-2012<br>Overall incidence rates for stress fractures in male recruits in the U.S. Armed Forces was 29.6 new stress fractures per 1,000 person-years, from 2003-2012<br><br>**Incidence rates (per 1,000 person-years) of stress fractures in recruits from the U.S. Armed Forces by age-group**<br>**Age group** — **Incidence rate**<br>18-20 — 13.5<br>21-24 — 32.7<br>25-29 — 50.9<br>30-34 — 85.7<br>35+ — 120.8	67%<br><br>Level of Evidence: III-2
Constantini et al. 2010 [68]	Prospective cohort (with historical retrospective control group)	Israeli female border police recruits (*n* = 1,423), each undergoing 16 weeks	Bone stress fractures were diagnosed by clinical examination and bone scintigraphy.	The overall incidence rate of stress fractures across all recruits in both groups combined was 264 stress fractures in 1423 recruits in 16 weeks of training, which equates to a *stress fracture* incidence rate of	The overall calculated *stress fracture* incidence rate for recruits in the intervention group was 49 stress fractures in 213 recruits in 16 weeks of training, which equates to 748 stress fractures per	73%

Study	Study Design	Participants	Methods (Diagnosis / Exposure to Risk Factors)	Occupations or occupational tasks: comparative levels of incidence or prevalence	Other contextual or risk factors	Study Quality Scores
Country of origin: Israel		of basic training, and comprised of:<br>Prospective intervention group supplied with modified (reduced weight) battle equipment (M16 rifle, modified combat vest) from three consecutive basic training courses from November 2005-November 2006 (*n* = 213);<br>Historical control group from 21 basic training courses from 1996-2005 (total *n* = 1,210), split into 15 courses with partial data (*n* = 848) and 6 courses with full data (inclusive of anthropometric and menstrual data; *n* = 362).		603 stress fractures per 1,000 recruit-years of training.<br>The 264 stress fractures recorded affected 99 individual recruits during the 16 weeks of training, giving a *case* incidence rate of 226 cases (individuals) with one or more diagnosed stress fractures per 1,000 recruit-years of training.<br>Recruits who were diagnosed with stress fractures experienced an average of 2.7 stress fractures each during the 16 weeks of training.	1,000 recruit-years; overall 8% (17/213) of recruits suffered at least one stress fracture, giving a *case* incidence rate of 259 cases (individuals) with one or more diagnosed stress fractures per 1,000 recruit-years of training; and that 8% of recruits suffered an average of 2.9 stress fractures each.<br>The overall calculated *stress fracture* incidence rate for recruits in the historical control group was 215 stress fractures in 1210 recruits in 16 weeks of training, which equates to 577 stress fractures per 1,000 recruit-years; overall we calculated that 7% (82/1210) of recruits suffered at least one stress fracture, giving a *case* incidence rate of 220 cases (individuals) with one or more diagnosed stress fractures per 1,000 recruit-years of training; and that 7% of recruits suffered an average of 2.6 stress fractures each.<br>However, it should be noted that the study authors reported that 18.3% (rather than 7%, which we calculated) of recruits in the control group suffered at least one stress fracture, based on the same underpinning figures (82/1210), and noted this figure was significantly greater than the figure of 8% from the intervention group. It would therefore appear there is an error in their calculations, and that actually both the *stress fracture* incidence rate and *case* incidence rate in the intervention group were slightly higher than the proportion in the control group. This error may also have contributed to the OR the study authors reported to compare odds of stress fractures in each group (see below), which also appears unlikely given the underlying incidence rates and proportions of recruits injured.<br>The authors reported that the only variable significantly associated with an increased risk of stress fracture after adjusting for demographics was training with heavy equipment (Odds ratio 4.0, 95% CI, 2.1-7.6). However, this OR appears to be incorrect, when the respective incidence rates listed above, are considered. The incidence rates reported for the intervention group were	Level of Evidence: II

Study	Study Design	Participants	Methods (Diagnosis / Exposure to Risk Factors)	Occupations or occupational tasks: comparative levels of incidence or prevalence	Other contextual or risk factors	Study Quality Scores
					higher than the incidence rates in the control group.	
Cosman et al. 2013 [69]<br><br>*Country of origin: United States of America*	Prospective cohort	United States Military Academy (USMA) cadets (N = 891; *n* = 755 men, mean age 18.7 years; *n* = 136 women, mean age 18.4 years).<br><br>Intensive basic training comprised the first 2 months of the study period (4-years).	Cadets with possible stress fractures were clinically examined by orthopaedic surgeons and diagnosed using X-ray, radionuclide bone scan or MRI.	The overall incidence of *stress fractures* in the USMA cadets observed over the 4-year period was 98 stress fractures in the 891 recruits, equating to 27.5 stress fractures per 1,000 cadet-years. The *case* incidence rate was 69 cases (i.e. cadets with one or more stress fractures) among the 891 cadets across the 4 year period, equating to 19.3 cases per 1,000 cadet-years. However, the incidence rates reduced substantially in later years when compared to Year 1 – the study authors reported that 'more than 50%' of stress fractures occurred within the first three months of the USMA training period, and the approximate proportions of cadets who suffered stress fractures in each year of the 4-year training program, by sex, were as follows:<br><br>**Year** / **Proportion of male cadets** / **Proportion of female cadets**<br>1 / 3.8% / 14%<br>2 / 1.5% / 2.8%<br>3 / 0.4% / 2.7%<br>4 / 0.2% / 1.4%	Overall incidence of stress fractures in male cadets over the 4-year period was 60 stress fractures in the 755 male cadets, equating to 19.9 stress fractures per 1,000 cadet-years. The *case* incidence rate for male cadets was 43 cases among the 755 male cadets across the 4 year period of follow-up, equating to 14.2 cases per 1,000 cadet-years.<br><br>Overall incidence of stress fractures in female cadets over the 4-year period was 38 stress fractures in the 136 female cadets, equating to 69.9 stress fractures per 1,000 cadet-years. The *case* incidence rate for female cadets was 26 cases among the 136 female cadets across the 4 year period of follow-up, equating to 47.8 cases per 1,000 cadet-years.<br><br>**Adjusted relative risks for specified categories or changes in significant risk factors, stratified by sex**<br>**Exposure** / **Men** / **Women**<br><7 h/wk exercise hx in year prior (vs. >7h/wk) / 2.31 (1.29-4.12) / 1.27 (0.60-2.74)<br>Years since menarche (each yr ↓) / - / 1.44 (1.19-1.73)<br>Diameter of femoral neck (each mm ↓) / 1.35 (1.01-1.81)* / 1.16 (1.01-1.33)<br>Tibial bone mineral content (each 10 mg ↓) / 1.11 (1.03-1.20) / 1.03 (0.92-1.16)	67%<br><br>Level of Evidence: II

Study	Study Design	Participants	Methods (Diagnosis / Exposure to Risk Factors)	Occupations or occupational tasks: comparative levels of incidence or prevalence	Other contextual or risk factors	Study Quality Scores
					Tibial cortex cross-sectional area (each 10 mm^2 ↓) 1.12 (1.03-1.23) 1.01 (0.89-1.15)<br>**this relationship was no longer significant (CI crossed 1.00) when adjusted for race.*	
Cowan et al. 2012 [37]<br><br>*Country of origin: United States of America*	Retrospective cohort	U.S. Army female active-duty members (initially recruits) – tracked through initial 6 months of service and entering military February 2005 – September 2006 (*n* = 1568).	Participants took the Assessment of Recruit Motivation and Strength (ARMS) modified Harvard Step Test upon entering service. Only participants allocated as weight qualified according to weight-for-height standards set by the Army were included. Observation period was 180 days. Data sources were procured from the US Military Entrance Processing Command and Defense Manpower Data Center for participant demographics; and the Standard Ambulatory Data Record was queried using ICD-9 codes relating to stress fracture (i.e. 733.93-.95).	Overall case incidence rate for stress fractures in these weight-qualified female U.S. Army personnel was 225.8 cases with one or more stress fractures per 1,000 person-years of service	**Adjusted (for all included variables) incidence rate ratios (IRR) for categories of risk factors related to stress fractures in female army recruits**<br>**Risk factor / Category / IRR (95% CI)**<br>Step Test status: Pass (ref) 1.00; Fail 1.76 (1.18-2.63)<br>BMI: Underweight 2.63 (1.38-5.02); Normal (ref) 1.00; Overweight/obese 0.77 (0.48-1.21)<br>Age (years): 18-19 (ref) 1.00; 20-24 2.06 (1.32-3.20); ≥ 25 3.07 (1.81-5.19)<br>Smoker: No (ref) 1.00; Yes 1.41 (0.91-2.21)<br>Race: White (ref) 1.00; Black 0.68 (0.42-1.12); Other 0.97 (0.57-1.66)<br>Met ACSM adult activity standards: Yes (ref) 1.00; No 2.13 (1.04-4.36)	78%<br><br>Level of Evidence: III-2
Cowan et al. 2011 [38]	Retrospective cohort	U.S. Army male active-duty members (initially recruits) – tracked through initial	Participants took the Assessment of Recruit Motivation and Strength (ARMS) modified Harvard Step Test upon entering	**Case incidence rates (IR) and crude hazard ratio for bone stress fractures, by ARMS status**<br>**Over body fat (IR) / Weight qualified (IR) / Hazard ratio (95% CI)**		78%

Study	Study Design	Participants	Methods (Diagnosis / Exposure to Risk Factors)	Occupations or occupational tasks: comparative levels of incidence or prevalence	Other contextual or risk factors	Study Quality Scores
Country of origin: United States of America		90 days of service and entering military between February 2005 – September 2006. Split into weight-qualified (*n* = 6511) and over body fat (*n* = 812) groups, as per the entry standards.	service. Only recruits who passed the ARMS were enrolled and were allocated as over body fat or weight-qualified.<br>Stress fracture data were extracted from outpatient medical data from the US Army Medical Command patient Administration Systems and Biostatistics Activity, using ICD-9 codes.	84.0 cases with one or more stress fractures per 1,000 person-years of service · 65.7 cases with one or more stress fractures per 1,000 person-years of service · 1.25 (0.74-2.12)		Level of Evidence: III-2
Dash et al. 2012 [70]<br><br>*Country of origin: India*	Prospective cohort	Indian military recruits (male) completing 42 weeks of initial training from 2004 – 2009 (*n* = 8,570)	Stress fractures were diagnosed with clinical examination and radiological classification.	Overall stress fracture case incidence rate in these Indian military recruits was 87.3 cases (individuals with stress fractures) per 1,000 person-years of initial training<br>79% of stress fractures occurred during the 19-week basic training phase, while 20% occurred in the 15-week advanced training phase and 1% in the 8-week pre-training phase involving mainly education and minimal physical activity<br><br>**Anatomical location of stress fractures**<br>**Location** / **Percentage of cases**<br>Femur: 1.65%<br>Tibia: 88.57%<br>Fibula: 9.27%<br>Metatarsals: 0.5%	**Identified risk factors for stress fractures**<br>**Risk factor** / **Proportion with stress fractures** / ***p* value**<br>Urban class: 10.1%<br>Rural class: 4.8%<br>Hx of prior physical activity: 5.0%<br>No hx of physical activity: 7.6%<br>Vegetarian: 9.8%<br>Non-Vegetarian: 6.1%<br>*p* value: <0.001	56%<br><br>Level of Evidence: II
Davey et al. 2016 [71]<br><br>*Country of origin: United Kingdom*	Prospective cohort	Royal Marine recruits from 20 troops commencing 32 weeks of Royal Marine training between September 2009 - July 2010 (N = 1082)	Stress fracture diagnoses were made by a positive X-ray or MRI scan following complaints of symptoms consistent with potential stress fracture.<br><br>Status of serum 25-hydroxyvitamin D (25(OH)D) and parathyroid hormone (PTH) concentrations were assessed via venous blood samples taken at weeks 1, 15 and 32 of recruit training, to determine associations with stress fracture occurrence.	7.2% of the Royal Marine recruits (*n* = 78) experienced a total of 92 stress fractures during recruit training, giving a *stress fracture* incidence rate of 138.2 stress fractures per 1,000 person-years of Royal Marine training and a *case* incidence rate of 117.1 cases (individuals with one or more stress fractures) per 1,000 person-years of Royal Marine training	No statistically significant differences in mean baseline serum 25(OH)D or PTH were observed in recruits who sustained a stress fracture or did not (*p* >0.05)<br><br>Concentrations of 25(OH)D <50 nmol L^{-1} in recruits at week 1 of training were associated with an increased odds of sustaining a stress fracture when compared with 25(OH)D concentrations above these levels (OR=1.6, 95% CI 1.0-2.6)	78%<br><br>Level of Evidence: II

Study	Study Design	Participants	Methods (Diagnosis / Exposure to Risk Factors)	Occupations or occupational tasks: comparative levels of incidence or prevalence	Other contextual or risk factors	Study Quality Scores
Dixon et al. 2019 [72]<br><br>*Country of origin: United Kingdom*	Prospective cohort (with nested case-control)	UK Royal Marine (male) recruits (*N* = 1065) completing 32 weeks of training between September 2010 – July 2012.	Passive ankle dorsiflexion, dynamic peak ankle dorsiflexion, foot abduction, timing of heel-off, peak pressure magnitude, impulse, and timing at the metatarsal areas, and plantar pressures (dynamic arch index) during barefoot running were assessed during week 2 of training when recruits were injury-free.<br><br>2nd and 3rd metatarsal stress fractures diagnosed via MRI scan following report of symptoms during the subsequent 30 weeks of training were then recorded.	7 diagnoses of unilateral 2nd metatarsal stress fractures were recorded in the 1065 recruits, across the remaining 30 weeks of training following initial assessment in week 2. This equates to an incidence rate for 2nd metatarsal stress fractures during initial training of 11.4 2nd metatarsal stress fractures per 1,000 person-years of initial training.<br><br>14 diagnoses of unilateral 3rd metatarsal stress fractures were recorded in the 1065 recruits, across the remaining 30 weeks of training following initial assessment in week 2. This equates to an incidence rate for 3rd metatarsal stress fractures during initial training of 22.8 3rd metatarsal stress fractures per 1,000 person-years of initial training.	**Relative risk ratios (RR), derived by transforming odds ratios based on 21 metatarsal stress fracture cases and 150 controls, for risk factors significantly associated with 2nd (MT2) and 3rd (MT3) metatarsal stress fractures**<br>**Risk factor | MT2 RR | MT3 RR**<br>Dynamic arch index (for each 1% increase) | 0.75 (0.63-0.89) | 1.03 (0.95-1.11)<br>Age (for each 1-year increase) | 1.06 (0.85-1.32) | 0.78 (0.61-0.99)<br>Foot abduction (for each 1-degree increase) | 0.87 (0.80-0.96) | 1.09 (0.99-1.20)<br>Time of peak pressure at 2nd metatarsal (for each 1% increase in proportion of stance time) | 1.0 (0.86-1.17) | 1.19 (1.04-1.35)	56%<br><br>Level of Evidence: II
Eastman et al., 2023 [88]<br><br>Country of Origin: United Kingdom	Prospective Cohort	Male British Army infantry recruits (*n* = 201) undertaking either the Line Infantry or Parachute Regiment Training Courses, recruited between April 2014 – June 2016.	Diagnoses (metatarsal, cuneiform, calcaneus, tibia, pubic rami, femur, neck of femur) were retrieved from participants' medical records. Injuries were determined by magnetic resonance imaging and graded using the Fredericson scale.	Overall calculated stress fracture case incidence rate among British Army recruits was 194 cases per 1,000 person-years<br><br>**Stress fractures by anatomical location**<br>Location | No. (%) in total | No. (%) parachute regiment | No. (%) line Infantry<br>Tibia | 7 (35) | 7 (50) | 0 (0)<br>Calcaneus | 4 (20) | 4 (28.6) | 0 (0)<br>Metatarsal | 3 (15) | 1 (7.1) | 2 (33.3)<br>Cuneiform | 2 (10) | 0 (0) | 2 (33.3)<br>Pubic Rami | 1 (5) | 1 (7.1) | 0 (0)<br>Femur | 1 (5) | 0 (0) | 1 (16.7)<br>Femoral neck | 1 (5) | 1 (7.1) | 0 (0)<br>Not recorded | 1 (5) | 0 (0) | 1 (16.7)	Calculated case-based incidence rate of line infantry recruits was 87.6 cases per 1,000 person-years.<br><br>Calculated case-based incidence rate of parachute regiment recruits was 404 cases per 1,000 person-years.<br><br>Training regiment was significantly associated with odds of stress fracture incidence (parachute versus line infantry OR=748.9, 95% CI 6.85-81,866.10); and faster 2.4-km run time was significantly associated with increased odds of stress fracture incidence (OR=1.05, 95% CI 1.01-1.09), while age, body mass, lean body mass, height, leg areal bone mineral density, total 25(OH)D, peak power output, maximum strength, total area, cortical volumetric bone mineral density, trabecular thickness and cortical pore diameter were not.	64%<br><br>Level of Evidence: II
Evans, Guyver &	Retrospective cohort	British Royal Marine recruits 2001-2011 (*n* = 6,452) completing	Trauma records from the Princess Elizabeth Orthopaedic Centre and the Commando Training Centre	Overall incidence of displaced FNSF in these Royal Marine recruits completing commando training was 0.93 FNSF per 1,000 recruits		67%

Study	Study Design	Participants	Methods (Diagnosis / Exposure to Risk Factors)	Occupations or occupational tasks: comparative levels of incidence or prevalence	Other contextual or risk factors	Study Quality Scores
Hubble (2012) [39]<br><br>*Country of origin: United Kingdom*		commando training (a 32-week course according to course information provided online by the Royal Navy, accessed 9/12/2021).	Royal Marines electronic medical records were queried to identify all recruits in the 10-year period who suffered a displaced femoral neck stress fracture (FNSF).	undertaking the 32 weeks of training, equating to an estimated incidence rate of 1.51 FNSF per 1,000 person-years of training.		Level of Evidence: III-2
Finestone et al. 2004 [92]<br><br>*Country of origin: Israel*	Quasi-Experimental	Base 1: Male infantry recruits (*n* = 451) undergoing basic training for the Israel Defense Forces (IDF). Base 2: Infantry recruits (*n* = 423, all male) undergoing basic training for the Israel Defense Forces (IDF).	Recruits on base 1 were randomly assigned to either wearing a soft custom-made orthosis (n = 227; 204 with complete clinical follow-up) or a soft prefabricated orthosis (*n* = 224; 213 complete follow-up).<br>Recruits on base 2 were randomly assigned to wearing either semirigid biomechanical orthoses (*n* = 215; 180 complete follow-up) or semirigid prefabricated orthoses (*n* = 208; 172 complete follow-up).<br><br>Recruits were blinded as to type of orthoses. Recruits were monitored every 3 weeks for comfort, symptoms of injury.	**Calculated overall stress fracture *case* incidence rate across the 14 weeks of basic training:**<br><br>300 cases per 1,000 person-years of basic training	Incidence rates of stress fractures did not differ significantly by type of orthosis used	89%<br><br>Level of Evidence: III-2
Fisher et al. 2021 [90]<br><br>*Country of origin: United States of America*	Cluster randomised trial	U.S. Air Force recruits completing basic military training (8 weeks) January 2016 – December 2018 (N = 56,400), with a total of 1,206,445 (intervention group) and 2,173,218 (control group) training days, respectively.	Incidence rates of musculoskeletal injury (including stress fractures) were compared between an intervention arm receiving athletic trainer care (*n* = 20,810) and a control group (*n* = 35,590).<br><br>The study does not state how stress fractures were diagnosed; however, data were extracted using relevant ICD-10 codes from the Trainee Health Surveillance database and the Armed Forces Health Longitudinal Technology Application.	Case incidence rate for lower extremity stress fractures within the intervention arm was 0.24 cases per 1,000 training-days, equating to 87.6 cases per 1,000 person-years of training<br><br>Case incidence rate for lower extremity stress fractures within the control arm was 0.29 cases per 1,000 training-days, equating to 105.9 cases per 1,000 person-years of training	**Incidence rate ratio (95% CI) for lower extremity stress fractures in intervention vs. control arms:** 0.84 (0.73-0.97)	54%<br><br>Level of Evidence: II

Study	Study Design	Participants	Methods (Diagnosis / Exposure to Risk Factors)	Occupations or occupational tasks: comparative levels of incidence or prevalence	Other contextual or risk factors	Study Quality Scores
Griffis et al., 2022 [61]<br><br>Country of Origin: United States	Retrospective Cohort	US Navy recruits (male and female) entering Recruit Training Command between October 1, 2009 – September 30, 2015 (*N* = 204,774). Specific study population included 1098 recruits (male *n* = 604; female *n* = 494) diagnosed with stress fracture. Among these, 437 recruits provided Vitamin D levels.	Stress fractures were diagnosed when members presented to the medical facility and extracted using ICD-9 and ICD-10 codes 733.93 (stress fractures of the tibia), 733.94 (stress fracture of the metatarsal bone), 733.95 (stress fracture of the tibia and fibula), 733.96 (stress fracture of the femoral shaft), and 733.97 (stress fracture of the femoral neck).	0.5% of recruits sustained a stress fracture during the 5-year study period. This equates to a *case* incidence rate for stress fracture of 34.9 cases per 1,000 person-years for the total cohort.<br><br>**Number of Stress Fractures by Anatomical Location**<br><br>**Anatomical Location — % (n)**<br>Ankle — 0.5 (6)<br>Femur/Hip — 12 (135)<br>Fibula — 4 (47)<br>Metatarsal, Foot, Toe — 17 (189)<br>Pelvis — 2 (20)<br>Tibia and Fibula — 5 (57)<br>Tibia — 51 (562)<br>Unspecified or Other — 8 (84)	Recruits who had deficient Vitamin D levels (≤ 30 ng/mL) at the time they were diagnosed with a stress fracture had significantly higher physical therapy treatment costs than recruits with low-normal Vitamin D levels (31-40 ng/mL) at the time they were diagnosed ($p = 0.049$).	55%<br><br>Level of Evidence: III-2
Hauret, 2001 [40]<br><br>*Country of origin: United States of America*	Retrospective cohort	U.S. Army recruits completing basic combat training at Fort Jackson in the calendar year 1998 (N = 32,251; *n* = 20,858 men, *n* = 11,393 women).	Admissions of recruits who sustained injuries and were admitted to the Physical Training Rehabilitation Program (PTRP) by Army allied health professionals were recorded.<br><br>Bone stress fractures were diagnosed using radiographic findings or bone scintigraphy and all cases of stress fracture were admitted to the PTRP.	Overall incidence rate of stress fractures (all types) sustained by recruits completing basic combat training: 837 stress fractures in 32,251 recruits in 9 weeks of training, equating to 149.9 *stress fractures* per 1,000 person-years of training<br>Overall *case* incidence rate: 587 cases (individuals with one or more stress fractures) in 32,251 recruits in 9 weeks of training, equating to 105.2 *cases* per 1,000 person-years of training	Overall incidence of stress fractures sustained by *male* recruits completing basic combat training: 207 stress fractures in 20,858 male recruits in 9 weeks of training, equating to 57.3 stress fractures per 1,000 person-years of training<br>Overall male *case* incidence rate: 168 cases (male recruits with one or more stress fractures) in 20,858 male recruits in 9 weeks of training,, equating to 46.5 cases per 1,000 person-years of training<br><br>Overall incidence of stress fractures sustained by *female* recruits completing basic combat training: 630 stress fractures in 11,393 female recruits in 9 weeks of training, equating to 319.5 stress fractures per 1,000 person-years of training<br>Overall female *case* incidence rate: 419 cases (female recruits with one or more stress fractures) in 11,393 female recruits in 9 weeks of training, equating to 212.5 cases per 1,000 person-years of training<br><br>Case incidence rate ratio (female: male): 4.6 (95% CI 3.8-5.5)	67%<br><br>Level of Evidence: III-2

<table>
<tr><th>Study</th><th>Study Design</th><th>Participants</th><th>Methods (Diagnosis / Exposure to Risk Factors)</th><th>Occupations or occupational tasks: comparative levels of incidence or prevalence</th><th>Other contextual or risk factors</th><th>Study Quality Scores</th></tr>
<tr><td>Heagerty et al. 2018 [41]

Country of origin: United Kingdom</td><td>Retrospective cohort</td><td>British Army Infantry recruits (N = 10,498) recorded over four-years (2012 – 2016) to have commenced the 26-week Combat Infantry Course (CIC) at the Infantry Training Centre (ITC).</td><td>Injury (including stress fracture) data were extracted from the ITC Primary Care Rehabilitation Facility (PCRF) register of injury data, which were prospectively entered.</td><td>Overall incidence of stress fractures over the four-year period was 57.3 stress fractures per 1,000 recruits across the 26-week CIC, equating to 114.6 stress fractures per 1,000 person-years of CIC training

Stress fracture incidence by year (stress fractures per 1,000 person-years of CIC training)
<table><tr><th>Year</th><th>Recruit inflow (stress fractures) (n (n))</th><th>Incidence</th></tr><tr><td>2012/13</td><td>3521 (175)</td><td>99.4</td></tr><tr><td>2013/14</td><td>1922 (171)</td><td>178.0</td></tr><tr><td>2014/15</td><td>2543 (137)</td><td>107.8</td></tr><tr><td>2015/16</td><td>2512 (119)</td><td>94.8</td></tr></table></td><td></td><td>67%

Level of Evidence: III-2</td></tr>
<tr><td>Hetsroni et al. 2008 [74]

Country of origin: Israel</td><td>Prospective cohort</td><td>Male Israel infantry recruits undertaking 14 weeks of basic training (N = 473; N = 405 following dropout)</td><td>Biomechanical measures of subtalar joint kinematics were evaluated walking on a treadmill 2-weeks prior to training commencement.

Measured joint kinematic variables included maximal pronation angle, pronation range of motion, time from heel strike to maximum pronation, pronation mean angular velocity and time to maximum pronation.
Associations between these measures and tibia and femur stress fracture occurrence were assessed.
For these assessments, recruits were split into quartiles for the pronation measures, with Q1 representing the lower 25% of measured values; Q2 and Q3 representing the IQR, and Q4 representing the values in the upper quartile.</td><td>42 of the 405 completing recruits sustained stress fractures of the tibia or femur or both within the 14-week training period, equating to a case incidence rate for stress fractures of 385 cases (individuals experiencing one or more stress fractures) per 1,000 person-years of basic training.</td><td>Odds ratios (OR) comparing odds of stress fracture development between quartile groups based on pronation measures

There were no significant differences between groups for any pronation parameters except for time to maximum pronation/stance time, with those results listed below.

Time to maximum pronation/stance time (%)
<table><tr><th>Side</th><th>Groups (medians)</th><th>OR (95% CI)</th></tr><tr><td>L</td><td>Q4 (42.9) vs Q1 (19.3)</td><td>0.09 (0.02-0.40)</td></tr><tr><td>L</td><td>Q4 (42.9) vs IQR (26.9)</td><td>0.16 (0.04-0.69)</td></tr><tr><td>R</td><td>Q4 (41.9) vs Q1 (20.2)</td><td>0.29 (0.09-0.95)</td></tr><tr><td>R</td><td>Q4 (41.9) vs IQR (27.9)</td><td>0.23 (0.08-0.69)</td></tr></table></td><td>67%

Level of Evidence: II</td></tr>
</table>

Study	Study Design	Participants	Methods (Diagnosis / Exposure to Risk Factors)	Occupations or occupational tasks: comparative levels of incidence or prevalence	Other contextual or risk factors	Study Quality Scores
House, Reece & Roiz de Sa (2013) [42]<br><br>*Country of origin: United Kingdom*	Retrospective cohort	Two groups of Royal Marines (RM) recruits completing 32-weeks of commando basic training.<br>Group 1 (phase 1) were recruits commencing training between March 2004 – October 2005 (*n* = 1416).<br>Group 2 (phase 2) were recruits entering training from April 2006 – October 2007 (*n* = 1358)	Injuries were monitored in phase 1 in RM recruits wearing standard issue Saran insole military boot; and compared to phase 2 where recruits were issued with a shock absorbing insole (SAI) within the military boot.<br>Injury information were reported to the RM medical centre and entered into the Medical Centre database. Stress fracture diagnosis was confirmed with X-ray or MRI of the suspected site.	Overall incidence of stress fractures in the Saran insole (phase 1) group was 65.0 stress fractures per 1,000 recruits over the 32-week period of observation, equating to 105.6 stress fractures per 1,000 person-years of training<br>Overall incidence of stress fractures in the SAI (phase 2) group was 39.0 stress fractures per 1,000 recruits over the 32-week period of observation, equating to 63.4 stress fractures per 1,000 person-years of training<br><br>**Stress fracture incidence (stress fractures per 1,000 person-years) by anatomical location and insole type**<br>**Location / Saran Insole / SAI**<br>Tibia / 26.3 / 8.0<br>Femur / 13.8 / 8.0<br>Metatarsal / 68.9 / 54.0<br>Fibula / 0 / 2.3<br>Pubic bone / 0 / 1.1	The odds ratio comparing odds of sustaining a stress fracture in the Saran insole group with odds in the SAI group was 1.71 (95% CI, 1.21-2.42)<br><br>**Stress fracture odds ratio (95% CI) by anatomical location and insole type (Saran insole compared to SAI)**<br>**Location / Odds ratio**<br>Tibia / 3.19 (95% CI, 1.36-7.45)<br>Femur / 1.65 (95% CI, 0.65-4.2)<br>Metatarsal / 1.23 (95% CI, 0.84-1.82)<br>Fibula / Insufficient data<br>Pubic bone / Insufficient data	73%<br><br>Level of Evidence: III-2
Hughes et al. 2019 [43]<br><br>*Country of origin: United States of America*	Cohort study with nested case-control study, which used cohort data from the underlying cohort study to inform derivation of incidence rate ratios from the case-control element	Two separate cohorts. Cohort one was the full U.S. Army population from 2002 – 2011 (with *n* = 24,146 stress fracture cases in that time period). Cohort two was the subset of individuals in the Army at each timepoint in the study period that was undertaking the 11-week basic combat training course (BCT-only subgroup). The BCT-subgroup experienced *n* = 9088 reported stress fractures during the	Cases of incident stress fractures were identified through the Total Army Injury and Health Outcomes Database (TAIHOD) using ICD-9 codes (i.e. 733.14-.16, 733.94-.98). Selection of controls was based on matching each stress fracture case to four control soldiers (no history of stress fracture), with controls matched for date of occurrence of case's injury and length of time within army service.<br><br>Frequency of non-steroidal anti-inflammatory drugs intake by individuals within each cohort (Army and BCT-only subgroup) was the main exposure variable and defined as intake of an NSAID 30-180 days prior to		**Incidence rate ratios for stress fractures comparing case incidence in those with NSAID use to case incidence in those without NSAID use (sex-adjusted)**<br><br>**Prescription / BCT (IRR, 95% CI)**<br>All NSAIDs / 5.3 (4.9-5.7)<br>Ibuprofen / 4.5 (4.2-4.9)<br>Naproxen / 4.8 (4.1-5.8)	100%<br><br>Level of Evidence: III-2

Study	Study Design	Participants	Methods (Diagnosis / Exposure to Risk Factors)	Occupations or occupational tasks: comparative levels of incidence or prevalence	Other contextual or risk factors	Study Quality Scores
		time period of the study.	diagnosis of stress fracture. NSAID use was extracted from medical records of soldiers.<br><br>Recorded NSAIDs included ibuprofen, naproxen, meloxicam, and indomethacin. Use of paracetamol (acetaminophen) was also included.		Indomethacin 2.9 (1.8-4.7)<br><br>Paracetamol 4.4 (3.9-4.9)	
Itskoviz, Marom & Ostfeld (2011) [44]<br><br>*Country of origin: Israel*	Retrospective cohort	Israel Defense Forces (IDF) recruits undertaking 4 months of basic training 1998 – 2007 (N not provided)	Stress fracture cases in recruits completing basic training were identified within a standardised IDF health database.	The mean ± SD proportion of IDF recruits who experienced stress fractures during 4 months of basic training was 4.74% ± 2.16% (range 2.53 – 9.60%) in each year between 1998 and 2007, equating to a mean *case* incidence rate for stress fractures of 142.2 cases per 1,000 person-years of training<br><br>**Stress fractures by anatomical location**<br><br>**Location** / **Percentage contribution to total**<br>Tibia or Fibula 76.8<br>Metatarsal bones 14.3<br>Femur 7.9<br>Tarsal bones 0.8<br>Other 0.2	**Sex differences in stress fracture rates in recruits undertaking basic training (percentages of recruits who suffered stress fractures)**<br><br>**Year** / **Male** / **Female**<br>2004 4% 11%<br>2005 3% 8%<br>2006 3% 6%<br>2007 4% 7%<br><br>Estimated relative risk (female:male): 2.29	56%<br><br>Level of Evidence: III-2
Kardouni et al. 2021 [65]<br><br>*Country of origin: United States of America*	Retrospective cohort	U.S Army soldiers commencing service from 1/01/2005 – 31/12/2014 (*N*= 701,027; male *n* = 586,412; female *n* = 114,615).	The Total Army Injury and Health Outcomes Database (TAIHOD) was queried for ICD-9 codes relating to stress fractures (733.93-.98; 733.14, 733.16) which occurred during the initial 6-months of an individuals' service.	Overall incidence rate of stress fractures in the initial 6-months of service was 20.19 (95% CI 19.86-20.52) stress fractures per 1,000 soldiers, equating to 40.38 (95% CI 39.72-41.04) stress fractures per 1,000 person-years<br><br>**Weekly incidence of stress fractures (stress fractures per 1,000 soldiers) over the first 26 weeks of service**<br><br>**Week No.** / **Incidence (95% CI)**<br>1 0.09 (0.07-0.12)	Overall incidence rate of stress fractures for men in the initial 6-months of service was 13.71 (95% CI, 13.42-14.01) stress fractures per 1,000 soldiers, equating to 27.42 (95% CI 26.84-28.02) stress fractures per 1,000 person-years<br>Overall incidence rate of stress fractures for women in the initial 6-months of service was 53.33 (95% CI, 52.03-54.64) stress fractures per 1,000 soldiers, equating to 106.66 (95% CI 104.06-109.28) stress fractures per 1,000 person-years<br><br>**Female: male stress fracture IRR: 3.89**<br><br>**Stress fracture distribution by anatomical location, by sex (% [n])**<br>**Location** / **Male** / **Female**	78%<br><br>Level of Evidence: III-2

Study	Study Design	Participants	Methods (Diagnosis / Exposure to Risk Factors)	Occupations or occupational tasks: comparative levels of incidence or prevalence	Other contextual or risk factors	Study Quality Scores
				2 0.42 (0.38-0.47)	Tibia/fibula 34.9% (2,805) 19.2% (1,173)	
				3 1.05 (0.98-1.13)	Metatarsal 13.8% (1,109) 5.2% (320)	
				4 1.57 (1.48-1.66)	Femoral shaft 3.6% (290) 2.9% (176)	
				5 1.74 (1.64-1.84)	Femoral neck 9.2% (737) 11.4% (695)	
				6 1.64 (1.54-1.73)	Pelvis 3.7% (295) 16.9% (1,036)	
				7 1.73 (1.63-1.82)	Unspecified /other 34.9% (2,806) 44.8% (2,713)	
				8 1.74 (1.64-1.84)		
				9 1.46 (1.37-1.55)	**Weekly incidence of stress fractures (stress fractures per 1,000 men/women) over the first 26 weeks of service by sex**	
				10 1.17 (1.08-1.25)	**Week No. Male Female**	
				11 1.31 (1.22-1.40)	1 0.05 0.29	
				12 1.14 (1.06-1.22)	3 0.74 2.85	
				13 1.05 (0.97-1.13)	5 1.23 4.39	
				14 0.92 (0.84-0.99)	7 1.20 4.54	
				15 0.83 (0.76-0.91)	9 1.07 3.55	
				16 0.69 (0.62-0.75)	11 0.96 3.26	
				17 0.54 (0.49-0.60)	13 0.65 3.23	
				18 0.53 (0.48-0.59)	15 0.53 2.53	
				19 0.38 (0.33-0.43)	17 0.35 1.73	
				20 0.39 (0.34-0.44)	19 0.23 1.38	
				21 0.33 (0.29-0.38)	21 0.19 1.16	
				22 0.27 (0.23-0.31)	23 0.17 0.79	
				23 0.25 (0.21-0.30)	25 0.11 0.46	
				24 0.17 (0.14-0.21)		
				25 0.16 (0.12-0.20)		
				26 0.08 (0.05-0.12)		
				**Stress fracture distribution by anatomical location**		
				**Location of fracture Proportion % (n)**		
				Tibia/fibula 28.1% (3,978)		
				Metatarsal 10.1% (1,429)		
				Femoral shaft 3.3% (466)		
				Femoral neck 10.1% (1,432)		
				Pelvis 9.4% (1,331)		
				Unspecified/other 39.0% (5,519)		

Study	Study Design	Participants	Methods (Diagnosis / Exposure to Risk Factors)	Occupations or occupational tasks: comparative levels of incidence or prevalence	Other contextual or risk factors	Study Quality Scores
Kelly et al., 2024 [63]<br><br>Country of Origin: United States	Retrospective Cohort	Male U.S Marine Corps recruits (N = 3044; *n* = 2,363 OLC program; *n* = 681 MLC program) completing training in 2020 ((original load carriage) (OLC)) and 2021 ((modified load carriage (MLC)).	Data were extracted from the sports medicine injury database and stratified using ICD-10 codes.	Total number of stress fractures experienced in OLC = 114; equating to a period prevalence rate for stress fractures in OLC condition = 4.83%<br><br>Total number of stress fractures experienced in MLC = 9; equating to a period prevalence rate for stress fractures in MLC condition = 1.32%	RR OLC:MLC = 3.66 (95% CI, 1.86-7.16)	55%<br><br>Level of Evidence: III-2
Knapik et al. 2012 [45]<br><br>*Country of origin: United States of America*	Retrospective cohort	U.S. Army recruit population (N = 583,651; *male n* = 475,745, age 21 ± 3 yrs, height 175.6 ± 6.9 cm, weight 76.1 ± 13.3 kg; *female n* = 107,906, age 21 ± 4 yrs, height 162.7 ± 6.4 cm, weight 61.8 ± 9.5 kg) undertaking 10 weeks of basic training (including in-processing time in the reception station) within the years January 1997 – December 2007 (11-years).	The Defense Medical Surveillance System (DMSS) was queried for ICD-9 codes relating to pathological fractures or stress fractures (733,1-.19; 733.93-.98), with the 'pathological' fractures nearly all likely to have been stress fractures recorded under the 'pathological fracture' code due to administrative requirements.<br><br>Descriptive statistics relating to individual recruits included in this study were extracted from the Defense Manpower Data Centre (DMDC) Master Personnel File and/or the Military Entrance Processing Station (MEPS) database.	Overall calculated *case* incidence rate for stress/pathological fracture in the recruit cohort: 17,804 cases in 583,651 recruits across 10 weeks of basic training, equating to 30.5 cases per 1,000 recruits across 10 weeks of training, *or* 158.6 cases per 1,000 person-years of basic training	**Stress fracture *case* incidence (cases per 1,000 recruits across 10 weeks of training) by sex, and odds ratio comparing risk for women with that for men**<br>**Men** · **Women** · **Odds ratio (95% CI)**<br>6.9 · 26.1 · 3.85 (3.66-4.05)<br>**Pathological fracture *case* incidence (cases per 1,000 recruits across 10 weeks of training) by sex, and odds ratio comparing risk for women with that for men**<br>**Men** · **Women** · **Odds ratio (95% CI)**<br>13.4 · 59.9 · 4.71 (4.54-4.88)<br>**Stress & pathological fracture *case* incidence (cases per 1,000 recruits across 10 weeks of training) by sex, and odds ratio comparing risk for women with that for men**<br>**Men** · **Women** · **Odds ratio (95% CI)**<br>19.3 · 79.9 · 4.71 (4.28-4.54)<br><br>**Univariate estimates of *case* incidence rates for stress and pathological fractures (cases per 1,000 recruits across 10 weeks of training), by age, BMI, and race/ethnicity, stratified by sex**<br>**Age** · **Men** · **Women**<br>< 20 years · 14.0 · 64.0<br>20-24 · 20.2 · 86.5	89%<br><br>Level of Evidence: III-2

<table>
<tr><th>Study</th><th>Study Design</th><th>Participants</th><th>Methods (Diagnosis / Exposure to Risk Factors)</th><th>Occupations or occupational tasks: comparative levels of incidence or prevalence</th><th>Other contextual or risk factors</th><th>Study Quality Scores</th></tr>
<tr><td></td><td></td><td></td><td></td><td></td><td>
<table>
<tr><td>25-29</td><td>31.4</td><td>105.6</td></tr>
<tr><td>≥ 30</td><td>45.5</td><td>129.2</td></tr>
<tr><td>BMI (kg/m^2)</td><td>Men</td><td>Women</td></tr>
<tr><td>< 18.5</td><td>30.9</td><td>100.4</td></tr>
<tr><td>18.5-24.9</td><td>18.2</td><td>80.5</td></tr>
<tr><td>25.0-29.9</td><td>19.9</td><td>75.2</td></tr>
<tr><td>≥ 30</td><td>20.6</td><td>77.2</td></tr>
<tr><td>Race / Ethnicity</td><td>Men</td><td>Women</td></tr>
<tr><td>White</td><td>21.0</td><td>90.5</td></tr>
<tr><td>Black</td><td>12.2</td><td>60.9</td></tr>
<tr><td>Hispanic</td><td>19.3</td><td>81.5</td></tr>
<tr><td>Asian</td><td>17.0</td><td>76.8</td></tr>
<tr><td>Am. Indian</td><td>21.4</td><td>81.3</td></tr>
<tr><td>Other</td><td>25.8</td><td>102.8</td></tr>
<tr><td>Unknown</td><td>16.0</td><td>74.1</td></tr>
</table>
Multivariate estimates of odds ratios (OR) and 95% CI, indicating comparative risks of stress and pathological fracture, by age, BMI, and race/ethnicity, stratified by sex
<table>
<tr><td>Age</td><td>Men</td><td>Women</td></tr>
<tr><td>< 20 years</td><td>1.00 (ref)</td><td>1.00 (ref)</td></tr>
<tr><td>20-24</td><td>1.47 (1.40-1.54)</td><td>1.41 (1.34-1.48)</td></tr>
<tr><td>25-29</td><td>2.33 (2.19-2.49)</td><td>1.80 (1.67-1.93)</td></tr>
<tr><td>≥ 30</td><td>3.50 (3.20-3.82)</td><td>2.29 (2.09-2.51)</td></tr>
<tr><td>BMI (kg/m^2)</td><td>Men</td><td>Women</td></tr>
<tr><td>< 18.5</td><td>1.78 (1.60-1.98)</td><td>1.31 (1.19-1.45)</td></tr>
<tr><td>18.5-24.9</td><td>1.00 (ref)</td><td>1.00 (ref)</td></tr>
<tr><td>25.0-29.9</td><td>0.98 (0.94-1.03)</td><td>0.87 (0.83-0.92)</td></tr>
<tr><td>≥ 30</td><td>0.98 (0.92-1.05)</td><td>0.82 (0.68-0.99)</td></tr>
<tr><td>Race / Ethnicity</td><td>Men</td><td>Women</td></tr>
</table>
</td><td></td></tr>
</table>

Study	Study Design	Participants	Methods (Diagnosis / Exposure to Risk Factors)	Occupations or occupational tasks: comparative levels of incidence or prevalence	Other contextual or risk factors	Study Quality Scores
					White 1.74 (1.62-1.87) 1.54 (1.46-1.63)<br>Black 1.00 (ref) 1.00 (ref)<br>Hispanic 1.58 (1.44-1.73) 1.40 (1.30-1.52)<br>Asian 1.29 (1.12-1.48) 1.23 (1.08-1.41)<br>Am. Indian 1.80 (1.46-2.21) 1.39 (1.16-1.65)<br>Other 2.08 (1.48-2.92) 1.78 (1.30-2.44)<br>Unknown 1.24 (0.99-1.55) 1.20 (0.98-1.46)	
Knapik, Sharp & Montain (2018) [46]<br><br>*Country of origin: United States of America*	Retrospective cohort	This study comprised further analysis of the population of U.S. Army recruits completing basic combat training considered in the preceding (2012), study (N = 583,651; *male n* = 475,745, age 21 ± 3 yrs, height 175.6 ± 6.9 cm, weight 76.1 ± 13.3 kg; *female n* = 107,906, age 21 ± 4 yrs, height 162.7 ± 6.4 cm, weight 61.8 ± 9.5 kg) undertaking 10 weeks of basic training (including in-processing time in the reception station) within the years January 1997 – December 2007 (11-years).	The Defense Medical Surveillance System (DMSS) was queried for ICD-9 codes relating to pathological fractures or stress fractures (733,1-.19; 733.93-.98), with the 'pathological' fractures nearly all likely to have been stress fractures recorded under the 'pathological fracture' code due to administrative requirements.<br>Descriptive statistics relating to individual recruits included in this study were extracted from the Defense Manpower Data Centre (DMDC) Master Personnel File and/or the Military Entrance Processing Station (MEPS) database.	The incidence rate for stress/pathological fractures in this population has been reported immediately above, in the preceding related study.	**Multivariate associations between stress fracture risks, and percentage body fat level, and race/ethnicity in male recruits**<br>**Body fat %** | **Case incidence (cases per 1,000 recruits across 10 weeks of training)** | **Odds ratio (95% CI)**<br>< 8.90% 23.7 1.27 (1.16-1.39)<br>8.90-10.62% 18.2 0.98 (0.89-1.07)<br>10.63-12.08% 16.6 0.89 (0.81-0.98)<br>12.09-13.51% 16.5 0.89 (0.80-0.97)<br>13.52-15.05% 16.6 0.89 (0.81-0.99)<br>15.06-16.73% 18.7 1.00 (ref)<br>16.74-18.60% 20.0 1.07 (0.97-1.17)	78%<br><br>Level of Evidence: III-2

Study	Study Design	Participants	Methods (Diagnosis / Exposure to Risk Factors)	Occupations or occupational tasks: comparative levels of incidence or prevalence	Other contextual or risk factors			Study Quality Scores
					18.61-20.61%	20.4	1.08 (0.99-1.19)	
					20.62-23.52%	21.1	1.13 (1.03-1.23)	
					≥ 23.53%	21.4	1.17 (1.07-1.28)	
					**Race / Ethnicity**	**Case incidence (cases per 1,000 recruits across 10 weeks of training)**	**Odds ratio (95% CI)**	
					Black	12.2	1.00 (ref)	
					White	21.0	1.72 (1.60-1.85)	
					Hispanic	19.3	1.56 (1.43-1.71)	
					Asian	17.0	1.38 (1.20-1.59)	
					Am. Indian	21.8	1.75 (1.42-2.15)	
					Other	25.8	2.12 (1.52-2.98)	
					Unknown	16.0	1.31 (1.04-1.64)	
					**Multivariate associations between stress fracture risks, and percentage body fat levels, and race/ethnicity in female recruits**			
					**Body fat %**	**Case incidence (cases per 1,000 recruits across 10 weeks of training)**	**Odds ratio (95% CI)**	
					< 22.90%	90.1	1.22 (1.11-1.35)	
					22.90-24.56%	81.4	1.09 (0.98-1.20)	

Study	Study Design	Participants	Methods (Diagnosis / Exposure to Risk Factors)	Occupations or occupational tasks: comparative levels of incidence or prevalence	Other contextual or risk factors			Study Quality Scores
					24.57-25.97%	80.2	1.06 (0.96-1.18)	
					25.98-27.23%	79.6	1.05 (0.95-1.16)	
					27.24-28.53%	76.6	1.00 (ref)	
					28.54-29.55%	81.1	1.07 (0.97-1.18)	
					29.56-30.78%	80.8	1.06 (0.96-1.17)	
					30.79-32.21%	72.6	0.95 (0.86-1.06)	
					32.22-34.20%	77.4	1.03 (0.93-1.13)	
					≥ 34.21%	79.2	1.07 (0.97-1.18)	
					**Race / Ethnicity**	**Case incidence (cases per 1,000 recruits across 10 weeks of training)**	**Odds ratio (95% CI)**	
					Black	60.9	1.00 (ref)	
					White	90.5	1.54 (1.46-1.63)	
					Hispanic	81.5	1.37 (1.27-1.48)	
					Asian	76.8	1.28 (1.12-1.46)	
					Am. Indian	81.3	1.37 (1.15-1.64)	
					Other	102.8	1.77 (1.29-2.43)	
					Unknown	74.1	1.23 (1.01-1.51)	

Study	Study Design	Participants	Methods (Diagnosis / Exposure to Risk Factors)	Occupations or occupational tasks: comparative levels of incidence or prevalence	Other contextual or risk factors	Study Quality Scores
Krauss et al. 2017 [75]<br><br>*Country of origin: United States of America*	Prospective cohort	U.S. Army female active-duty members (recruits – tracked through initial 6 months [183 days] of service), entering the U.S. Army between February 2005 and September 2006 (N=1900).<br><br>Weight-qualified and fit: n=1061<br><br>Weight-qualified but unfit: n=524<br><br>Excess body fat but fit: n=315<br><br>Excess body fat and unfit: n=0	Participants took the Assessment of Recruit Motivation and Strength (ARMS) Harvard Step Test upon entering service and were graded as fit (completed test) and unfit (failed test). Participants were also categorised as weight qualified or exceeds body fat according to weight-for-height standards set by the Army. Observation period was 183 days. Data sources were procured from the US Military Entrance Processing Command and Defense Manpower Data Center for participant demographics; and the Standard Ambulatory Data Record was queried using ICD-9 codes relating to stress fracture (i.e. 733.93-.95) to identify cases.	Overall stress fracture *case* incidence rate in the female cohort: 196 cases in 132,400 person-days, equating to 1.48 cases per 1,000 person-days, *or* 540 cases (individuals with one or more stress fractures) per 1,000 person-years	**Stress fracture *case* incidence rates (cases per 1,000 person-days) and adjusted incidence rate ratios and 95% CI in the categorised female soldiers**<br>**Weight-qualified participants** | **Case incidence rate** | **Adjusted IRR***<br>Fit | 1.31 | 1.00 (Reference)<br>Unfit | 2.19 | 1.62 (1.19-2.21)<br>**Adjusted for age category, smoking status, BMI, race, and education*<br>**Fit participants** | **Case incidence rate** | **Adjusted IRR***<br>Weight-qualified | 1.31 | 1.00 (Reference)<br>Excess body fat | 1.02 | 0.79 (0.49-1.28)<br>**Adjusted for age category, smoking status*	67%<br><br>Level of Evidence: II
Kucera et al. 2016 [76]<br><br>*Country of origin: United States of America*	Prospective cohort	Military academy cadets or midshipmen from USMA (Westpoint), USNA, and USAFA, totalling three classes from each between 2005 and 2008 (N = 9,811; *male* N = 7,390, age range = 16 – 23 years, mean age = 18.8 years; *female* N = 2,421, age range = 16 – 22, mean age was not available to be computed).	Lower extremity injuries, including fractures and stress fractures, occurring during two months of basic training (1 Jul-31 Aug) were identified via queries of the Defense Medical Surveillance System (DMSS) using ICD-9 codes.	**Frequencies and calculated incidence rates (stress fractures per 1,000 person-years) of lower extremity *stress fractures* among first-year cadets during 2 months of basic training**<br>**Location** | **Stress fracture (n; IR)**<br>High/thigh | 0; 0<br>Knee | 0; 0<br>Lower leg | 54; 33.02<br>Ankle | 0; 0<br>Ankle/foot | 53; 32.41<br>Foot/toes | 0; 0<br>Total (lower extremity sites) | 107; 65.44	**Adjusted risk ratios for lower leg stress fractures in cadets of each sex, by prior histories of lower leg injury and stress fracture among military cadets**<br>**Male Cadets**<br>**Lower Leg Stress fracture** | History of lower leg injury | History of lower leg injury with activity limitation<br>No **history** | 1.00 (ref) | 1.00 (ref)<br>Prior **history** | 3.58 (1.13-11.34) | 17.03 (4.73-61.29)<br>**Female cadets**<br>**Lower Leg Stress fracture** | History of lower leg injury | History of lower leg injury with activity limitation<br>No **history** | 1.00 (ref) | 1.00 (ref)<br>Prior **history** | 6.06 (3.02-12.14) | 9.68 (3.91-23.95)	78%<br><br>Level of Evidence: II

Study	Study Design	Participants	Methods (Diagnosis / Exposure to Risk Factors)	Occupations or occupational tasks: comparative levels of incidence or prevalence	Other contextual or risk factors	Study Quality Scores
					Adjusted for age, injury prevention program participation, number of sports, and distance running index	
Lappe et al. 2008 [28]<br><br>*Country of origin: United States of America*	Randomized controlled trial (RCT)	5,201 female U.S. naval recruits undertaking basic training were recruited to participate in the trial between May 2001 – March 2006.<br><br>3700 participants completed the trial.	Recruits randomly assigned to two groups. Intervention group received ingestion of 2000 mg calcium and 800 IU vitamin D. Control group received identical placebo.<br><br>Recruit stress fracture injuries were monitored throughout basic training (8-weeks) in the medical clinic. Diagnoses were confirmed with radiography or technetium scan.	*Case* incidence rate for stress fractures reported as 5.9% of recruits per 8 wk training period (309 participants diagnosed with stress fractures), equating to 383.5 cases with one or more stress fractures per 1,000 person-years of basic training.	*A lower proportion of the intervention group (calcium + vitamin D) (5.3%) than of the control group (6.6%) was diagnosed with stress fractures, with a RR of 0.80 (95% CI 0.64-0.99).<br>* *Inclusive of all enrolled participants, analysed by intention to treat*<br><br>Including only recruits who completed the study (*n* = 3700) in a per protocol analysis, 21% fewer recruits were diagnosed with stress fractures in the intervention group (6.8%) than in the control group (8.6%; $p = 0.02$)<br><br>**Statistically significant findings of analyses assessing relationships between stress fracture occurrence and specific risk factors, adjusting for treatment effect**<br>**Factor** – **Relative risk (RR)**<br>Amenorrhea vs normal menstruation – 1.83<br>Age > 25 vs younger – 1.60<br>Depomedroxyprogesterone use vs non-use – 1.45<br>Smoker vs never smoked – 1.41<br>Run time (<15 minutes/1.5 mi vs longer) – 0.78<br>Exercise history (≥3 times/wk vs less) – 0.70<br>Dairy food consumption, alcohol use, previous fractures, race/ethnicity, and family history of osteoporosis were not significantly associated with stress fracture occurrence<br>**Logistic regression analysis (all factors included in model, which was adjusted for treatment)**<br>**Factor*** – **Odds Ratio**	100%<br><br>Level of Evidence: II

Study	Study Design	Participants	Methods (Diagnosis / Exposure to Risk Factors)	Occupations or occupational tasks: comparative levels of incidence or prevalence	Other contextual or risk factors	Study Quality Scores
					Amenorrhea vs normal menstruation 1.86 (1.41-2.45)<br>Age > 25 vs younger 1.66 (1.07-2.45)<br>Depomedroxyprogesterone use vs non-use 1.24 (0.91-1.68)<br>Smoker vs never smoked 1.31 (0.99-1.75)<br>Run time (<15 min/1.5 mi vs longer) 0.81 (0.62-1.05)<br>Treatment vs placebo 0.78 (0.61-1.01)<br>Exercise history (≥3 times/wk vs less) 0.66 (0.51-0.86)<br>* *none of these covariates had statistically significant interactions with each other*	
Lappe et al. 2005 [77]<br><br>*Country of origin: United States of America*	Prospective cohort	Female U.S. Army recruits (N = 4139; median age = 19.63 years (range 16-35 yrs)) commencing basic training (duration of 8 weeks) between August 1995 – July 1996.	Recruits underwent quantitative ultrasound (QUS) as a proxy measure of bone mineral density (BMD) at the calcaneus bone (speed of sound and broadband ultrasound attenuation), prior to commencing basic training. Other measured variables included: dairy food consumption, previous fractures, family history of osteoporosis, current or past smoking, regular weight-bearing exercise, and use of corticosteroid medication and contraceptives. Stress fractures were diagnosed by medical personnel and confirmed with radiographs or Technetium-99m (^{99m}Tc) scan.	Overall calculated *stress fracture* incidence during basic training over the period of observation was 321 stress fractures in 4139 recruits across the 8 weeks of basic training, equating to 504.1 stress fractures per 1,000 person-years of basic training. Overall calculated *case* incidence of stress fractures during basic training over the period of observation was 194 cases in 4139 recruits across the 8 weeks of basic training, equating to 304.7 cases per 1,000 person-years of basic training.	**Relative risks (RR) of stress fracture occurrence, by exposure factors**<br><br>**Factor** / **RR, adjusted by race and speed of sound (SOS) from QUS**<br>Age (each +1 year) 1.11 (1.07-1.14)<br>**Factor** / **RR, adjusted by age and speed of sound (SOS) from QUS**<br>American Indian 0.51 (0.07-3.87)<br>Asian 1.73 (0.59-5.13)<br>Black 1.00 (reference)<br>Hispanic 2.28 (1.28-4.06)<br>White 2.11 (1.30-3.20)<br>**Factor** / **RR, adjusted by age, race, and speed of sound (SOS) from QUS**<br>Smoker (yes vs never) 1.22 (0.89-1.67)<br>> 10 alcoholic drinks/week vs less 3.08 (1.59-5.97)	78%<br><br>Level of Evidence: II

Study	Study Design	Participants	Methods (Diagnosis / Exposure to Risk Factors)	Occupations or occupational tasks: comparative levels of incidence or prevalence	Other contextual or risk factors	Study Quality Scores
					Exercise (history of ≥3 sessions/week vs less) 0.55 (0.41-0.74)<br>**RR of stress fracture occurrence associated with each single unit decrease in speed of sound (SOS) and broadband ultrasound attenuation (BUA) in QUS of the calcaneus (reductions in each indicate reduced BMD)**<br>RR (for each 1 m/s ↓ in SOS) was 1.09 (1.07-1.11) RR (for each 1 db/MHz ↓ in BUA) was 1.03 (1.02-1.04);	
Lappe, Stegman & Recker (2001) [78]<br><br>*Country of origin: United States of America*	Prospective cohort	Female U.S. Army recruits (N = 3758; mean ± SD age = 21.1 ± 3.7 years (range 16-35)) commencing basic training (duration of 8 weeks) at Fort Leonard Wood between August 1995 – June 1996.	Recruits underwent quantitative ultrasound (QUS) as a proxy measure of bone mineral density at the calcaneus bone (speed of sound and broadband ultrasound attenuation), prior to commencing basic training.<br>Other measured variables included: dairy food consumption, previous fractures, family history of osteoporosis, current or past smoking, regular weight-bearing exercise, and use of corticosteroid medication and contraceptives.<br><br>Stress fractures were diagnosed by medical personnel and confirmed with radiographs or Technetium-99m (^{99m}Tc) scan.	Overall calculated *stress fracture* incidence during basic training over the period of observation was 504 stress fractures in 3758 female recruits across the 8 weeks of basic training, equating to 871.7 stress fractures per 1,000 person-years of basic training.<br>Overall calculated *case* incidence rate for stress fractures during basic training over the period of observation was 84.9 cases per 1,000 female recruits (319 cases in total) across 8 weeks of basic training, equating to 551.8 cases per 1,000 person-years of basic training.	**Relative risk (RR) of stress fracture occurrence, by exposure factors**<br>**Factor** — **RR, adjusted by race and speed of sound (SOS) from QUS**<br>Age (each +1 year) — 1.07 (1.05-1.10)<br>**Factor** — **RR, adjusted by age and speed of sound (SOS) from QUS**<br>White — 1.18 (1.07-1.31)<br>Black — 1.00 (reference)<br>All other races — 1.50 (1.003-2.25)<br>**Factor** — **RR, adjusted by age, race, and speed of sound (SOS) from QUS**<br>Lowest weight as adult (each -1 lb) — 1.01 (1.004-1.02)<br>Current smoker vs not — 1.25 (0.97-1.60)<br>History of smoking vs none — 1.34 (1.05-1.71)<br>Packs/day (each +1 pack) — 1.15 (0.94-1.41)	78%<br><br>Level of Evidence: II

<table>
<tr><th>Study</th><th>Study Design</th><th>Participants</th><th>Methods (Diagnosis / Exposure to Risk Factors)</th><th>Occupations or occupational tasks: comparative levels of incidence or prevalence</th><th>Other contextual or risk factors</th><th>Study Quality Scores</th></tr>
<tr><td></td><td></td><td></td><td></td><td></td><td><table>
<tr><td>Years smoked (each +1 year)</td><td>1.05 (1.02-1.08)</td></tr>
<tr><td>> 10 alcoholic drinks/week vs less</td><td>3.22 (1.82-5.69)</td></tr>
<tr><td>Exercise (history of ≥3 sessions/week vs less)</td><td>0.65 (0.51-0.82)</td></tr>
<tr><td>Years of exercise (each +1 year)</td><td>0.89 (0.84-0.95)</td></tr>
<tr><td>History of steroid use vs none</td><td>1.80 (0.86-3.79)</td></tr>
</table></td><td></td></tr>
<tr><td>Mattila et al. 2007 [79]

Country of origin: Finland</td><td>Prospective cohort</td><td>Finnish male and female conscripts completing military service between 1998-2004 (N = 152,095; females = 2,345, males = 149,750). Total exposure time = 102,515 person-years.</td><td>Stress fractures of the pelvis, hip, thigh or knee were identified using the MRI archives of the Central Military Hospital. Patients suspected of bone stress injuries were referred to the military hospital where diagnosis was confirmed via MRI and orthopaedic surgeon examination</td><td>Overall incidence rate of bone stress injuries of the pelvis, femur, tibial condyle or patella was 311 (95% CI, 277-345) bone stress injuries per 100,000 person-years, equating to 3.1 bone stress injuries per 1,000 person-years

Frequencies of bone stress injuries by body location and sex, in 149,750 males and 2,345 females, with RR comparing the sexes<table>
<tr><th>Body site</th><th>Male (n)</th><th>Female (n, RR female: male)</th></tr>
<tr><td>Sacrum</td><td>15</td><td>12, RR 51.1</td></tr>
<tr><td>Ramus</td><td>17</td><td>9, RR 33.8</td></tr>
<tr><td>Femoral neck</td><td>74</td><td>14, RR 12.1</td></tr>
<tr><td>Femoral Shaft</td><td>8</td><td>0, -</td></tr>
<tr><td>Patella</td><td>4</td><td>0, -</td></tr>
<tr><td>Femoral condyle</td><td>85</td><td>1, RR 0.8</td></tr>
<tr><td>Tibial condyle</td><td>76</td><td>4, RR 3.4</td></tr>
<tr><td>All sites total</td><td>279</td><td>40, RR 33.8</td></tr>
</table></td><td>Hazard ratios (HR) for bone stress injuries, comparing categories within specific risk factors<table>
<tr><th>Factor</th><th>Category</th><th>Adjusted* HR (95% CI)</th></tr>
<tr><td>Sex</td><td>Male</td><td>1.0 (Reference)</td></tr>
<tr><td></td><td>Female</td><td>8.2 (4.8-14.2)</td></tr>
<tr><td>Serving time</td><td>180 d</td><td>1.0 (reference)</td></tr>
<tr><td></td><td>270 d</td><td>n/a</td></tr>
<tr><td></td><td>362 d</td><td>0.7 (0.5-0.9)</td></tr>
<tr><td>Age (yr)</td><td>17-19</td><td>1.0 (reference)</td></tr>
<tr><td></td><td>20</td><td>1.2 (0.9-1.7)</td></tr>
<tr><td></td><td>21-29</td><td>2.1 (1.4-3.1)</td></tr>
<tr><td>12-min running test</td><td>Highest quartile</td><td>1.0 (reference)</td></tr>
<tr><td></td><td>Third quartile</td><td>1.0 (0.7-1.6)</td></tr>
</table></td><td>89%

Level of Evidence: II</td></tr>
</table>

Study	Study Design	Participants	Methods (Diagnosis / Exposure to Risk Factors)	Occupations or occupational tasks: comparative levels of incidence or prevalence	Other contextual or risk factors	Study Quality Scores
					Second quartile 1.3 (0.8-2.0)<br>Lowest quartile 1.3 (0.8-2.0)<br>**Muscle strength** Highest quartile 1.0 (reference)<br>Third quartile 0.8 (0.5-1.4)<br>Second quartile 1.2 (0.8-1.9)<br>Lowest quartile 1.4 (0.7-1.9)<br>**Body mass index** < 20 kg•m^{-2} 1.0 (reference)<br>20-25 kg•m^{-2} 0.7 (0.5-1.0)<br>>25 kg•m^{-2} 0.7 (0.5-1.1)<br>**Height** Shortest quartile 1.0 (reference)<br>Second quartile 1.0 (0.8-1.6)<br>Third quartile 1.1 (0.7-1.6)<br>Tallest quartile 1.2 (0.6-1.5)<br>**adjusted for sex, serving time, age, 12-min running test result, muscle strength, body mass index, height*	
Merkel et al. 2008 [26]<br><br>*Country of origin: Israel*	Prospective cohort	Three companies of Karakal (light infantry) Israel Defense Forces male (n = 83) and female (n = 227) combatant recruits (age range = 18-20 yrs) were followed.<br>Comparator group of non-combatants were also followed (non-combatant females n = 128).	Haematological and inflammatory blood marker samples were collected at induction, 2 months, and 4 months for female and male combatants and non-combatant comparator group.<br><br>Stress fractures were diagnosed by either radiography or scintigraphy.	Calculated overall *case* incidence rate for stress fractures in female combatants undergoing Karakal basic training was 118.9 cases per 1,000 recruits over the 20-week observation period, equating to 309 cases per 1,000 person-years in the female cohort.<br>No stress fractures occurred in either the non-combatant group, or the male combatants	**Iron and Transferrin levels in female combatants with (SF) and without stress fractures (NSF) at key time-points**<br><br>**Iron (µg•dL^{-1}) (mean ± SD)**<br>**Time-point** / **Stress Fracture group (*n*)** / **Iron**<br>0-months / SF (27) / 54 ± 24<br>*(p = 0.02)* / NSF (198) / 70 ± 46<br>4-months / SF (23) / 56 ± 35<br>*(p = 0.004)* / NSF (126) / 81 ± 46<br>**Transferrin (mg•dL^{-1}) (mean ± SD)**	78%<br><br>Level of Evidence: II

Study	Study Design	Participants	Methods (Diagnosis / Exposure to Risk Factors)	Occupations or occupational tasks: comparative levels of incidence or prevalence	Other contextual or risk factors	Study Quality Scores
					**Time-point** / **Stress Fracture group (*n*)** / **Transferrin (higher levels are associated with iron deficiency)**<br>2-months / SF (22) / 355 ± 80<br>(*p = 0.04)* / NSF (164) / 316 ± 57<br>4-months / SF (23) / 370 ± 52<br>(*p = 0.01)* / NSF (126) / 336 ± 56<br><br>There were no significant associations between other blood markers (i.e. ferritin, C-reactive protein or interleukin-6) and the stress fracture groups.	
Milgrom et al. 2017 [47]<br><br>*Country of origin: Israel*	Retrospective cohort	Participants were recruits completing 14-weeks of basic training within the same Israel Defense Forces infantry unit in the years 1983, 1988, 2002, 2006, 2007, 2011, 2012, 2013, and 2015 (N = 544).	Stress fracture diagnosis was made by clinical examination and radiographs from orthopaedic surgeons.<br>Major training changes in the observation years are as follows:<br>A) Cumulative formal march distance was 548 km between 1983-1988; from 2002 this reduced to 348 km.<br>B) 1988 – training restricted to authorised training protocols<br>C) 2002 – geographical training area moved from hilly to flatter; cumulative march distance lowered<br>D) 2006 – suspected tibia stress fracture treated as clinical stress fracture (prior to radiographical confirmation, recruits were placed on rest based on symptoms alone)<br>E) 2011 – 7 hour/day sleep regimen; physical therapist part of medical team; military boot replaced with hot weather U.S. boot	**Stress fracture cases (% of recruits; cases per 1,000 person-years) by year of observation**<br>**Year** / **Radiographic confirmation** / **Radiographic or clinical diagnoses**<br>1983 / 40%; 1486 / n/a<br>1988 / 20%; 743 / n/a<br>2002 / 15%; 557 / 15%; 557<br>2006 / 8%; 297 / 16%; 594<br>2007 / 5%; 186 / 15%; 557	**Multivariate analysis of risk factors associated with stress fracture occurrence**<br>**Factor (radiographically verified cases)** / **OR (95% CI)**<br>Weight (each 1kg decrease) / 1.05 (1.008-1.09)<br>Training not restricted vs training restricted to authorised protocols / 3.17 (1.10-9.09)<br>No early management of suspected stress fractures vs early management of potential stress fractures suspected based on clinical assessment, for 2 weeks prior to radiological confirmation / 2.38 (1.24-4.54)<br>**Factor (radiographically or clinically verified cases)** / **OR (95% CI)**<br>Weight (each 1kg decrease) / 1.03 (1.00-1.07)	78%<br><br>Level of Evidence: III-2

Study	Study Design	Participants	Methods (Diagnosis / Exposure to Risk Factors)	Occupations or occupational tasks: comparative levels of incidence or prevalence	Other contextual or risk factors	Study Quality Scores
			F) 2013 – specific running exercise halted, and lower body strength program added.	2011 12%; 446 14%; 520<br>2012 5%; 186 15%; 557<br>2013 11%; 409 13%; 483<br>2015 10%; 371 12%; 446	Training not restricted vs training restricted to authorised protocols 3.87 (1.52-9.83)<br>*Risk factors which demonstrated unclear, non-significant, effects in the multivariate model were moving training to flatter terrain, reducing formal march distance, sleep regimen, physical therapist in unit, new boots, reducing formal running and increasing lower body strengthening, age, height, BMI.*	
Milgrom et al. 2000 [17]<br><br>*Country of origin: Israel*	Prospective cohort	Participants comprised elite Israel Defense Forces infantry recruits from three cohorts training on the same base, but at different times.<br>1988 group: *n* = 452 infantry recruits (*n* completing training = 392; mean age 18.7 yrs (range, 17.9-25.2 yrs)).<br>1990 group: *n* = 433 infantry recruits (*n* completing training = 394; mean age 18.8 yrs (range, 17.7-24.8 yrs)).<br>1995 group: *n* = 404 infantry recruits (*n* completing training = 332; mean age 18.8 yrs (range, 17.7-27.3 yrs)).	Stress fractures were diagnosed at the central army stress fracture clinic using bone scintigraphy.<br><br>Sporting activity history was evaluated at induction through an oral questionnaire, with answers recorded.	**Calculated overall *case* incidence rates for stress fractures (cases per 1,000 recruit-years) for each of the induction groups during the 14-week basic training period**<br>**Group** / **Case incidence rate**<br>1988 881.0<br>1990 914.5<br>1995 604.3<br>Overall 810.5	**Calculated stress fracture *case* incidence rates (cases per 1,000 recruit-years) by body location and sports history, over the 14-week basic training period**<br>**Sports history** / **Tibia stress fracture** / **Femoral stress fracture**<br>Ball sports played 257.4 148.9<br>No ball sports played 726.1 338.7<br><br>**Calculated stress fracture *case* incidence rates (cases per 1,000 recruit-years) in each induction group, stratified by sports history and body locations, over the 14-week basic training period**<br>**1988 intake group: Ball sports played**<br>**All stress fractures** / **Tibia stress fracture** / **Femoral stress fracture**<br>489.5 172.7 287.9<br><br>**1988 intake group: No ball sports played**<br>**All stress fractures** / **Tibia stress fracture** / **Femoral stress fracture**<br>1073.4 861.3 395.6<br>**1990 intake group: Ball sports played**	78%<br><br>Level of Evidence: II

Study	Study Design	Participants	Methods (Diagnosis / Exposure to Risk Factors)	Occupations or occupational tasks: comparative levels of incidence or prevalence	Other contextual or risk factors	Study Quality Scores
					**All stress fractures** / **Tibia stress fracture** / **Femoral stress fracture**<br>619.2 / 453.9 / 41.2<br>**1990 intake group: No ball sports played**<br>**All stress fractures** / **Tibia stress fracture** / **Femoral stress fracture**<br>1001.7 / 769.6 / 256.7<br>**1995 intake group: Ball sports played**<br>**All stress fractures** / **Tibia stress fracture** / **Femoral stress fracture**<br>135.2 / 135.2 / 0.0<br>**1995 intake group: No ball sports played**<br>**All stress fractures** / **Tibia stress fracture** / **Femoral stress fracture**<br>697.2 / 549.7 / 375.5<br><br>**Odds ratios for stress fracture occurrence in recruits with history of ball sports, compared to no ball sports**<br>1988 OR = 0.373 (95% CI 0.21-0.66)<br>1990 OR = 0.541 (95% CI 0.29-0.99)<br>1995 OR = 0.16 (95% CI 0.03-0.69)	
Montain et al. 2013 [48]<br><br>*Country of origin: United States of America*	Retrospective cohort	All U.S. Army Basic Combat Training (BCT) trainees from Jan 1997 – Jan 2007 (*n* = 421,461 men; 90,141 women).	The Armed Forces Health Surveillance Center (AFHSC) data repository was utilised to extract personnel demographics, characteristics, and medical encounters. Medical encounters were queried using ICD-9 codes relating to stress fractures (733.1-.19, 733.93-.99), lower leg fractures (820-829) and frank fractures (800-829).	Overall *case* incidence for stress fractures during army basic combat training over the period of observation was 29.5 cases per 1,000 person-years	**Case incidence rates (cases per 1,000 person-years) and odds ratio (OR) comparing stress fracture risk in men and women trainees**<br>**Men** / **Women** / **OR women/men**<br>18.8 / 79.6 / 4.51 (95% CI 4.36-4.66)<br><br>Overall odds ratio comparing risk of sustaining a stress fracture in personnel from a low UV index group and a high UV index group: 0.83 (95% CI 0.80-0.87)	78%<br><br>Level of Evidence: III-2

Study	Study Design	Participants	Methods (Diagnosis / Exposure to Risk Factors)	Occupations or occupational tasks: comparative levels of incidence or prevalence	Other contextual or risk factors	Study Quality Scores
			Outcome measures of interest were personnel primary Home of Residence (HOR) and their exposure to ultraviolet (UV) light, and relationship to stress fractures.		**Odds ratios (OR) comparing risks of sustaining stress fracture during BCT by HOR UV level or Race/Ethnicity category, stratified by sex**<br><br>UV Index / Men (OR [95% CI]) / Women (OR [95% CI])<br>High: 1.00 (reference) / 1.00 (reference)<br>Medium: 1.07 (1.01-1.13) / 1.02 (0.97-1.08)<br>Low: 0.92 (0.87-0.97) / 0.89 (0.84-0.95)<br><br>Race/ Ethnicity / Men (OR [95% CI])) / Women (OR [95% CI]))<br>White: 1.79 (1.66-1.93) / 1.60 (1.51-1.70)<br>Black: 1.00 (reference) / 1.00 (reference)<br>Hispanic: 1.61 (1.46-1.78) / 1.35 (1.24-1.47)<br>Asian: 1.44 (1.24-1.68) / 1.30 (1.12-1.50)<br>Am. Indian: 1.72 (1.37-2.15) / 1.31 (1.08-1.59)<br>Other: 2.59 (1.84-3.66) / 1.74 (1.21-2.50)<br>Unknown: 1.11 (0.81-1.53) / 1.05 (0.78-1.40)<br><br>**Odds ratios (OR [95% CI])) comparing risks of sustaining stress fracture during BCT by HOR level, stratified by Race/Ethnicity category and sex**<br><br>Race / Ethnicity / UV Index / Men (OR, 95% CI)<br>White: High / 1.00 (reference)<br>Medium / 1.00 (0.93-1.06)	

Study	Study Design	Participants	Methods (Diagnosis / Exposure to Risk Factors)	Occupations or occupational tasks: comparative levels of incidence or prevalence	Other contextual or risk factors			Study Quality Scores
						Low	0.79 (0.74-0.84)	
						High	1.00 (reference)	
					Black	Medium	1.12 (0.94-1.32)	
						Low	1.09 (0.90-1.33)	
						High	1.00 (reference)	
					Hispanic	Medium	1.35 (1.15-1.60)	
						Low	1.18 (0.99-1.39)	
						High	1.00 (reference)	
					Asian	Medium	1.10 (0.80-1.52)	
						Low	1.39 (1.01-1.90)	
						High	1.00 (reference)	
					Am. Indian	Medium	1.03 (0.62-1.71)	
						Low	0.80 (0.46-1.37)	
					**Race / Ethnicity**	**UV Index**	**Women (OR, 95% CI)**	
						High	1.00 (reference)	
					White	Medium	0.94 (0.87-1.01)	
						Low	0.74 (0.68-0.80)	
					Black	High	1.00 (reference)	

Study	Study Design	Participants	Methods (Diagnosis / Exposure to Risk Factors)	Occupations or occupational tasks: comparative levels of incidence or prevalence	Other contextual or risk factors	Study Quality Scores
					Medium 1.12 (1.00-1.25)<br>Low 1.05 (0.91-1.21)<br>High 1.00 (reference)<br>Hispanic Medium 1.11 (0.93-1.31)<br>Low 1.09 (0.92-1.29)<br>High 1.00 (reference)<br>Asian Medium 0.85 (0.62-1.16)<br>Low 0.57 (0.39-0.84)<br>High 1.00 (reference)<br>Am. Indian Medium 1.32 (0.86-2.04)<br>Low 0.71 (0.43-1.16)	
Moran et al. 2013 [80]<br><br>*Country of origin: Israel*	Prospective cohort	Israeli male recruits completing army basic and advanced training over a 6-month training period (training weeks 3-13 were evaluated with pedometers) (N = 44)	Data relating to anthropometrics and aerobic fitness were collected at baseline and at the end of advanced basic training (~4 months following baseline). Data relating to psychological factors were collected via questionnaire at baseline and after 2 months of training.<br>Pedometers were worn by recruits for weeks 3-13.<br>Stress fracture surveillance spanned the entire 6-month period (4-months basic training; 2 months advanced training) and stress fractures were diagnosed by two orthopaedic surgeons with	Overall calculated *case* incidence rate for radiologically confirmed stress fractures over the entire training period (6-months) was 363.6 cases per 1,000 recruits, equating to 727.2 cases per 1,000 person-years of army basic and advanced training.	**Statistically significant differences in means** (±SD) **for risk factors in recruits who sustained stress fracture when compared to those who did not**<br><br>**Risk Factor** / **Stress fracture** / **Non-stress fracture**<br>Avg. daily steps during training wks 3-13: 16,276 ± 3,317 / 14,103 ± 2,302<br>Total distance covered during: 866 ± 136 km / 744 ± 161 km	56%<br><br>Level of Evidence: II

Study	Study Design	Participants	Methods (Diagnosis / Exposure to Risk Factors)	Occupations or occupational tasks: comparative levels of incidence or prevalence	Other contextual or risk factors	Study Quality Scores
			only stress fractures with radiographic imaging confirmation considered in incidence calculations.		training wks 3-13<br>Perceived coping (scale 0-7) 5.87 ± 0.52 5.32 ± 0.99<br>Perceived extent to which Commander valued performance (scale 0-7) 6.14 ± 0.38 5.30 ± 0.62<br>Perceived motivation (scale 0-5) 3.75 ± 0.29 3.48 ± 0.60<br>Mean values for baseline demographic, anthropometric and fitness factors did not differ significantly between recruits who sustained or did not sustain a stress fracture	
Moran et al. 2012 [81]<br><br>*Country of origin: Israel*	Prospective cohort	Israeli male army recruits participating in basic and advanced training for an elite combat unit, from two different companies.<br>Company one (prediction model) consisted of *n* = 57 soldiers;<br>Company two (validation model) consisted of *n* = 59 soldiers.	Upon induction, data relating to anthropometrics, activity history, fitness, bone quality (computed tomography), nutrition, haematology profile, and psychological factors were collected.<br>Stress fracture injury surveillance spanned the 1-year training period. Stress fractures were diagnosed by two orthopaedic surgeons using bone scintigraphy or magnetic resonance imaging.	The overall calculated stress fracture incidence rate across both companies of recruits was 517.2 cases per 1,000 person-years<br><br>The overall calculated *case* incidence rate for stress fractures was 387.9 cases per 1,000 person-years	**Factors included in the final prediction model for stress fracture occurrence**<br><br>**Factor** — **Adjusted odds ratio indicating change in stress fracture risk per unit increase in each factor**<br>Aerobic training history (times/week) — 0.22 (0.82-0.59)<br>Aerobic training history (min/session) — 1.66 (1.02-2.69)<br>Waist circumference (cm) — 0.85 (0.71-1.01)<br>Prediction model successfully predicted presence or absence of stress fracture in 85.3% of recruits	67%<br><br>Level of Evidence: II

Study	Study Design	Participants	Methods (Diagnosis / Exposure to Risk Factors)	Occupations or occupational tasks: comparative levels of incidence or prevalence	Other contextual or risk factors	Study Quality Scores
					in the first company; and 76.5% of recruits in the second company.	
Moran et al. 2008 [82]<br><br>*Country of origin: Israel*	Prospective cohort	Three companies of a gender integrated Israeli army combat unit were recruited on their induction (*n* = 219 female soldiers; *n* = 83 male soldiers).	Upon induction, data relating to anthropometric variables, blood samples for hematology profile and markers of bone metabolism, fitness tests, bone quality (peripheral quantitative computed tomography), nutritional and activity habits, and psychological profile were collected.<br>Stress fracture injury surveillance spanned the 4-month basic training period. Stress fractures were diagnosed by two orthopaedic surgeons using bone scintigraphy or magnetic resonance imaging.	The calculated *case* incidence rate for stress fractures in female recruits was 369.9 cases per 1,000 person-years<br><br>There were no stress fractures recorded in the male recruits	**Final multivariate stress fracture prediction model for female army recruits**<br><br>**Factor** — **Adjusted odds ratio indicating change in stress fracture risk per unit increase in each factor**<br>Ferritin ($ng \cdot mL^{-1}$) — 1.03 (1.005-1.067)<br>BMI ($kg \cdot m^{-2}$) — 0.90 (0.78-1.03)<br>Fe ($\mu g \cdot dL^{-1}$) — 0.98 (0.97-1.0)<br>Burnout (scale 1-7) — 1.59 (1.04-2.41)<br>Height (cm) — 1.08 (1.01-1.15)<br>Prediction model successfully predicted presence or absence of stress fracture in 76.5% of recruits.	67%<br><br>Level of Evidence: II
Oetting et al. 2017 [49]<br><br>*Country of origin: United States of America*	Retrospective cohort	U.S. Army trainees in the fiscal year 2010 (N = 15,082; *n* = 13,145 male and *n* = 1,937 females)	Recruit responses at entry to the military on The Tailored Adaptive Personality Assessment System (TAPAS) were extracted from the U.S. Army Research Institute for Social and Behavioural Sciences.<br><br>Stress fracture diagnosis was made using ICD-9 codes (733.93-.95) in outpatient clinics within the military training environment.<br><br>The initial 180-days of service were evaluated.	Overall *case* incidence for stress fractures observed in the U.S. Army trainees in the first 180-days of service was 14.4 cases per 1,000 recruits, equating to 29.2 cases per 1,000 person-years	Overall *case* incidence for stress fractures observed in male U.S. Army trainees in the first 180-days of service was 12.6 cases per 1,000 recruits, equating to 25.6 cases per 1,000 person-years<br>Overall *case* incidence for stress fractures observed in female U.S. Army trainees in the first 180-days of service was 26.8 cases per 1,000 recruits, equating to 54.3 cases per 1,000 person-years<br>Stress fracture case incidence rate ratio (female: male): 2.14 (95% CI 1.57 to 2.91)<br><br>**Adjusted odds ratios indicating change in stress fracture risk per unit increase in TAPAS physical conditioning score, stratified by sex**<br>**TAPAS Item** — **Men[a]** — **Women[b]**<br>Physical conditioning* — 0.68 (0.57-0.80) — 0.60 (0.43-0.82)<br>*surrogate for self-reported activity scale, assessed as a z-score ranging from -2.33 to 2.33	89%<br><br>Level of Evidence: III-2

Study	Study Design	Participants	Methods (Diagnosis / Exposure to Risk Factors)	Occupations or occupational tasks: comparative levels of incidence or prevalence	Other contextual or risk factors	Study Quality Scores
					**a** covariates included age, BMI, physical demand rating for military occupational specialty<br>**b** covariates included age	
Orr et al. 2020 [50]<br><br>*Country of origin: Australia*	Retrospective cohort	Two cohorts of Australian Army recruits, from 28-day reservist training courses and 80-day basic recruit training courses, completing training 2006 – 2011.<br><br>Final analysis included N = 19,769 recruits (28-day course, n= 7692, 87.0% men; 80-day course, n= 12,077, 91.8% men). Mean ± SD recruit age was 22.2 ± 6.10 years.	Stress fracture cases in the two recruit cohorts were identified from the injury surveillance database of the Defence Injury Prevention Program (DIPP).<br><br>Predictor variables for the logistic regression model included participant demographics (e.g., height) and scores from physical fitness assessments (e.g., aerobic and muscular endurance).	Only one stress fracture was recorded in the 28-day army reservist course<br><br>The overall incidence of stress fractures in the 80-day standard course was 0.4%, equating to 17 stress fractures per 1,000 person-years	Stress fracture incidence in females completing the 80-day standard course was 1.3%, equating to 59.3 stress fractures per 1,000 person-years<br>Stress fracture incidence in males completing the 80-day standard course was 0.3%, equating to 13.7 stress fractures per 1,000 person-years<br>Stress fracture incidence rate ratio (female: male): 4.41 (95% CI 2.33 to 8.35)<br><br>**Summary of final prediction model for stress fractures in both the 28 and 80-day courses combined**<br><br>**Retained predictors:** Height (cm), initial shuttle run level, course no (directions of associations of each of these with stress fracture risk not specified).<br>**Nagelkerke R^2:** 0.10<br>**Area under the curve (AUC):** 0.78 (indicating *'fair' model performance)*	89%<br><br>Level of Evidence: III-2
Palmanovich et al. 2017 [91]<br><br>*Country of origin: Israel*	Randomised controlled trial (RCT)	Female Border Infantry recruits (Israel) completing 4-month basic training between 2007 and 2009 (N = 240)	Recruits split into two subgroups: Group 1 (*n* = 101) completed 4months of basic training wearing standard special unit fighting vest (SUFV) made for men and women (weight = 1350 grams)<br>Group 2 (*n* = 139) completed 4months of basic training wearing the new fighting vest (NFV) made for women and available in 3 sizes (weight = 1950 grams).	Overall stress fracture *case* incidence rate for recruits wearing the SUFV over the 4-month basic training period was 128.7 cases per 1,000 recruits, equating to 386.1 cases per 1,000 person-years<br><br>Overall stress fracture *case* incidence rate for recruits wearing the NFV over the 4-month basic training period was 129.5 cases per 1,000 recruits, equating to 388.5 cases per 1,000 person-years		46%<br><br>Level of Evidence: II

<table>
<tr><th>Study</th><th>Study Design</th><th>Participants</th><th>Methods (Diagnosis / Exposure to Risk Factors)</th><th>Occupations or occupational tasks: comparative levels of incidence or prevalence</th><th>Other contextual or risk factors</th><th>Study Quality Scores</th></tr>
<tr><td></td><td></td><td></td><td>Bone scintigraphy was used by the Medical Service to diagnose stress fractures.</td><td></td><td></td><td></td></tr>
<tr><td>Piantanida et al. 2000 [51]

Country of origin: United States of America</td><td>Retrospective cohort</td><td>U.S. Marine Corps officer candidates completing basic training over 6-weeks in the year 1997 (N = 480)</td><td>Data were obtained from entry questionnaires, daily medical logs, and individual medical records.
Stress fractures were diagnosed with clinical evaluation and nuclear medicine bone scan.</td><td>Overall incidence rate of stress fractures over the 6-week period of observation was 7 stress fractures in 480 officer cadets, equating to 126.4 stress fractures per 1,000 person-years of Marine Corps officer basic training</td><td>Overall incidence rate of stress fractures for male marine corps officers over the 6-week period of observation was 0.05 stress fractures per 100 trainees per 1,000 hours of training

Overall incidence rate of stress fractures for female marine corps officers over the 6-week period of observation was 0.03 stress fractures per 100 trainees per 1,000 hours of training, giving a female: male incidence rate ratio for stress fractures of 0.6</td><td>78%

Level of Evidence: III-2</td></tr>
<tr><td>Pihlajamaki et al. 2019 [52]

Country of origin: Finland</td><td>Retrospective cohort</td><td>Finnish male conscripts (N = 4029) born in the years 1969, 1974, 1979, 1984, and 1989 completing basic training (8 weeks) and subsequent service</td><td>Stress fracture cases were identified from garrison health care centre records using ICD-9 or ICD-10 codes. Stress fractures were diagnosed using radiography, scintigraphy or magnetic resonance imaging scans.</td><td>Overall incidence of stress fractures during military service was 1.27 (95% CI, 0.92-1.70) stress fractures per 1,000 follow-up months, equating to 15.2 stress fractures per 1,000 person-years

Stress fractures primarily occurred during the initial 4-months of military service, with the cumulative incidence of stress fractures occurring plateauing after this point, up to 12-months.</td><td>Regression model for risk of stress fractures (significant explanatory variables)

Self-reported pre-military service high levels of physical activity (i.e. more than twice a week, causing sweating and breathlessness) reduced stress fracture risk when compared to not having that level of physical activity prior to training commencement: incidence rate ratio 0.41 (95% CI 0.20-0.85)

Anatomical locations of stress fractures
<table><tr><th>Location</th><th>N</th><th>Percent of total</th></tr><tr><td>Metatarsal bones</td><td>17</td><td>43%</td></tr><tr><td>Tibia</td><td>16</td><td>40%</td></tr><tr><td>Calcaneus</td><td>2</td><td>5%</td></tr><tr><td>Femoral shaft</td><td>2</td><td>5%</td></tr><tr><td>Upper part of femur</td><td>2</td><td>5%</td></tr><tr><td>Femoral neck</td><td>1</td><td>3%</td></tr><tr><td>Upper part of tibia</td><td>1</td><td>3%</td></tr></table></td><td>89%

Level of Evidence: III-2</td></tr>
</table>

Study	Study Design	Participants	Methods (Diagnosis / Exposure to Risk Factors)	Occupations or occupational tasks: comparative levels of incidence or prevalence	Other contextual or risk factors	Study Quality Scores
					Both femurs, upper 1 3%<br>Femur & calcaneus 1 3%<br>Tibia & metatarsal bone 1 3%	
Pihlajamaki et al. 2006a [53]<br><br>*Country of origin: Finland*	Retrospective cohort	Finnish male military conscripts from 1/01/1970 – 31/12/1990 (total person-years evaluated was 546,317)	Conscripts treated for undisplaced femoral neck stress fractures were identified through an ICD-8 or ICD-9 query of the Finnish National Hospital Discharge Register.<br>Fractures were diagnosed using conventional anteroposterior (AP) and frog-leg lateral radiographs.	Overall incidence of undisplaced femoral neck stress fractures was 0.201 such stress fractures per 1,000 service-years.<br>The *case* incidence rate for undisplaced femoral neck stress fractures was 0.194 cases (individuals with one or more such stress fractures) per 1,000 service-years.<br><br>Incidence of undisplaced femoral neck stress fractures from 1970-1986 was 0.132 such stress fractures per 1,000 service-years.<br><br>Incidence of undisplaced femoral neck stress fractures from 1987-1990 was 0.532 such stress fractures per 1,000 service-years.	No evaluated potential risk factors (i.e. BMI, neck-shaft angle, or leg dominance) were significantly related to undisplaced femoral neck stress fracture occurrence	89%<br><br>Level of Evidence: III-2
Pihlajamaki et al. 2006b [54]<br><br>*Country of origin: Finland*	Retrospective cohort	Finnish male military conscripts (N = 694,457) from 1/01/1975 to 31/12/1994 (total person-years evaluated was 491,160)	Displaced femoral neck stress fracture diagnoses were identified using ICD-8 and ICD-9 codes and extracted from the National Hospital Discharge Register.<br><br>Note: Changes were implemented to the diagnosis and prevention of displaced femoral neck stress fractures from the year 1986.	Overall incidence of displaced femoral neck stress fractures was 0.043 such stress fractures per 1,000 service-years<br>Incidence of displaced femoral neck stress fractures from 1975-1986 was 0.053 per 1,000 service years<br>Incidence of displaced femoral neck stress fractures from 1987-1994 was 0.023 per 1,000 service years<br>Total overall incidence of femoral neck stress fractures (displaced and undisplaced) was 0.208 per 1,000 service-years from 1975-1986; and 0.532 per 1,000 service-years from 1987-1994 (none were displaced in the latter period 1987-1994)		89%<br><br>Level of Evidence: III-2
Popovich et al. 2000 [93]<br><br>*Country of origin:*	Quasi-experimental	Six companies of U.S. Army Training recruits undergoing basic training (8 weeks) from July, August and	Medical records were reviewed from the William Beaumont Army Medical Center utilising customised forms designed for the study to identify injuries and reports of bone scans and x-rays.	Overall incidence of stress fractures in the U.S. Army recruits undergoing basic training was 47.9 stress fractures per 1,000 recruits over the 8-week period of observation, equating to 311.4 stress fractures per 1,000 person-years.	**Stress fracture case incidence rate by company (stress fracture as primary diagnosis)**	89%<br><br>Level of Evidence: III-2

Study	Study Design	Participants	Methods (Diagnosis / Exposure to Risk Factors)	Occupations or occupational tasks: comparative levels of incidence or prevalence	Other contextual or risk factors	Study Quality Scores
United States of America		September 1989 (N = 1357).	Company interventions were as follows: C1 and C2 were standard controls, R2, R3, and R4 were training companies which utilised rest from running during week 2, 3, or 4, respectively. R5 increased running volumes; however, withdrew from running during week 4 and did not resume until week 6 due to an early impression of higher injury rates.	All stress fractures were recorded in the lower extremities; primarily in the foot (53%) and shin (28%).	**Company** / **Stress fracture case incidence rate (% of recruits affected by stress fractures within the 8-week training period)**<br>C1 (control): 3.6% (n = 9)<br>C2 (control): 1.0% (n = 2)<br>R2 (rest from running in week 2): 6.1% (n = 13)<br>R3 (rest from running in week 3): 1.9% (n = 5)<br>R4 (rest from running in week 4): 4.5% (n = 9)<br>R5 (↑ running volume): 1.8% (n = 4)<br><br>The interactions between external training load variables and stress fracture injury rates were unclear.	
Rauh et al. 2006 [13]<br><br>*Country of origin: Israel*	Prospective cohort	Female Marine Corps recruits completing basic training at Parris Island Marine Corps Recruit Depot (MCRD) in 1999 (N = 824, age range 17-31 years).	Stress fractures diagnosed via clinical examination and confirmed with radiograph and/or bone scan.<br>Total training day exposures (TDE): 68,686 TDE.	Overall incidence rate of stress fractures in female Marine Corps recruits in 1999 was 1.0 stress fracture per 1,000 training day exposures (TDE), equating to 365.0 stress fractures per 1,000 person-years<br><br>Incidence rate of *initial* stress fractures in female Marine Corps recruits in 1999 was 0.86 stress fractures per 1,000 TDE (i.e. up to the point of initial injury);<br><br>*Subsequent* rate of stress fractures was 3.0 stress fractures per 1,000 TDE (i.e. only TDE following initial injuries counted as the denominator);<br><br>The incidence rate ratio comparing subsequent stress fracture risk (following an initial stress fracture) to initial stress fracture risk was 3.5 (95% CI 1.6-6.7)<br><br>**Lower extremity stress fracture incidence rates (stress fractures per 1,000 person years), by anatomical location**<br>**Location** / **N** / **Rate**<br>Hip/pelvis / 10 / 54.8	**Unadjusted odds ratios (OR) for factors significantly associated with stress fractures in female recruits**<br>**Factor** / **Category** / **OR (95% CI)**<br>Run time / Quartile 1 (fastest) / 1.0 (reference)<br>Run time / Quartile 2 / 1.3 (0.5-3.2)<br>Run time / Quartile 3 / 1.2 (0.5-3.0)<br>Run time / Quartile 4 / 2.6 (1.2-5.8)<br>Self-rated fitness / Excellent-very good / 1.0 (reference)<br>Self-rated fitness / Good / 2.5 (0.7-8.6)	89%<br><br>Level of Evidence: II

Study	Study Design	Participants	Methods (Diagnosis / Exposure to Risk Factors)	Occupations or occupational tasks: comparative levels of incidence or prevalence			Other contextual or risk factors			Study Quality Scores
				Femur	7	36.5		Fair-poor	5.1 (1.5-17.1)	
				Tibia	38	200.8	Mean run distance (miles per day in 2 months prior to training)	≥ 3	1.0 (reference)	
				Fibula	4	21.9		1.5 – 2.9	2.9 (0.9-9.6)	
				Metatarsals	7	36.5		< 1.5	3.9 (1.1-13.5)	
							Months of LE weight training, past year	≥ 7	1.0 (reference)	
								0 – 6	5.4 (1.3-22.3)	
							Frequency of LE stretching per week (2 months prior to training)	≥ 2	1.0 (reference)	
								0 – 1	2.1 (1.2-3.7)	
							Secondary amenorrhea	No	1.0 (ref)	
								Yes	2.7 (1.1-6.9)	
							Exercise: times per wk (2 months prior to training)	5-7	1.0 (ref)	
								2-4	1.4 (0.7-2.7)	
								0-1	2.7 (1.2-6.0)	
							**Adjusted* OR for factors significantly associated with stress fractures in female recruits, from multivariate logistic regression model**			
							**Factor**	**Category**	**aOR (95% CI)**	
							Run time	Quartile 1 (fastest)	1.0 (reference)	
								Quartile 2	1.4 (0.5-3.9)	
								Quartile 3	1.1 (0.4-3.2)	
								Quartile 4	3.3 (1.4-8.1)	

Study	Study Design	Participants	Methods (Diagnosis / Exposure to Risk Factors)	Occupations or occupational tasks: comparative levels of incidence or prevalence	Other contextual or risk factors	Study Quality Scores
					Secondary amenorrhea: No — 1.0 (reference); Yes — 4.1 (1.5-10.9)<br>Months of LE weight training in past year: ≥ 7 — 1.0 (reference); 0 – 6 — 4.5 (1.1-18.9)<br>**Adjusted for each displayed factor and age, race/ethnicity, and BMI*<br>*Factors with unclear effects: race/ethnicity, age, BMI, history of LE stress fracture/non-stress fracture, freq. of sweating, change in exercise, freq. of running, mean run time, months run (past year), age at menarche, no. of menses (past year), birth control hormone use, oligomenorrhea*	
Reis et al. 2007 [2]<br><br>*Country of origin: United States of America*	Prospective cohort	Male U.S. Marine Corps recruits completing 12-weeks of basic training between Feb to April 2003 (N = 2,137)	Stress fractures were diagnosed by Naval medical staff and confirmed via x-ray, triple-phase bone scan, or magnetic resonance imaging.<br><br>NOTE: Cases include bone stress reactions which were defined in this study as an early stress fracture, prior to disruption of the bone.	Total *case* incidence rate for stress fractures in male Marine Corp recruits completing basic training was 0.74 cases per 1,000 training day exposures, equating to 270.1 cases per 1,000 person-years	**Stress fracture occurrence during training as a risk factor for discharge from service in the Marine Corps**<br>**Stress fracture category** / **Adjusted odds ratio for discharge from the Marine Corps**<br>Yes — 4.19 (2.73-6.45)<br>No — 1.00 (reference)<br>** Adjusted for demographics, physical characteristics, self-reported exercise participation, and LE injuries prior to training*	78%<br><br>Level of Evidence: II
Ross & Allsopp (2002) [66]<br><br>*Country of origin: United Kingdom*	Retrospective cohort	U.K. Royal Marines Commandos male recruits completing basic training (N = 3,574) in either the:<br>A) Conventional (original) training curriculum (*n*=1483 at commencement; mean age 20.8 yrs)<br>B) New and modified training curriculum (*n*=2091 at commencement; mean age 19.5 yrs)	All lower limb stress fractures diagnosed with plain radiography or bone scan between 1997 and June 30th 2000 were examined.	The overall incidence rate for *lower limb* stress fractures in the UK Royal Marines Commando recruits during basic infantry training was estimated, based on numbers of stress fractures recorded, numbers of recruits who reached Commando testing weeks near the end of the program, and the 30-week training duration, to be:<br><br>3.89 stress fractures per 1,000 person-weeks (equating to 202.3 stress fractures per 1,000 person-years) across both training programs combined		78%<br><br>Level of Evidence: III-2

Study	Study Design	Participants	Methods (Diagnosis / Exposure to Risk Factors)	Occupations or occupational tasks: comparative levels of incidence or prevalence	Other contextual or risk factors	Study Quality Scores
				Within each program, the incidence rates were estimated, on the same basis, to be:<br><br>4.09 stress fractures per 1,000 person-weeks (equating to 212.8 stress fractures per 1,000 person-years) in the original program/curriculum<br><br>3.68 stress fractures per 1,000 person-weeks (equating to 191.2 stress fractures per 1,000 person-years) in the new program/curriculum<br><br>The new program involved increased aerobic power entry requirements, more active rest and more running earlier in the program, when compared to the original program		
Ruohola et al. 2006 [25]<br><br>*Country of origin: Finland*	Prospective cohort	Male Finnish infantry recruits undergoing military training (N = 756; mean age 19.8 years, range 18-28)	Stress fractures were diagnosed by medical professionals through clinical examination and radiographic imaging and/or MRI.<br><br>Vitamin D (serum 25(OH)D) samples were extracted upon entry to military training.<br><br>Total follow-up period of 3-months	Overall stress fracture *case* incidence rate was 116 (95% CI, 68-165) cases per 1,000 person-years	**Risk factors and associated adjusted odds ratios for stress fracture, based on multivariate logistic regression**<br>**Risk factor** / **Category** / **aOR (95% CI)**<br>25 (OH) D / Less than median (<75.8 nM) / 3.6 (1.2-11.1)<br>/ ≥ 75.8 nM / 1.0 (reference)<br>Cooper test/ 12-minute run / Continuous (aOR is per metre increase in distance run) / 0.999 (0.997-1.000)<br>*BMI, age, smoking status and muscle strength were not found to be significant risk factors*	89%<br><br>Level of Evidence: II
Ruohola et al. 2009 [84]<br><br>*Country of origin: Finland*	Prospective cohort	Finnish military conscripts undergoing training (N = 820; males = 796, females = 24; mean age 19.8 years, range 18-28)	Stress fractures were diagnosed by medical professionals through clinical examination and radiographic imaging and/or MRI.<br>Serum tartrate acid phosphatase isoform 5b (TRACP-5b) samples were taken at entry, on first day of stress injury, and 2nd 3rd and 4th samples were drawn at 3 to 4-day intervals (samples II, III, IV).<br>Total follow-up period of 3-months.	Total calculated *case* incidence rate for stress fractures observed within the 90-day follow-up period was 24.4 cases per 1,000 recruits, equating to 99.0 cases per 1,000 person-years	The relationship between TRACP-5b measures and stress fracture occurrence was non-significant	78%<br><br>Level of Evidence: II

Study	Study Design	Participants	Methods (Diagnosis / Exposure to Risk Factors)	Occupations or occupational tasks: comparative levels of incidence or prevalence	Other contextual or risk factors	Study Quality Scores
Salminen et al. 2003 [55]<br><br>*Country of origin: Finland*	Retrospective cohort	Finnish military recruits from 1/01/1980 – 31/12/1999 (average population at risk per year was 33,000 over the 20-year period; 18-19 years of age)	All medical records and radiographs of displaced stress fractures of the femoral shaft in Finnish military recruits treated at the National Military Hospital of Finland were identified and included as cases	Overall *case* incidence of displaced femoral shaft stress fractures was 1.5 cases per 100,000 person-years of military service, equating to 0.015 cases per 1,000 person-years<br>Each case involved one fracture and so the incidence of displaced femoral shaft stress fractures was also 0.015 such stress fractures per 1,000 person-years		67%<br><br>Level of Evidence: III-2
Schaffer et al. 2006 [85]<br><br>*Country of origin: United States of America*	Prospective cohort	Female Marine Corps recruits completing basic training from March 1995 – September 1996 (N = 2,962; 17-33 years).	Stress fractures were diagnosed with radiographs or scintigraphy by the senior medical officer at the Branch Medical Clinic.	Overall calculated incidence of stress fractures during 13-weeks of basic training was 61.1 stress fractures per 1,000 recruits, equating to 244.4 stress fractures per 1,000 person-years<br><br>**Distribution of stress fractures by body site**<br>Tibia 24.9% (IR 60.9 tibial stress fractures per 1,000 person-years)<br>Metatarsal 22.1% (IR 54.0 metatarsal stress fractures per 1,000 person-years)<br>Pelvis 21.6% (IR 52.8 pelvic stress fractures per 1,000 person-years)<br>Femur 19.9% (IR 48.6 femoral stress fractures per 1,000 person-years)<br>Ankle/foot 7.7% (IR 18.8 ankle/foot stress fractures per 1,000 person-years)<br>Fibula 3.9% (IR 9.5 fibular stress fractures per 1,000 person-years)	**Risk factors and associated adjusted* odds ratios for stress fractures, based on the multivariate logistic regression model**<br><br>**Risk factor / Category / aOR (95% CI)**<br>Run time: Quartile 1 (fastest) / 1.00 (reference)<br>Run time: Q2 / 1.21 (0.5-2.1)<br>Run time: Q3 / 3.41 (1.9-6.1)<br>Run time: Q4 (slowest) / 3.54 (2.0-6.3)<br>Number of periods of menses during past year: 10-12 / 1.00 (reference)<br>Number of periods of menses during past year: 1-9 / 0.76 (0.4-1.3)<br>Number of periods of menses during past year: 0 / 3.79 (1.3-10.7)	78%<br><br>Level of Evidence: II

Study	Study Design	Participants	Methods (Diagnosis / Exposure to Risk Factors)	Occupations or occupational tasks: comparative levels of incidence or prevalence	Other contextual or risk factors	Study Quality Scores
					**Risk factors and adjusted* odds ratios for *pelvic* or *femoral* stress fractures, based on the multivariate logistic regression model**<br>**Risk factor** / **Category** / **aOR (95% CI)**<br>Run time / Quartile 1 (fastest) / 1.00 (reference)<br>Q2 / 2.37 (0.9-6.2)<br>Q3 / 3.89 (1.6-9.6)<br>Q4 (slowest) / 3.14 (1.2-9.0)<br>Number of periods of menses during past year / 10-12 / 1.00 (reference)<br>1-9 / 1.12 (0.6-2.3)<br>0 / 5.85 (1.7-20.8)<br>** Adjusted for age, race/ethnicity, and for other factors included in the final model*	
Shapiro et al., 2022 [60]<br><br>Country of Origin: Israel	Retrospective Cohort	Male (n = 56072) and female (n = 6299) recruits enlisted to basic combat training (i.e., infantry, light infantry, combat engineering, armour, artillery, search and rescue warriors, air defence and field intelligence units) between 2014-2017.	Israel Defense Forces medical database was queried to identify records of stress fractures, diagnosed using radiographs and bone scans.	The period prevalence of clinically diagnosed stress fracture during the period 2014-2017 was 5.9%.		45%<br><br>Level of Evidence: III-2
Sharma et al. 2015 [12]<br><br>*Country of origin: United Kingdom*	Prospective cohort	British Army recruits (N = 6,608; mean ± SD age 18.9 ± 2.2 years; mean ± SD height 176.5 ± 7.8 cm; mean ± SD weight 69.2 ± 9.7 kg; mean ± SD BMI 22.1 ± 2.5	Stress fractures were diagnosed by clinical examination and x-ray and/or MRI scans.	Overall *case* incidence rate for stress fractures in British Army recruits completing the Combat Infantryman's Course in the two consecutive years was 31.3 cases per 1,000 recruits across the 26 weeks of training, equating to 62.6 cases per 1,000 person-years		78%<br><br>Level of Evidence: II

Study	Study Design	Participants	Methods (Diagnosis / Exposure to Risk Factors)	Occupations or occupational tasks: comparative levels of incidence or prevalence	Other contextual or risk factors	Study Quality Scores
		kg/m^2) completing the Combat Infantryman's Course (~26 weeks of training) from April 2006 – March 2008 (two-year period). Recruits were from the Line (66.2%), Guards (15.8%), Parachute (11.1%) and Gurkha (6.9%) regiments.		***Case* incidence rates for stress fractures (cases per 1,000 person-years) by anatomical location**<br>**Location** — **Incidence rate**<br>Tibia — 27.8<br>Metatarsal — 24.8<br>Femur — 5.2<br>Calcaneum — 4.8		
Sheehan et al. 2003 [94]<br><br>*Country of origin: United States of America*	Quasi-experimental	Marine Corps recruits completing 11-weeks of military training (*n* = 155 females, mean ± SD height 165.4 ± 6.5 cm, mean ± SD weight 60.1 ± 7.1 kg; *n* = 58 males, mean ± SD height 175.9 ± 6.3 cm, mean ± SD weight 69.4 ± 9.7 kg)	Stress fractures were diagnosed by clinical examination and x-ray findings.<br><br>Deoxypyridinoline (DPD; a biomarker of bone resorption) and creatinine (CR) were assessed at baseline (3 consecutive days) and every 3rd morning throughout the training period.	Overall *case* incidence rate for stress fractures in participating male recruits completing the 11-weeks of military training was 17.2 cases per 1,000 recruits, equating to 81.3 cases per 1,000 person-years<br><br>Overall *case* incidence rate for stress fractures in participating female recruits completing the 11-weeks of military training was 38.7 cases per 1,000 recruits, equating to 182.9 cases per 1,000 person-years	Calculated incidence rate ratio (female:male): 2.25 (95% CI 0.28-18.3)<br><br>DPD levels were not significantly associated with stress fracture occurrence in male or female Marine Corps recruits	89%<br><br>Level of Evidence: III-2
Sormaala et al. 2006 [56]<br><br>*Country of origin: Finland*	Retrospective cohort	Finnish military recruits completing military service between 1/04/1997 and 31/03/2005 (*total person-years of exposure* = 117,149)	Military recruits with MRI scan of talus bone stress injury (grade IV – fracture injury) from archives of Central Military Hospital	Incidence of talus bone stress fracture (grade IV stress injury) across the 96-month period of observation was 0.09 talar stress fractures per 1,000 person-years	There were no significant associations between talus bone stress fracture (Grade IV) occurrence and age, sex, length of service, aerobic fitness level or muscular strength	89%<br><br>Level of Evidence: III-2
Välimäki et al. 2005 [86]<br><br>*Country of origin: Finland*	Prospective cohort	Finnish male military recruits from an infantry unit (N = 179; ages 18-20 years) completing compulsory military service, ranging from 6 to 12 months in duration	Stress fracture diagnosed with clinical examination and X-ray findings.	Incidence rates were not calculable because durations of service periods were variable (ranging from 6 to 12 months) and total person-years of exposure were not reported for the cohort.<br><br>Overall frequency of stress fractures was 83.8 cases per 1,000 recruits<br><br>Frequency of metatarsal stress fractures was 55.9 cases per 1,000 recruits	**Factors significantly associated with stress fracture occurrence (vs. without)**<br>**Factor** — **Median factor values in those with vs without stress fracture** — ***p***<br>Height (cm) — 183 vs 180 — 0.04<br>Cooper test (distance m) — 2345 vs 2700 — 0.02	89%<br><br>Level of Evidence: II

Study	Study Design	Participants	Methods (Diagnosis / Exposure to Risk Factors)	Occupations or occupational tasks: comparative levels of incidence or prevalence	Other contextual or risk factors	Study Quality Scores
				Frequency of tibial stress fractures was 22.3 cases per 1,000 recruits<br><br>Frequency of calcaneal stress fractures was 5.6 cases per 1,000 recruits	Femoral neck bone mineral content (g) — 6.27 vs 6.52 — 0.02<br>Femoral neck bone mineral density (g/cm^2) — 1.14 vs 1.19 — 0.03<br>Total hip bone mineral content (g) — 41.6 vs 43.1 — 0.02<br>Total hip bone mineral density (g/cm^2) — 1.14 vs 1.19 — 0.04<br>Serum parathyroid hormone (ng/l) — 56 vs 35 — 0.02	
Wood et al. 2014 [58]<br><br>*Country of origin: United Kingdom*	Retrospective cohort (data prospectively collected)	U.K. Royal Marine recruits completing basic commando training (32 weeks) between April 2004 and April 2008 (N = 4200)	Stress fractures were diagnosed using plain radiographs and/or Magnetic Resonance Imaging (MRI), the latter in a small number of cases.	Overall incidence of stress fractures in Royal Marines completing 32 weeks of basic commando training was 52.4 stress fractures per 1,000 recruits, equating to 85.2 stress fractures per 1,000 person-years<br><br>**Incidence (stress fractures per 1,000 person-years) of stress fractures, by anatomical location**<br>**Location** — **Incidence**<br>Metatarsal — 55.3<br>Tibia — 20.2<br>Femur (femoral neck) — 8.1<br>Fibula — 2.3		78%<br><br>Level of Evidence: III-2
Wood & Kruger (2015) [95]<br><br>*Country of origin: South Africa*	Quasi-experimental (pre-post-test design)	South African male military recruits (N = 100) completing 12 weeks of basic military training	Stress fractures were diagnosed via MRI and radiographic findings.	There were no stress fractures recorded in this military recruit population (incidence rate = 0 stress fractures per 1,000 person-years)		89%<br><br>Level of Evidence: III-2
Yanovich et al. 2011 [27]<br><br>*Country of origin: Israel*	Prospective cohort	Israeli Defence Force military recruits completing basic training as part of mandatory service (n = 221 female [mean ± SD age 19.0 ± 0.9 years; mean ± SD	Stress fractures were diagnosed with clinical examination and confirmed using radiography, magnetic resonance imaging or bone scintigraphy.<br><br>There were two key time-points for data collection for	There were no observed stress fractures within the male combatant recruits or female non-combatant recruits<br><br>The *case* incidence rate for stress fractures in female combatant recruits was 66.0 cases per 1,000 recruits over the 14-weeks of basic training,	**Significant differences in frequencies of haematological factors between recruits with stress fractures (SF) and those with no stress fractures (NSF) (p<0.05 for each)**<br>**Month 0 - Baseline**<br>**Factor** — **SF (n = 14)** — **NSF (n = 149)**	89%<br><br>Level of Evidence: II

Study	Study Design	Participants	Methods (Diagnosis / Exposure to Risk Factors)	Occupations or occupational tasks: comparative levels of incidence or prevalence	Other contextual or risk factors	Study Quality Scores
		weight 60.6 ± 10.1 kg]; *n* = 78 male [mean ± SD age 19.2 ± 1.1 years; mean ± SD weight 69.8 ± 13.1 kg]) - light infantry combat soldiers.<br>Control group of female noncombat recruits completing training in the Medical Corps were included for comparison (*n* = 121; mean ± SD age 18.6 ± 0.4 years; mean ± SD weight 57.6 ± 9.5 kg).<br>Due to attrition, final numbers remaining at the end of 14 weeks of training were: 48 female soldiers; 21 male soldiers; 31 female non-combatant soldiers.	haematological factors and stress fractures : upon recruitment – month 0; and following basic training – month 4.	equating to 245.1 cases per 1,000 person-years in the female combatant recruits	Anemia — 28.6% — 17.1%<br>Iron deficiency anaemia — 23.6% — 15.0%<br>**Month 4 – Completion of basic training**<br>**Variable — SF (*n* = 14) — NSF (*n* = 149)**<br>Anemia — 23.1% — 10.0%<br>Iron deficiency anaemia — 23.1% — 8.3%<br><br>*Statistical significance was reported as p < 0.05. Factors for which differences between SF and NSF groups did not reach significance: Transferrin saturation, Ferritin*	
Zhao et al. 2016 [21]<br><br>*Country of origin: China*	Prospective cohort	Male Chinese infantry recruits from three units (N = 1,398), completing 8-weeks of basic military training	Stress fracture diagnoses were made through clinical examination and X-ray findings.<br>Blood samples for markers of bone turnover (bone alkaline phosphatase [BALP]), cross-linked collagen telopeptide [CTX] and N-mid osteocalcin [N-mid OC]) were sampled at baseline.<br>Genomic DNA testing was also conducted to determine associations of stress fractures with GDF5 rs143383.	Overall *case* incidence rate for stress fractures in the Chinese infantry recruits was 135.2 cases per 1,000 recruits over the 8-week basic training period, equating to 878.8 cases per 1,000 person-years.<br><br>***Case* incidence rates for stress fractures in basic training, by anatomical location**<br><br>**Location — Incidence (cases per 1,000 person-years)**<br>Metatarsal — 446.6<br>Tibia — 293.2	**Factors associated with significantly increased risk of stress fractures, and associated odds ratios**<br><br>**Factor — Odds ratio (95% CI)**<br>History of prior fracture (yes vs no) — 1.77 (1.13-2.77)<br>History of <7 h (vs ≥ 7hrs) per week of exercise in previous year — 1.84 (1.32-2.56)<br><br>**Allelic and genotypic frequencies of rs143383 (GDF5) gene in those with (compared to those without) stress fractures. ORs indicate odds of those with, compared to odds of those without, stress fractures having the indicated factor category**	89%<br><br>Level of Evidence: II

Study	Study Design	Participants	Methods (Diagnosis / Exposure to Risk Factors)	Occupations or occupational tasks: comparative levels of incidence or prevalence	Other contextual or risk factors	Study Quality Scores
				Pelvis 60.5<br>Femur 41.6<br>Femoral neck 37.1	**Factor** | **Categories forming basis for OR** | **OR (95% CI),**<br>GDF5 rs143383 allele frequency | T (vs C) allele | 1.75 (1.35-2.28)<br>GDF5 rs143383 genotypic frequency | Codominant TT (vs. CC and TC) | 1.76 (1.29-2.38)<br>| Dominant TT+TC (vs CC) | 2.91 (1.25-6.74)<br>| Recessive TT (vs CC+TC) | 1.83 (1.33-2.52)<br>*There were no significant differences in stress fracture risk levels associated with the examined markers of bone turnover, smoking status, army physical fitness test results, age, height, body weight, or leg length.*	

Methodological quality percentage score is based on the critical appraisal tool specific to the study design, described in the methods section of this review. The levels of evidence are also described in the methods section of this review.

IDF: Israel Defense Forces. Wk: Week. NZ: New Zealand. NZDF: New Zealand Defence Forces. U.S.: United States. OEF: Operation Enduring Freedom. OIF: Operation Iraqi Freedom. OND: Operation New Dawn. OR: Odds Ratio. CI: Confidence Interval. ACSM: American College of Sports Medicine. USMA: United States Military Academy. MRI: Magnetic Resonance Imaging. H/wk: Hours per week. mm: millimetre. mm^2: millimetre squared. ARMS: Assessment of Recruit Motivation and Strength. IRR: Incidence Rate Ratio. Ref: Reference. IR: Incidence Rate. (25(OH)D): Serum 25-hydroxyvitamin D. PTH: Parathyroid Hormone. mmoL: millimoles per litre. BCT: Basic Combat Training. U.K: United Kingdom. MT2: 2nd Metatarsal. MT3: 3rd Metatarsal. FNSF: Displaced Femoral Neck Stress Fracture. PTRP: Physical Training Rehabilitation Program. PCRF: Primary Care Rehabilitation Facility. CIC: Combat Infantry Course. ITC: Infantry Training Centre. IQR: Interquartile Range. RM: Royal Marines. SAI: Shock Absorbing Insole. TAIHOD: Total Army Injury and Health Outcomes Database. NSAID: Non-steroidal anti-inflammatory drugs. SD: Standard Deviation. DMDC: Defence Manpower Data Centre. MEPS: Military Entrance Processing Station. Yrs: Years. BMI: Body Mass Index. DMSS: Defense Medical Surveillance System. USNA: United States Naval Academy. USAFA: United States Air Force Academy. IU: International Units. QUS: Quantative Ultrasound. RR: Relative Risk. SOS: Speed of Sound. HR: Hazard Ratio. SF: Stress Fracture. NSF: Non-Stress Fracture. AFHSC: Armed Forces Health Surveillance Center. UV: Ultra Violet. cm: Centimetres. Min/session: Minutes per Session. TAPAS: Tailored Adaptative Personality Assessment System. SUFV: Special Unit Fighting Vest. NFV: New Fighting Vest. MCRD: Marine Corps Recruit Depot. TDE: Training Day Exposures. LE: Lower Extremity. UE: Upper Extremity. TRACP-5b: Serum tartrate acid phosphatase isoform 5b. DPD: Deoxypyridinoline. CR: Creatinine. BALP: Bone Alkaline Phosphatase. CTX: Collagen Telopeptide. N-mid OC: N-mid osteocalcin. DNA: Deoxyribonucleic acid. Hx: History. Avg: Average.
