# Supplementary material for: Incidence and Risk Factors for the Development of Stress Fractures in Military Recruits and Qualified Personnel: A Systematic Review
Source: Int J Environ Res Public Health. 2025 Nov 20;22(11):1760. doi: 10.3390/ijerph22111760 (PMC12652357; doi:10.3390/ijerph22111760)
Supplement: Supplementary file 1 [file ijerph-22-01760-s001.zip › Supplementary Material File S5.pdf]

Supplementary Material S5: Characteristics and key findings of studies of stress fractures in qualified military personnel and studies where recruit and qualified military populations could not be separated due to the time-periods of observation

<table>
<tr><th>Study</th><th>Study Design</th><th>Participants</th><th>Methods (Diagnosis / Exposure to Risk Factors)</th><th>Occupations or occupational tasks: comparative levels of incidence or prevalence</th><th>Other contextual or risk factors</th><th>Study Quality Scores</th></tr>
<tr>
<td>Ben-Ami et al. 2018 [7]

Country of origin: Israel</td>
<td>Retrospective cohort</td>
<td>100,000 randomly selected Israeli Defence Force combat soldiers starting and finishing military service in the period 1/01/2005 – 31/12/2015.</td>
<td>Randomly sampled soldiers' medical records were queried for exposure information relating to ADHD diagnosis and use of methylphenidate.
Three cohorts were then formed: subjects with treated (methylphenidate) ADHD (n = 689); subjects with untreated ADHD (n = 762); and control group with no ADHD (n = 98,549). The treated ADHD group had a proportion of females almost double that of the other two cohorts (13.9% vs 7.5% and 5.9%).
ICD-9 codes relating to stress fracture diagnoses were used to calculate incidence.</td>
<td>Overall incidence (new stress fracture diagnoses per 1,000 person-years) of stress fractures for control group was 56.88

Control group: Stress fracture incidence by body location (new stress fracture diagnoses per 1,000 person-years)
<table>
<tr><th>Location</th><th>Control Group</th></tr>
<tr><td>Tibia & Fibula</td><td>46.20</td></tr>
<tr><td>Metatarsals</td><td>6.60</td></tr>
<tr><td>Femoral shaft</td><td>2.76</td></tr>
<tr><td>Femoral neck</td><td>0.60</td></tr>
<tr><td>Pelvis</td><td>0.48</td></tr>
</table></td>
<td>Overall incidence (new stress fracture diagnoses per 1,000 person-years) of stress fractures, by ADHD treatment status
<table>
<tr><th>Treated ADHD</th><th>Untreated ADHD</th></tr>
<tr><td>81.96</td><td>62.76</td></tr>
</table>
Adjusted OR for stress fractures
<table>
<tr><th>Factor</th><th>OR (95% CI)</th></tr>
<tr><td>Treated ADHD</td><td>1.04 (1.02-1.07)</td></tr>
<tr><td>Untreated ADHD</td><td>1.01 (0.99-1.03)</td></tr>
<tr><td>Control</td><td>1.00 (Reference)</td></tr>
<tr><td>Female</td><td>1.06 (1.05-1.07)</td></tr>
<tr><td>Male</td><td>1.00 (Reference)</td></tr>
<tr><td>Anaemia – Yes</td><td>1.05 (1.04-1.06)</td></tr>
<tr><td>Anaemia – No</td><td>1.00 (Reference)</td></tr>
<tr><td>Weight (per additional kg)</td><td>0.99 (0.985-0.995)</td></tr>
<tr><td>Age (per additional year)</td><td>0.994 (0.991-0.997)</td></tr>
<tr><td>Duration of service (per each additional month)</td><td>1.0009 (1.0006-1.0010)</td></tr>
</table>
Stress fracture incidence by body location (new stress fracture diagnoses per 1,000 person-years)
<table>
<tr><th>Location</th><th>Treated ADHD</th><th>Untreated ADHD</th></tr>
</table></td>
<td>67%

Level of Evidence: III-2</td>
</tr>
</table>

Study	Study Design	Participants	Methods (Diagnosis / Exposure to Risk Factors)	Occupations or occupational tasks: comparative levels of incidence or prevalence	Other contextual or risk factors	Study Quality Scores
					Tibia & Fibula — 70.08 — 58.32<br>Metatarsals — 9.72 — 9.48<br>Femoral shaft — 5.16 — 3.6<br>Femoral neck — 1.32 — 0.60<br>Pelvis — 1.32 — 0.60	
Bulathsinhala et al. 2017 [9]<br><br>*Country of origin: United States of America*	Retrospective cohort	Active-duty U.S. Army soldiers from 2001-2011 (N = 1,299,332).	Data were extracted from the Total Army Injury and Health Outcomes Database (TAIHOD) using medical encounters records and ICD-9 codes relating to stress fractures (733.93 – 733.98).	**Overall incidence rate for stress fractures in U.S. Army soldiers from 2001-2011 was 4.12 stress fractures per 1,000 person-years**	**Overall incidence rate for stress fractures in male U.S. Army soldiers from 2001-2011 was 2.05 stress fractures per 1,000 person-years**<br>**Overall incidence rate for stress fractures in female U.S. Army soldiers from 2001-2011 was 7.45 stress fractures per 1,000 person-years**<br>**Incidence rate ratio for stress fractures, female: male: 3.63**<br><br>**Incidence rates for stress fractures by race-origin category (stress fractures per 1,000 person-years)**<br>**Race-Origin — Incidence rate**<br>Non-Hispanic black — 2.72<br>Non-Hispanic white — 4.72<br>Hispanic — 4.10<br>American Indian/native Alaskan — 5.11<br>Asian — 3.21<br>Native Hawaiian/Pacific Islander — 3.06<br>Mixed races — 5.41<br><br>**Adjusted Hazard Ratios (HR) for stress fractures, comparing race-origin categories, stratified by sex**<br>**Race-origin — HR for Males — HR for Females**	89%<br><br>Level of Evidence: III-2

Study	Study Design	Participants	Methods (Diagnosis / Exposure to Risk Factors)	Occupations or occupational tasks: comparative levels of incidence or prevalence	Other contextual or risk factors	Study Quality Scores
					Non-Hispanic black: 1.00 (Reference); 1.00 (Reference)<br>Non-Hispanic white: 1.59 (1.49-1.68); 1.92 (1.81-2.03)<br>Hispanic: 1.19 (1.10-1.29); 1.65 (1.53-1.79)<br>American Indian/native Alaskan: 1.15 (0.93-1.43); 1.72 (1.44-2.05)<br>Asian: 1.06 (0.94-1.19); 1.32 (1.16-1.49)<br>Native Hawaiian / Pacific Islander: 0.73 (0.52-1.03); 1.18 (0.90-1.55)<br>Mixed races: 1.42 (0.78-2.57); 1.58 (0.85-2.95)	
Claassen, Hu & Rohrbeck (2014) [13]<br><br>*Country of origin: United States of America*	Retrospective cohort	Active U.S. military service members (Army, Navy, Air Force, Marine Corps, Coast Guard), recruits, and deployed members to OEF, OIF or OND. Surveillance period for active and recruit cohorts was 1/01/2003 – 31/12/2012; deployed was 1/01/2008 – 31/12/2012. Coast guard periods were 2007 – 2012.	Data from the Defense Medical Surveillance System, Theater Medical Data Store, and Transportation Command Regulating and Command and Control Evacuation System were queried for incident ICD-9 codes for fracture types: stress, pathologic, head, vertebra, ribs, sternum, larynx and trachea, pelvis, arm, hand, leg, foot/ankle, unspecified.<br><br>Stress fracture data for qualified military personnel only are presented here.	Active-duty personnel: stress fracture overall incidence: 2.7 stress fractures per 1,000 person-years<br>Deployed personnel: stress fracture overall incidence: 0.7 stress fractures per 1,000 person-years<br>**Stress fracture incidence (stress fractures per 1,000 person-years) by anatomical location, in active service members and deployed personnel**<br>**Location** / **Active** / **Deployed**<br>Tibia / fibula: 1.2; 0.2<br>Metatarsals: 0.4; 0.2<br>Other bone: 0.9; 0.3<br>Femoral neck: 0.1; 0.0<br>Femoral shaft: 0.0; 0.0<br>Pelvis: 0.1; 0.0		67%<br><br>Level of Evidence: III-2

Study	Study Design	Participants	Methods (Diagnosis / Exposure to Risk Factors)	Occupations or occupational tasks: comparative levels of incidence or prevalence	Other contextual or risk factors	Study Quality Scores
Fedgo & Stahlman (2020) [30]<br><br>*Country of origin: United States of America*	Retrospective cohort (with nested case-control	Active component members of the U.S. Army, Air Force, Navy, or Marine Corps serving between 1/06/2014 – 31/12/2018 – total number not reported. Cases of incident stress fractures were identified throughout the study period (*n* = 7036) and each matched with up to four controls (*n* = 28141).	The Defense Medical Surveillance System was used to identify cases of incident stress fractures, pharmacy data relating to NSAIDs, and data relating to vitamin D deficiency with hospitalisation or ambulatory encounters.<br>Incident stress fractures were identified with ICD-9 (733.93-.98) or ICD-10 (M84.371-.379, M84.30-.38, M84.344-0.36, M84.51-.53) codes or through hospitalisation data with diagnosis code for stress fracture. Vitamin D deficiency was identified using ICD-9 code E55.9. To identify individuals who had been prescribed NSAIDs 30-180 days prior to the case-control study reference date, prescription records were queried for American Hospital Formulatory Service (AFHS) therapeutic class code 280804.<br><br>NSAID as the main exposure variable was defined as intake of an NSAID 30-180 days prior to diagnosis of stress fracture. Included NSAID classes were propionic acid derivatives, salicylates, preferential cox-2 inhibitors, indole derivatives, aryl acetic acid derivatives, anthranillic acid derione). mn		Service members who had intake of NSAIDs in the designated period demonstrated an adjusted* incidence rate ratio for stress fracture diagnoses of 1.70 (95% CI, 1.58-1.82) when compared to members who had not received NSAIDs.<br><br>When the analysis was restricted to solely the Army population, the incidence rate ratio was 1.64 (95% CI, 1.49-1.80).<br><br>**adjusted for sex, race/ethnicity, service, age, time in service, recruit status, occupation, and diagnosis of vitamin D deficiency*	67%<br><br>Level of Evidence: III-2

Study	Study Design	Participants	Methods (Diagnosis / Exposure to Risk Factors)	Occupations or occupational tasks: comparative levels of incidence or prevalence	Other contextual or risk factors	Study Quality Scores
Finestone et al. 2014 [34]<br><br>*Country of origin: Israel*	Prospective cohort	Three consecutive inductions of male (n=85) and female (n=238) Karakal (light infantry) recruits (September 2004 – July 2005) completing basic training (14 weeks) and subsequent service, totalling 36 months	Recruits were examined by orthopaedic surgeons every 2-3 weeks for the duration of their basic training and subsequent stress fractures, occurring during subsequent service, were identified from central medical records. Stress fractures were confirmed by radiography and/or bone scans.	Calculated overall 36-month incidence of stress fractures (all types) diagnosed in the male and female recruits (combined) was 16.1% (95% CI 12.5-20.5%), equating to an incidence rate of 53.7 stress fractures per 1,000 person-years of military service	36-month incidence of stress fractures (all types) diagnosed in females was 21.0% (95% CI 16.2-26.5%), equating to an incidence rate of 70.0 stress fractures per 1,000 person-years of military service<br>36-month incidence of total stress fractures diagnosed for males was 2.35% (95% CI, 0.3-8.2%), equating to an incidence rate of 7.8 stress fractures per 1,000 person-years of military service<br>Calculated stress fracture incidence rate ratio (female:male) = 8.93 (95% CI 2.22-35.90)<br>**Stress fracture incidence rates over 36 months of service, by anatomical location and sex (stress fractures per 1,000 person-years)**<br>**Location / Male / Female**<br>Femoral 3.9 23.7<br>Tibia 3.9 40.7<br>Fibular 0 4.3<br>Metatarsal 0 14.0<br>Other 0 5.7	89%<br><br>Level of Evidence: II
Hughes et al. 2019 [44]<br><br>*Country of origin: United States of America*	Cohort study with nested case-control study, which used cohort data from the underlying cohort study to inform derivation of incidence rate ratios from the case-control element	Two separate cohorts. Cohort one was the full U.S. Army population from 2002 – 2011 (with *n* = 24,146 stress fracture cases in that time period). Cohort two was the subset of individuals in the Army at each timepoint in the study period that was undertaking the 11-week basic combat training course (BCT-only subgroup). The BCT-subgroup experienced *n* = 9088 reported stress fractures during the time period of the study.	Cases of incident stress fractures were identified through the Total Army Injury and Health Outcomes Database (TAIHOD) using ICD-9 codes (i.e. 733.14-.16, 733.94-.98). Selection of controls was based on matching each stress fracture case to four control soldiers (no history of stress fracture), with controls matched for date of occurrence of case's injury and length of time within army service.<br><br>Frequency of non-steroidal anti-inflammatory drugs intake by individuals within each cohort (Army and BCT-only subgroup) was the main exposure variable and defined as intake of an NSAID 30-180 days prior to diagnosis of stress fracture.		**Incidence rate ratios for stress fractures comparing incidence in those with NSAID or paracetamol use to those without NSAID or paracetamol use (sex-adjusted)**<br>**Prescription / Army (IRR, 95% CI)**<br>All NSAIDs 2.9 (2.8-2.9)<br>Ibuprofen 2.2 (2.1-2.3)<br>Naproxen 2.6 (2.5-2.7)<br>Indomethacin 2.1 (1.8-2.3)<br>Paracetamol 2.1 (2.0-2.2)<br><br>**Incidence rate ratios for stress fractures in the full army population, comparing those with NSAID or paracetamol use unrelated to MSK injury or pain to those without such use (sex-adjusted)**	100%<br><br>Level of Evidence: III-2

Study	Study Design	Participants	Methods (Diagnosis / Exposure to Risk Factors)	Occupations or occupational tasks: comparative levels of incidence or prevalence	Other contextual or risk factors	Study Quality Scores
			NSAID use was extracted from medical records of soldiers.<br><br>Recorded NSAIDs included ibuprofen, naproxen, meloxicam, and indomethacin. Use of paracetamol (acetaminophen) was also included.		**Prescription** / **IRR (95% CI) for full Army population**<br>All NSAIDs 1.74 (1.66-1.82)<br>Ibuprofen 1.61 (1.53-1.69)<br>Naproxen 1.89 (1.73-2.07)<br>Indomethacin 1.50 (1.16-1.95)<br>Paracetamol 1.36 (1.27-1.46)<br><br>**Lagged analysis model* (Army population)**<br>**Time-lag** / **Prescription** / **IRR (95% CI)**<br>**15-day**: All NSAIDs 3.9 (3.7-4.0); Ibuprofen 2.7 (2.6-2.8); Naproxen 3.1 (2.9-3.2); Indomethacin 2.5 (2.2-2.8); Paracetamol 2.3 (2.3-2.4)<br>**30-day**: All NSAIDs 2.9 (2.8-2.9); Ibuprofen 2.2 (2.1-2.3); Naproxen 2.6 (2.5-2.7); Indomethacin 2.1 (1.8-2.3); Paracetamol 2.1 (2.0-2.2)<br>**45-day**: All NSAIDs 2.4 (2.3-2.5); Ibuprofen 2.0 (1.9-2.0); Naproxen 2.3 (2.2-2.4); Indomethacin 2.0 (1.7-2.3); Paracetamol 1.9 (1.8-2.0)<br>* The lagged analysis model constituted a sensitivity analysis, where IRRs if NSAIDs were prescribed within 15-180 days or 45-180 days prior to stress fracture onset were compared to the IRRs from the primary analysis (NSAID use 30-180 days prior to stress fracture onset).	
Johnson et al., 2024 [47]<br><br>Country of Origin: United States	Retrospective Cohort	US Air Force Special Warfare trainees (N = 2,290; *n* = 2,278 males; *n* = 12 females) entering an 8-week (120 day) training period between	Injury data were obtained from Medical Health System Mart.<br><br>Initially, health records were queried for encounters with relevant ICD10 codes followed by a review to verify the individual had imaging results to	*Case* incidence rate of 1.41 lower extremity BSI per 100 person-months for the total cohort. This equates to a *case* incidence rate for BSI of 164.7 cases per 1,000 person-years for the total cohort.<br><br>**BSI by Anatomical Location**	The *case* incidence rate for BSI for males was calculated as 163.1 per 1,000 person-years; and for females the incidence was calculated as 506.3 cases per 1,000 person-years. This equates to an IRR of 3.11 (Females:Males).<br>**Logistic regression analysis (all factors included in model, which was adjusted for treatment)**	82%<br><br>Level of Evidence: III-2

Study	Study Design	Participants	Methods (Diagnosis / Exposure to Risk Factors)	Occupations or occupational tasks: comparative levels of incidence or prevalence	Other contextual or risk factors	Study Quality Scores
		October 2017 – May 2021.	confirm the diagnosis of a bone stress injury (BSI).	**Anatomical Location** — **% LE BSIs**<br>Tibia/Fibula — 63%<br>Foot — 22%<br>Hip/Thigh — 12%<br>Ankle — 3%	Physical abilities stamina test 2-min sit-ups — OR = 0.956 (95% CI 0.927-0.987) — *p* = 0.006<br>Prior hx of high impact sports — OR = 0.361 (95% CI 0.203-0.641) — *p* = 0.001<br>Prior lower-leg MSKI — OR = 4.830 (95% CI 2.1260-9.418) — *p* <0.001<br>Sample included n = 1289 – entered after June 16, 2019<br>High impact sports: Baseball, basketball, cross country, football, gymnastics, lacrosse, martial arts, track & field, rugby, soccer, tennis	
MacGregor et al., 2022 [66]<br><br>Country of Origin: United States	Retrospective Cohort	All active duty and reserve military service members in the U.S Armed Forces (Army, Navy, Marine Corps or Air Force) between 2006-2015.	The Defense Medical Epidemiology Database (DMED) was queried for ICD-9 codes relating to Ankle-Foot Complex (AFC) stress fractures (733.93 – 733.94).	Overall incidence of AFC stress fractures across all branches of service was 3.20 per 1,000 person-years.	Overall incidence of AFC stress fractures across all branches of service for male personnel only was 2.76 per 1,000 person-years; and for females was 5.78 per 1,000 person-years. This equates to a female:male IRR of 2.10.<br><br>**Sex-specific incidence rates (AFC stress fractures per 1,000 person-years) for enlisted personnel by branch of service and sex**<br>**Branch of Service** — **Male** — **Female** — **p-value**<br>Air Force — 1.7 — 3.7 — <0.001<br>Army — 4.0 — 8.6 — <0.001<br>Marine Corps — 4.8 — 11.8 — <0.001<br>Navy — 1.6 — 5.8 — <0.001<br>**Sex-specific incidence rates (AFC stress fractures per 1,000 person-years) for officers by branch of service and sex**<br>**Branch of Service** — **Male** — **Female** — **p-value**<br>Air Force — 0.8 — 2.1 — <0.001<br>Army — 1.5 — 2.5 — <0.001<br>Marine Corps — 2.0 — 5.5 — <0.001<br>Navy — 0.7 — 1.7 — <0.001	91%<br><br>Level of Evidence: III-2

Study	Study Design	Participants	Methods (Diagnosis / Exposure to Risk Factors)	Occupations or occupational tasks: comparative levels of incidence or prevalence	Other contextual or risk factors	Study Quality Scores
					(see nested table below)	

Integrated (male & female) and non-integrated enlisted occupations AFC stress fracture incidence

Occupation	Incidence	p-value
Total male – integrated	2.9	<0.001
Total female – integrated	6.6	<0.001
**Non-integrated enlisted occupations AFC stress fracture incidence**		
Special forces	0.8	<0.001
Infantry	5.0	<0.001
Mechanised/armour	2.4	<0.001

Relative risk of AFC stress fracture for female personnel (comparative to males) in integrated occupations

Occupation	RR (95% CI)	p-value
	**Enlisted**	
Artillery/gunnery	5.15 (4.62-5.75)	<0.001
Aviation	5.74 (4.80-6.87)	<0.001
Engineers	3.16 (2.56-3.90)	<0.001
Maintenance	2.91 (1.83-2.02)	<0.001
Administration/Intelligence/Communication	1.91 (1.83-2.02)	<0.001
Logistics	2.87 (2.67-3.08)	<0.001
Maritime/Naval Specialities	2.41 (2.04-2.85)	<0.001
Training	3.74 (2.87-4.87)	<0.001
	**Officer**	
Ground and Naval Gunfire	0.95 (1.61-1.49)	0.84
Aviation	2.33 (1.52-3.55)	<0.001

Study	Study Design	Participants	Methods (Diagnosis / Exposure to Risk Factors)	Occupations or occupational tasks: comparative levels of incidence or prevalence	Other contextual or risk factors	Study Quality Scores
					Engineering and Maintenance | 2.14 (1.71-2.68) | <0.001<br>Administration | 1.95 (1.51-2.51) | <0.001<br>Operations and Intelligence | 2.27 (1.75-2.96) | <0.001<br>Logistics | 2.28 (1.77-2.94) | <0.001<br>Services | 2.08 (1.80-2.40) | <0.001<br>Training | 1.79 (1.33-2.40) | <0.001	
Potter et al. 2002 [94]<br><br>*Country of origin: United States of America*	Prospective cohort	U.S. Army soldiers of the 82nd Airborne Division, 1/04/1996 – 31/03/1997, N=1965. Includes two infantry battalions (*n* = 669), and three combat support battalions (*n* = 1,180 men; *n* = 116 women).	Medical records of activity duty soldiers were reviewed to identify radiographically confirmed or clinically suspected stress fracture cases, with both included in the reported stress fracture incidence rates reported here.	Overall incidence of stress fractures was 1.8 (95% CI, 1.3-2.5) stress fractures per 1,000 soldier months, equating to 21.6 stress fractures per 1,000 person-years	Incidence of stress fractures for women was 4.2 (95% CI, 1.5-10) stress fractures per 1,000 soldier months, equating to 50.4 stress fractures per 1,000 person-years<br><br>Incidence of stress fractures for men was 1.7 (95% CI, 1.2-2.4) stress fractures per 1,000 soldier months, equating to 20.4 stress fractures per 1,000 person-years<br><br>Incidence rate ratio for stress fractures, female: male, was 2.47	89%<br><br>Level of Evidence: II
Waterman et al. 2016 [117]<br><br>*Country of origin: United States of America*	Retrospective cohort	U.S. Armed Forces (Army, Navy, Air Force, Marines) service members 2009 – 2012.	Defense Medical Epidemiology Database (DMED) queried for ICD-9 codes relating to stress fractures of the lower extremities (e.g., 733.93 – 733.97) occurring 2009 – 2012.	**Overall incidence of stress fractures from 2009 – 2012 was 5.69 stress fractures per 1,000 person-years**<br><br>**Overall incidence (stress fractures per 1,000 person-years) of stress fractures by anatomical location**<br><br>**Location** | **Incidence rate**<br>Femoral neck | 0.49<br>Tibia | 2.26	**Adjusted incidence rate ratios for stress fractures, by age category**<br><br>**Category** | **Age (years)** | **Adjusted incidence rate ratio***<br>| < 20 | 3.14 (3.05-3.23)<br>**Overall** | 20-24 | 1.00 (reference)<br>| 25-29 | 1.15 (1.11-1.19)	89%<br><br>Level of Evidence: III-2

Study	Study Design	Participants	Methods (Diagnosis / Exposure to Risk Factors)	Occupations or occupational tasks: comparative levels of incidence or prevalence			Other contextual or risk factors			Study Quality Scores
				Metatarsal		0.92		30-34	1.47 (1.40-1.54)	
				Other		1.68		35-39	2.01 (1.90-2.13)	
				Femoral shaft		0.34		≥ 40	6.4 (6.12-6.70)	
								< 20	2.22 (2.01-2.45)	
				**Adjusted incidence rate ratios for stress fractures, by branch of service**				20-24	1.00 (reference)	
				**Category**	**Branch**	**Adjusted incidence rate ratio***	**Femoral Neck**	25-29	1.09 (0.98-1.22)	
					Army	2.56 (2.47-2.65)		30-34	1.3 (1.11-1.54)	
				**Total**	Marines	1.9 (1.82-1.98)		35-39	1.82 (1.49-2.22)	
					Air Force	1.09 (1.04-1.13)		≥ 40	6.69 (5.75-7.78)	
					Navy	1.00 (reference)		< 20	3.07 (2.93-3.22)	
					Army	6.19 (5.27-7.26)		20-24	1.00 (reference)	
				**Femoral Neck**	Marines	4.28 (3.57-5.12)	**Tibial**	25-29	1.14 (1.08-1.20)	
					Air Force	1.06 (0.86-1.32)		30-34	1.52 (1.41-1.64)	
					Navy	1.00 (reference)		35-39	1.92 (1.75-2.10)	
				**Tibial**	Army	1.84 (1.75-1.94)		≥ 40	6.61 (6.15-7.12)	
					Marines	1.34 (1.26-1.43)	**Adjusted for sex, race, rank, branch of service and calendar year*			

Study	Study Design	Participants	Methods (Diagnosis / Exposure to Risk Factors)	Occupations or occupational tasks: comparative levels of incidence or prevalence	Other contextual or risk factors	Study Quality Scores
				(see below)	(see below)	

Occupations or occupational tasks: comparative levels of incidence or prevalence (continued)

Air Force	0.91 (0.86-0.98)
Navy	1.00 (reference)

**Adjusted for sex, race, rank, age and calendar year*

Adjusted incidence rate ratios for stress fractures, by Rank category

	Category	
**Overall**	Junior Enlisted	18.54 (16.97-20.26)
	Junior Officer	3.89 (3.53-4.29)
	Senior officer	1.00 (reference)
	Junior Enlisted	29.76 (21.23-41.72)
**Femoral Neck**	Senior Enlisted	2.11 (1.49-2.99)
	Junior Officer	3.04 (2.05-4.49)
	Senior officer	1.00 (reference)
	Junior Enlisted	30.76 (25.97-36.44)
**Tibial**	Senior Enlisted	4.84 (4.11-5.72)
	Junior Officer	5.63 (4.69-6.75)
	Senior officer	1.00 (reference)

Other contextual or risk factors

Adjusted incidence rate ratios for stress fractures, by race category

**Rank**	**Adjusted incidence rate ratio***	**Adjusted incidence rate ratio***
**Total**	White	1.51 (1.46-1.55)
Senior Enlisted	3.2 (2.94-3.49)	1.17 (1.12-1.22)
	Black	1.00 (reference)
	White	1.88 (1.68-2.09)
**Femoral Neck**	Other	1.11 (0.94-1.31)
	Black	1.00 (reference)
	White	1.18 (1.12-1.23)
**Tibial**	Other	1.08 (1.01-1.15)
	Black	1.00 (reference)

**Adjusted for sex, age, rank, branch of service and calendar year*

Study	Study Design	Participants	Methods (Diagnosis / Exposure to Risk Factors)	Occupations or occupational tasks: comparative levels of incidence or prevalence	Other contextual or risk factors	Study Quality Scores
				**Adjusted for sex, race, age, rank, and calendar year*	**Adjusted incidence rate ratios for stress fractures, by sex**<br><br>**Category** / **Sex** / **Adjusted incidence rate ratio***<br>**Overall**: Male 1.00 (reference); Female 3.11 (3.03-3.18)<br>**Femoral Neck**: Male 1.00 (reference); Female 7.13 (6.60-7.71)<br>**Tibial**: Male 1.00 (reference); Female 2.3 (2.21-2.40)<br><br>**Adjusted for branch of service, race, age, rank, and calendar year*	

Methodological quality percentage score is based on the critical appraisal tool specific to the study design, described in the methods section of this review. The levels of evidence are also described in the methods section of this review.

U.S.: United States. TAIHOD: Total Army Injury and Health Outcomes Database. HR: Hazard Ratio. OEF: Operation Enduring Freedom. OIF: Operation Iraqi Freedom. OND: Operation New Dawn. NSAID: Non-steroidal anti-inflammatory drugs. AFHS: American Hospital Formulatory Service. MSK: Musculoskeletal. IRR: Incidence Rate Ratio. DMED: Defense Medical Epidemiology Database. USMA: United States Military Academy. ADHD: Attention deficit hyperactivity disorder. Kg: Kilogram. BCT: Basic Combat Training.
